# Supplementary material for: Rapid and accurate prediction of protein homo-oligomer symmetry using Seq2Symm
Source: Nat Commun. 2025 Feb 27;16:2017. doi: 10.1038/s41467-025-57148-3 (PMC11868566; doi:10.1038/s41467-025-57148-3)
Supplement: Supplementary file 1 — Supplementary Information [file 41467_2025_57148_MOESM1_ESM.pdf]

Supplementary Figures

Supplementary Figure 1. (a) We show AUC-PR on the validation set across all methods **(b)** Weighted AUC-PR on the validation and test set, where the number of structures from each class are used to weight the average. Majority classes like C1 which constitute 40% of the dataset dominate the performance metrics.

(a) Validation set AUC-PR

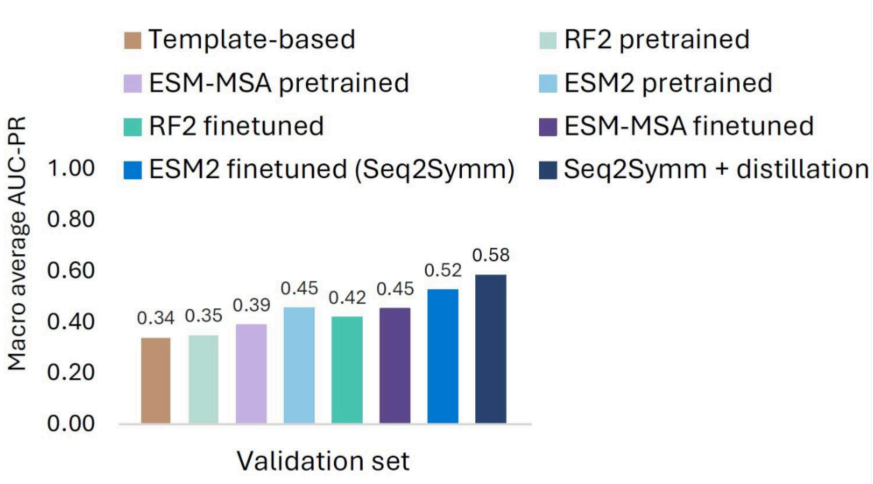

(b) Weighted AUC-PR

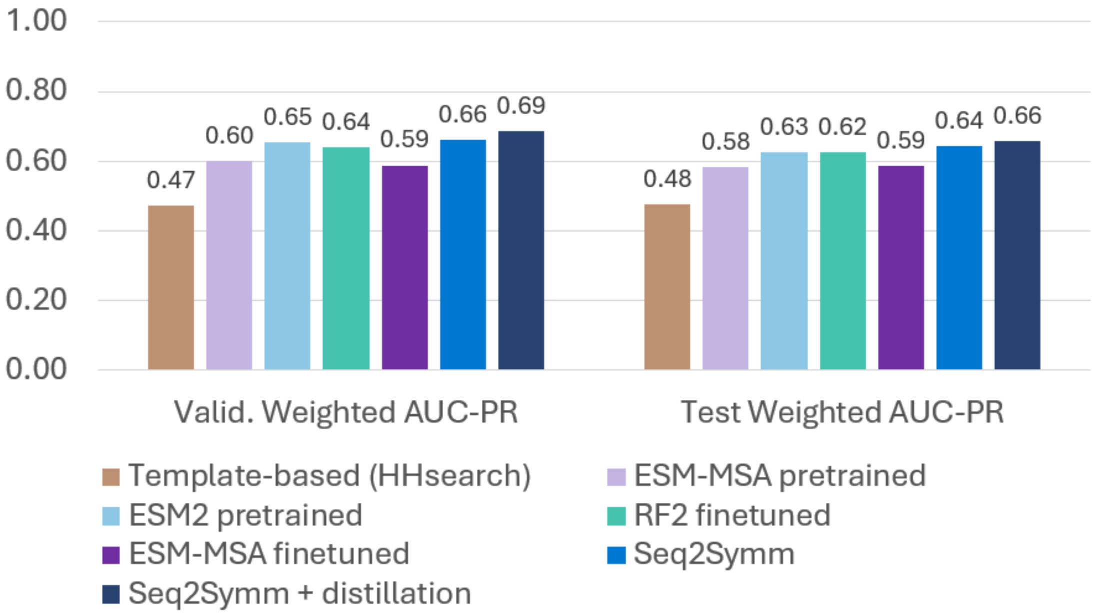

Supplementary Figure 2. Class-wise precision recall curves on the validation set. Seq2Symm is shown as ‘ESM2 finetuned’ and ‘ESM2 ft distilled’ is Seq2Symm with distillation.

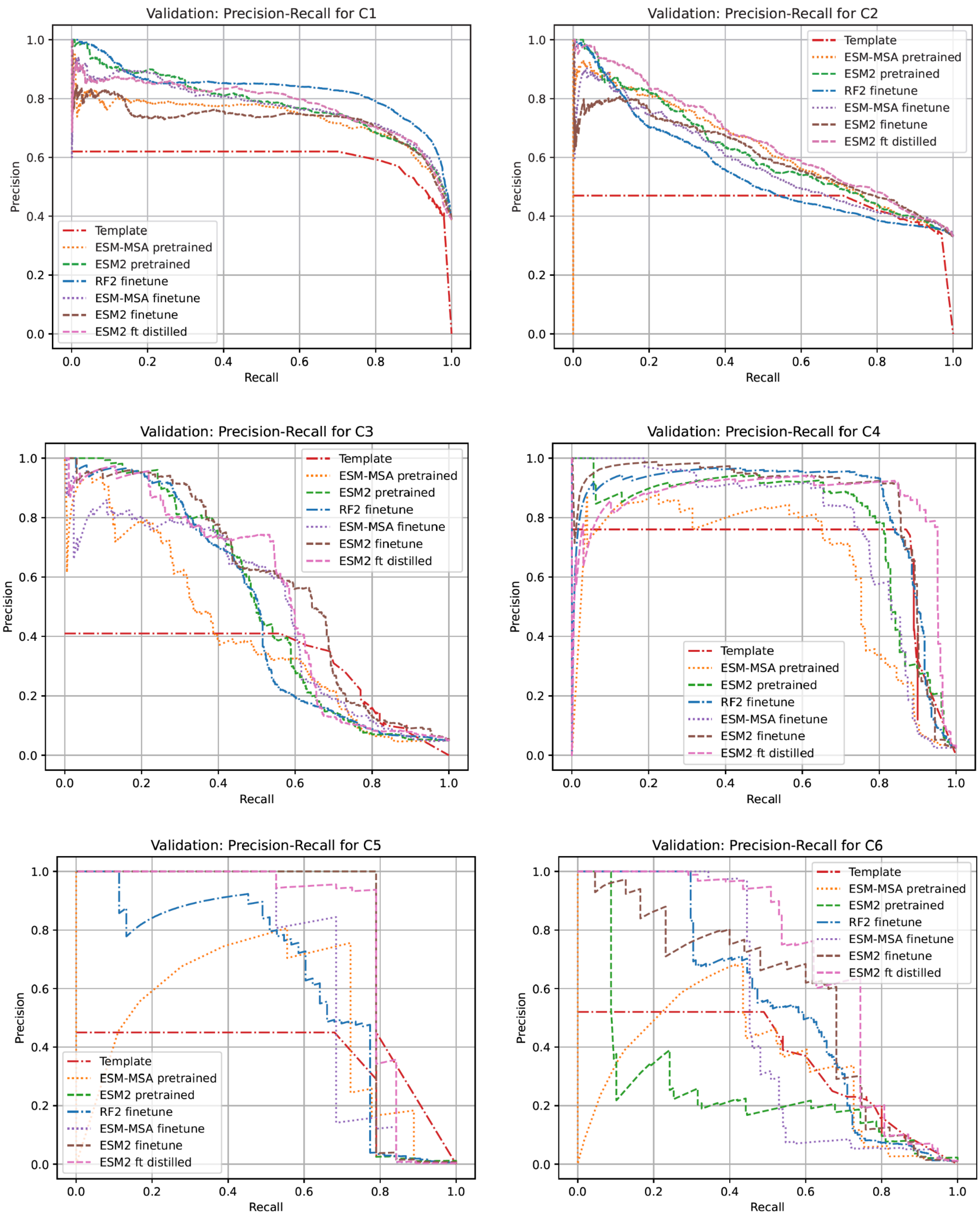

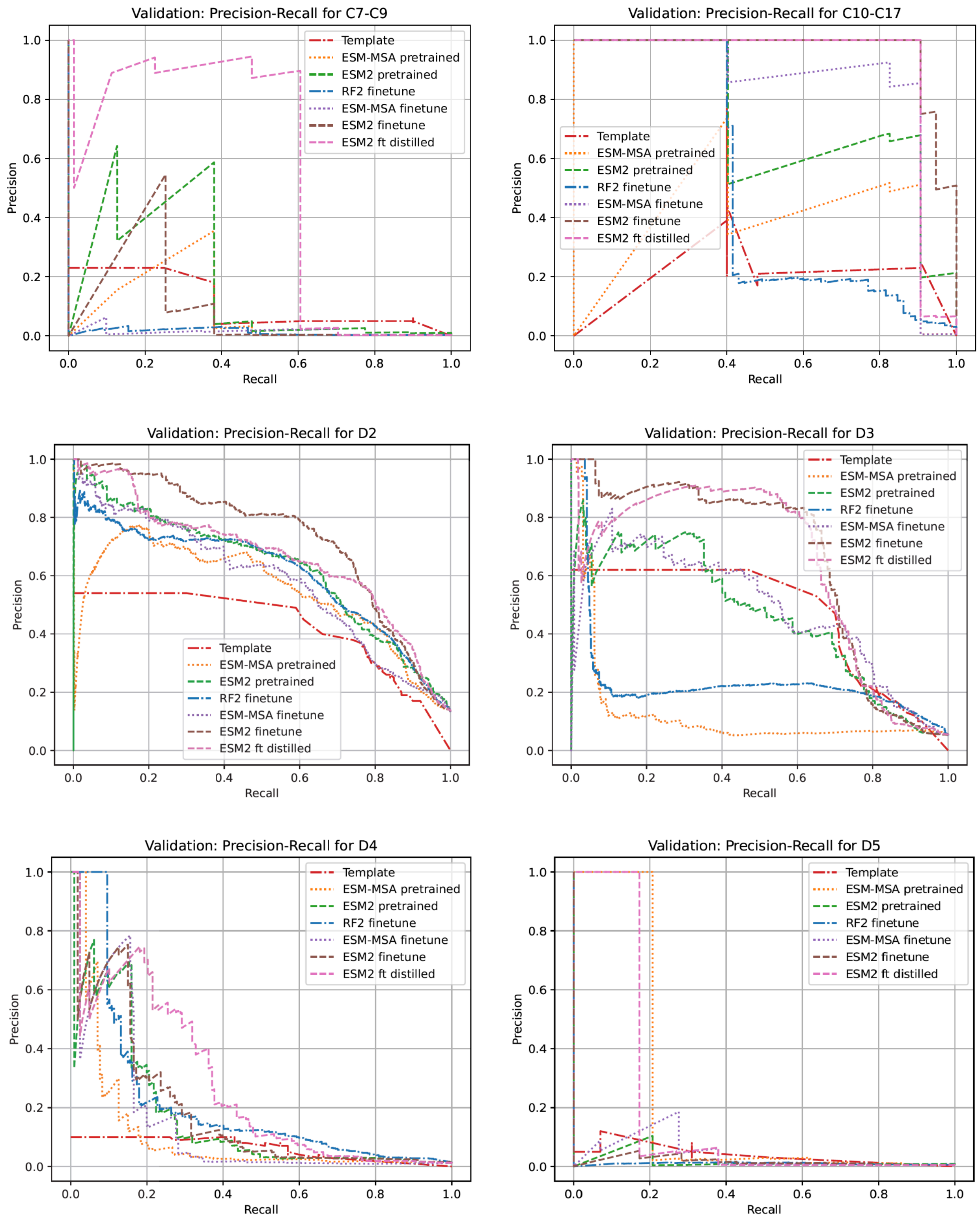
Validation: Precision-Recall for C7-C9
Validation: Precision-Recall for C10-C17
Validation: Precision-Recall for D2
Validation: Precision-Recall for D3
Validation: Precision-Recall for D4
Validation: Precision-Recall for D5
Precision
Recall
0.0
0.2
0.4
0.6
0.8
1.0
Template
ESM-MSA pretrained
ESM2 pretrained
RF2 finetune
ESM-MSA finetune
ESM2 finetune
ESM2 ft distilled

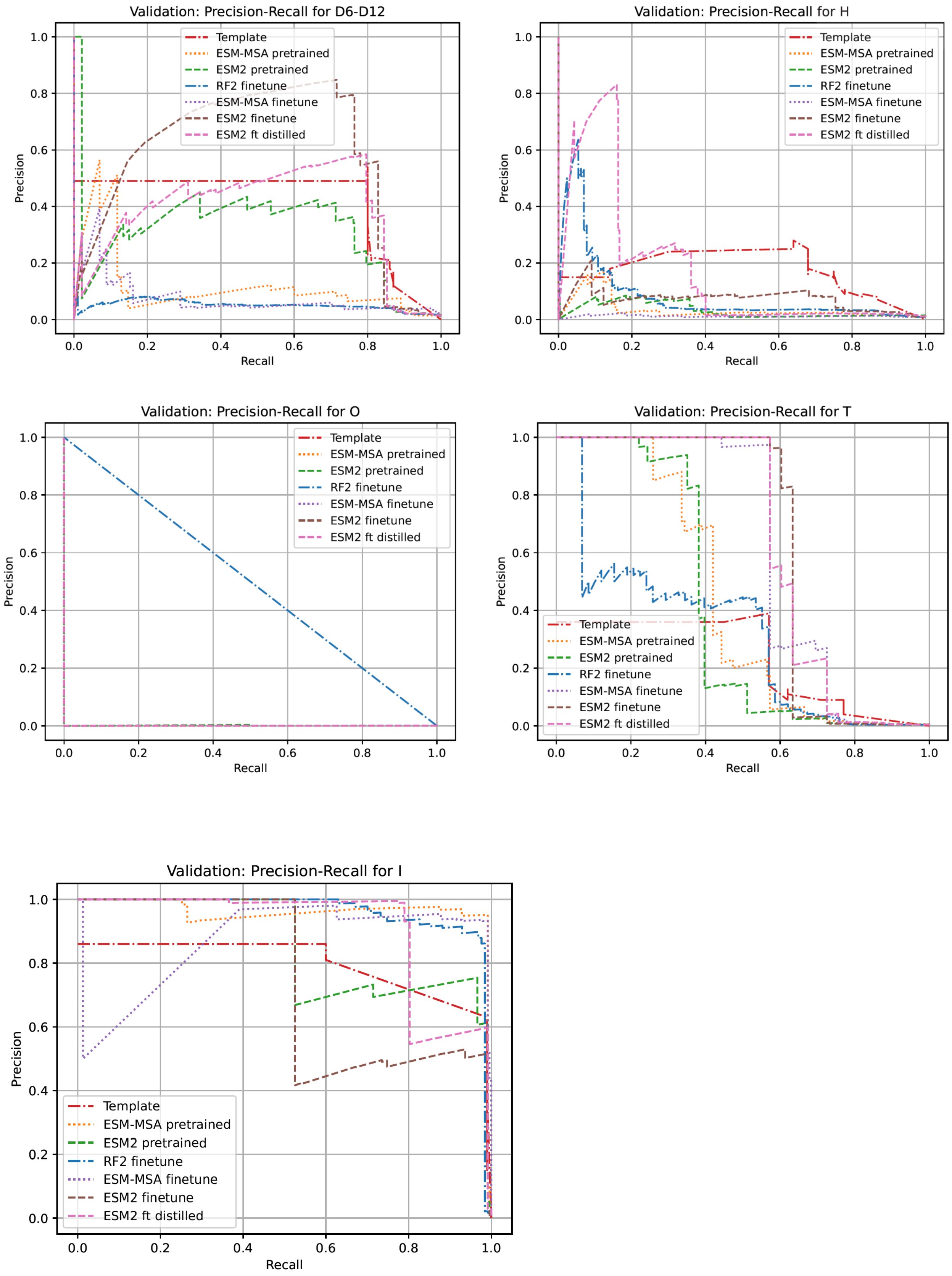
Validation: Precision-Recall for D6-D12
Validation: Precision-Recall for H
Validation: Precision-Recall for O
Validation: Precision-Recall for T
Validation: Precision-Recall for I
Precision
Recall
Template
ESM-MSA pretrained
ESM2 pretrained
RF2 finetune
ESM-MSA finetune
ESM2 finetune
ESM2 ft distilled

Supplementary Figure 3. Class-wise precision recall curves on the unseen test dataset. Seq2Symm is shown as 'ESM2 finetuned' and 'ESM2 ft distilled' is Seq2Symm with distillation.

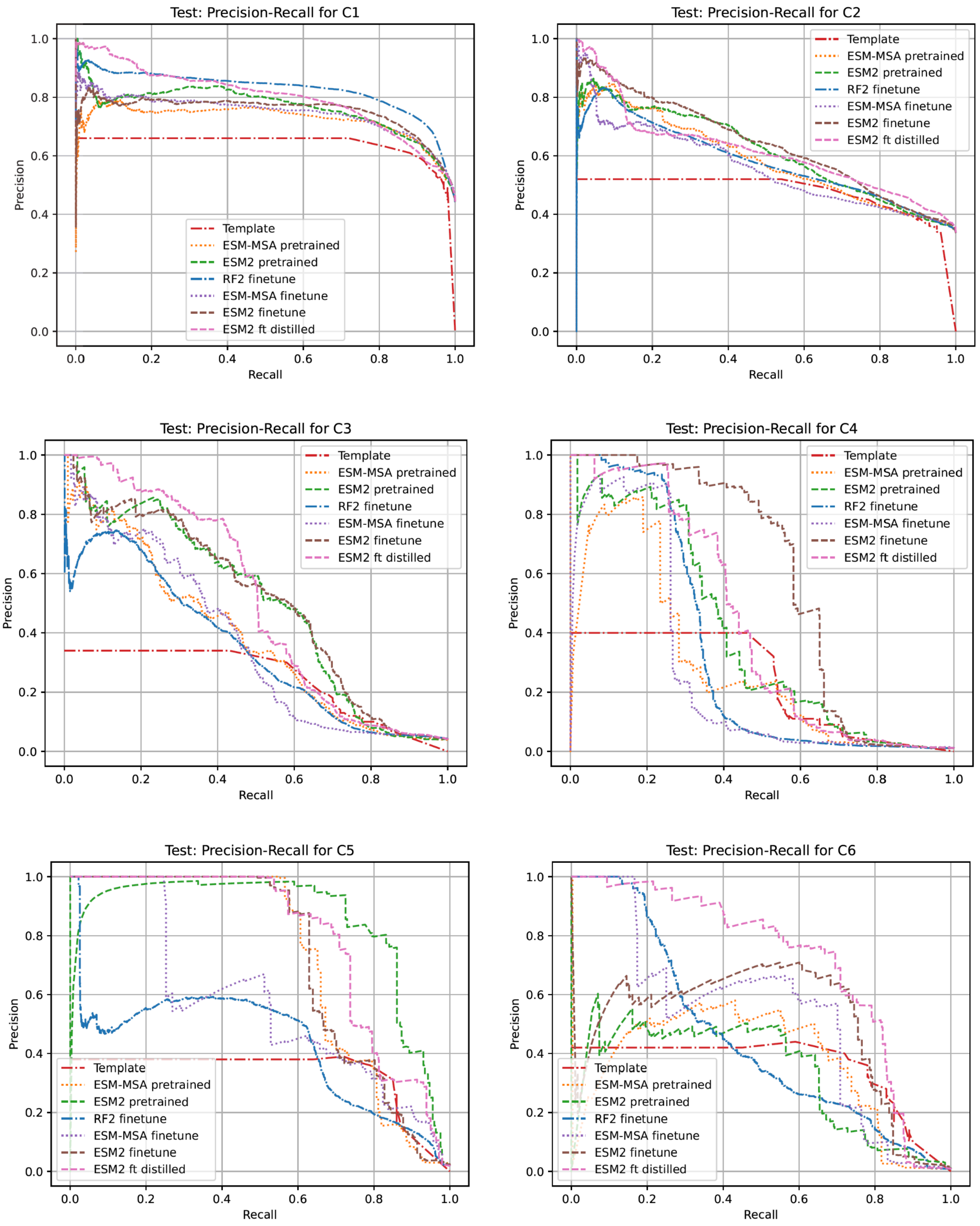

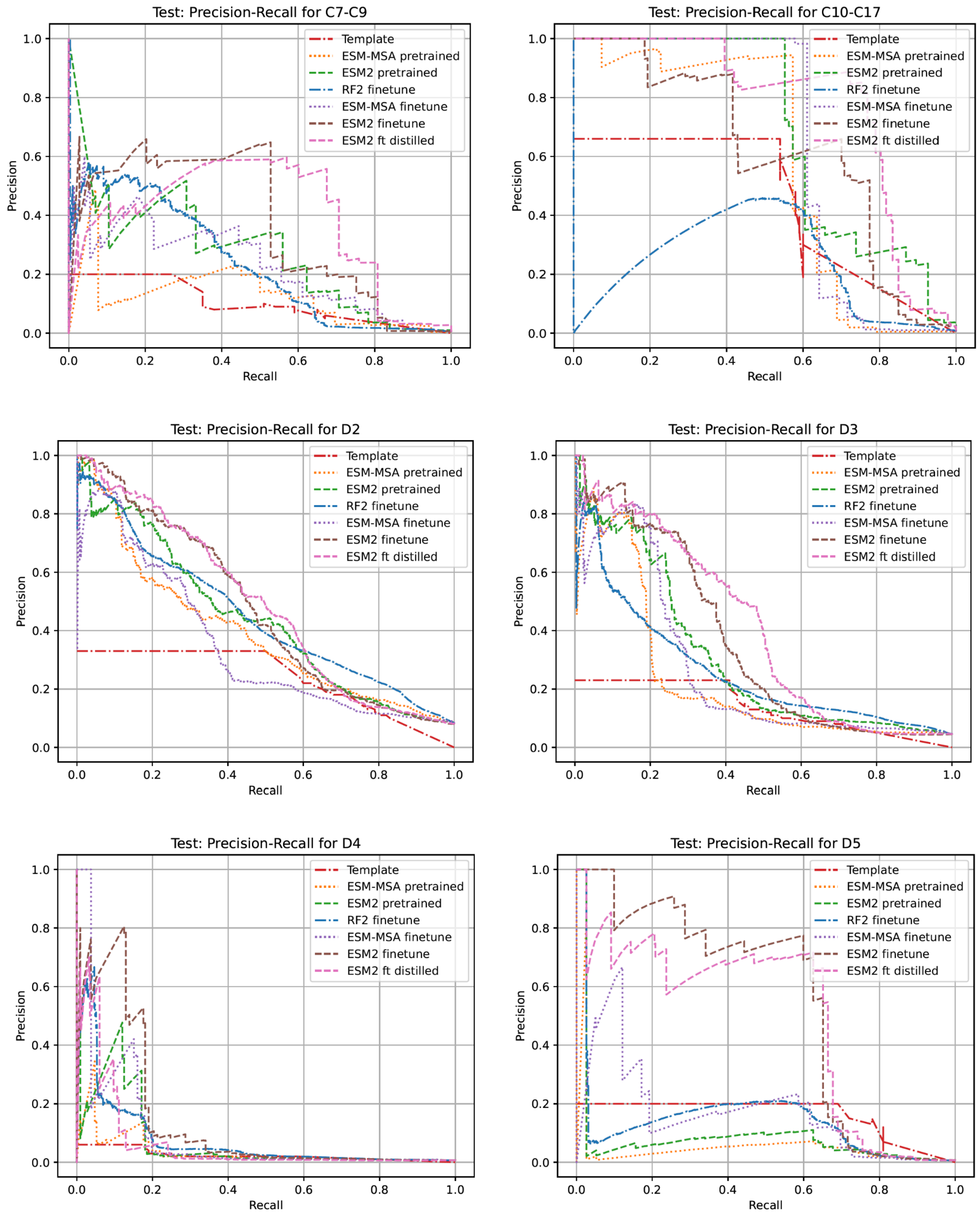

Test: Precision-Recall for C7-C9
Test: Precision-Recall for C10-C17
Test: Precision-Recall for D2
Test: Precision-Recall for D3
Test: Precision-Recall for D4
Test: Precision-Recall for D5
Precision
Recall
0.0
0.2
0.4
0.6
0.8
1.0
Template
ESM-MSA pretrained
ESM2 pretrained
RF2 finetune
ESM-MSA finetune
ESM2 finetune
ESM2 ft distilled

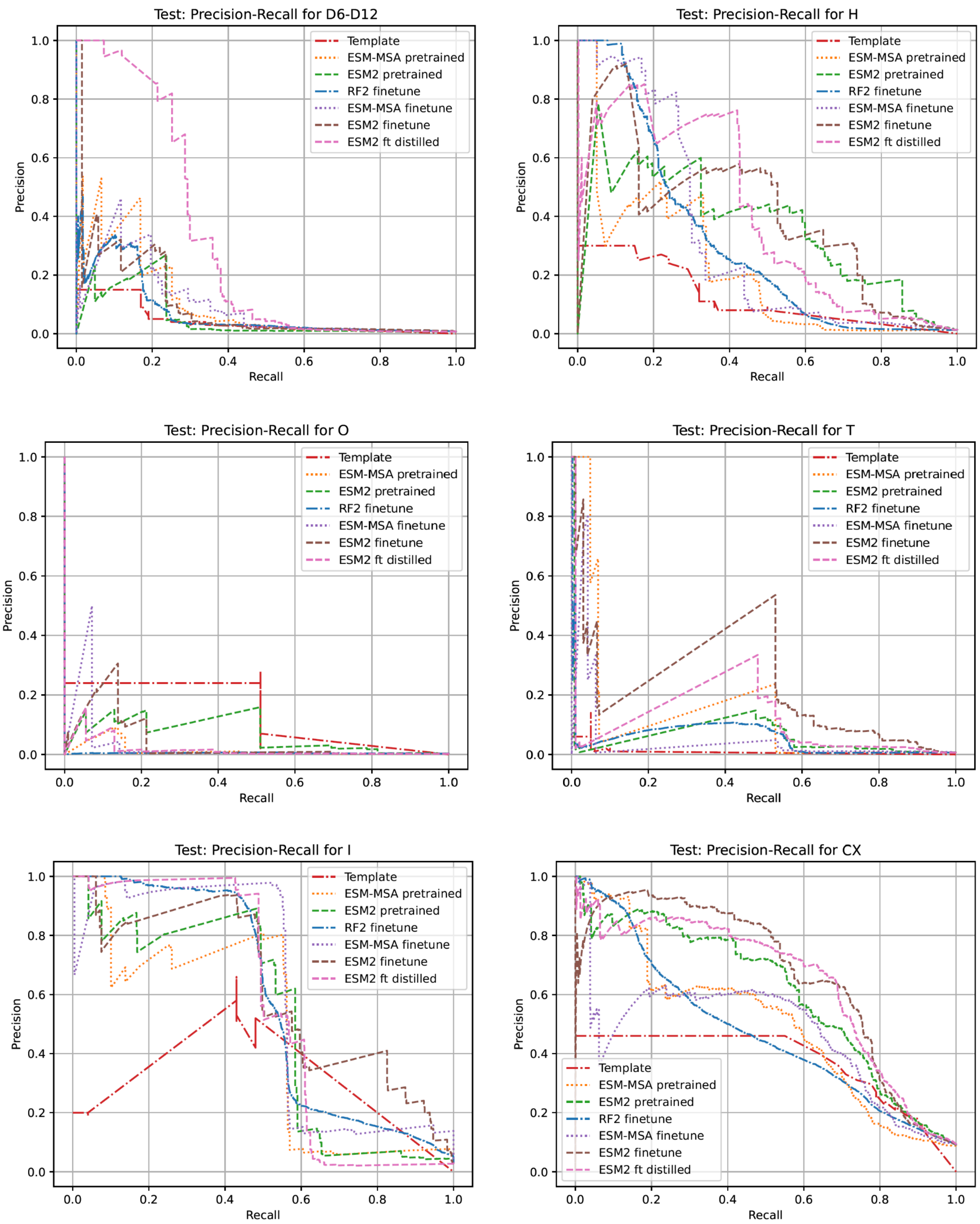
Test: Precision-Recall for D6-D12
Test: Precision-Recall for H
Test: Precision-Recall for O
Test: Precision-Recall for T
Test: Precision-Recall for I
Test: Precision-Recall for CX
Precision
Recall
0.0
0.2
0.4
0.6
0.8
1.0
Template
ESM-MSA pretrained
ESM2 pretrained
RF2 finetune
ESM-MSA finetune
ESM2 finetune
ESM2 ft distilled

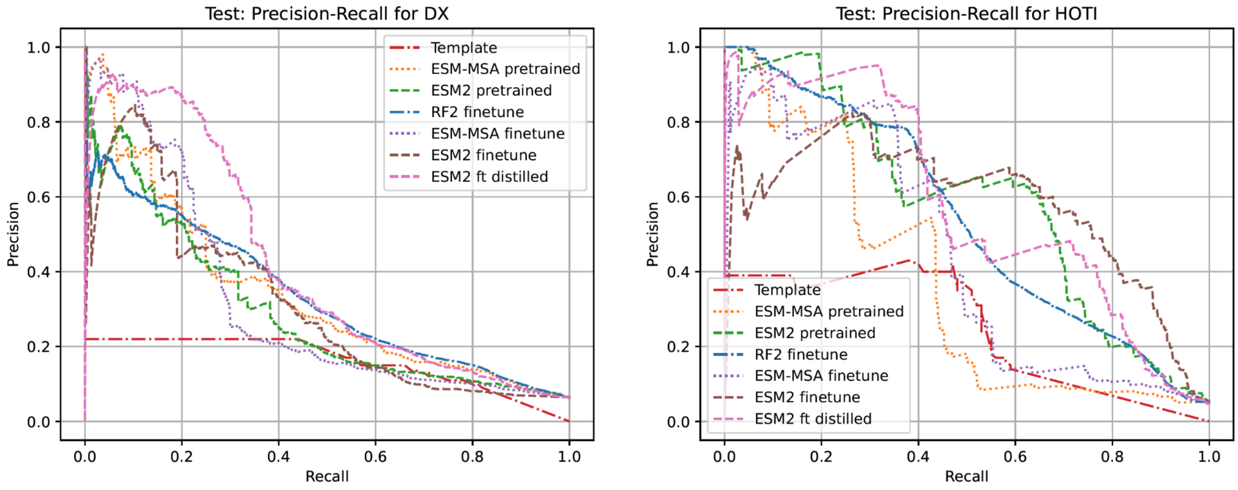

Supplementary Figure 4. Confusion matrices of various methods on test set proteins with a single homo-oligomer symmetry label. Singly labeled proteins comprise 90% of the test set with 60,582 structures.

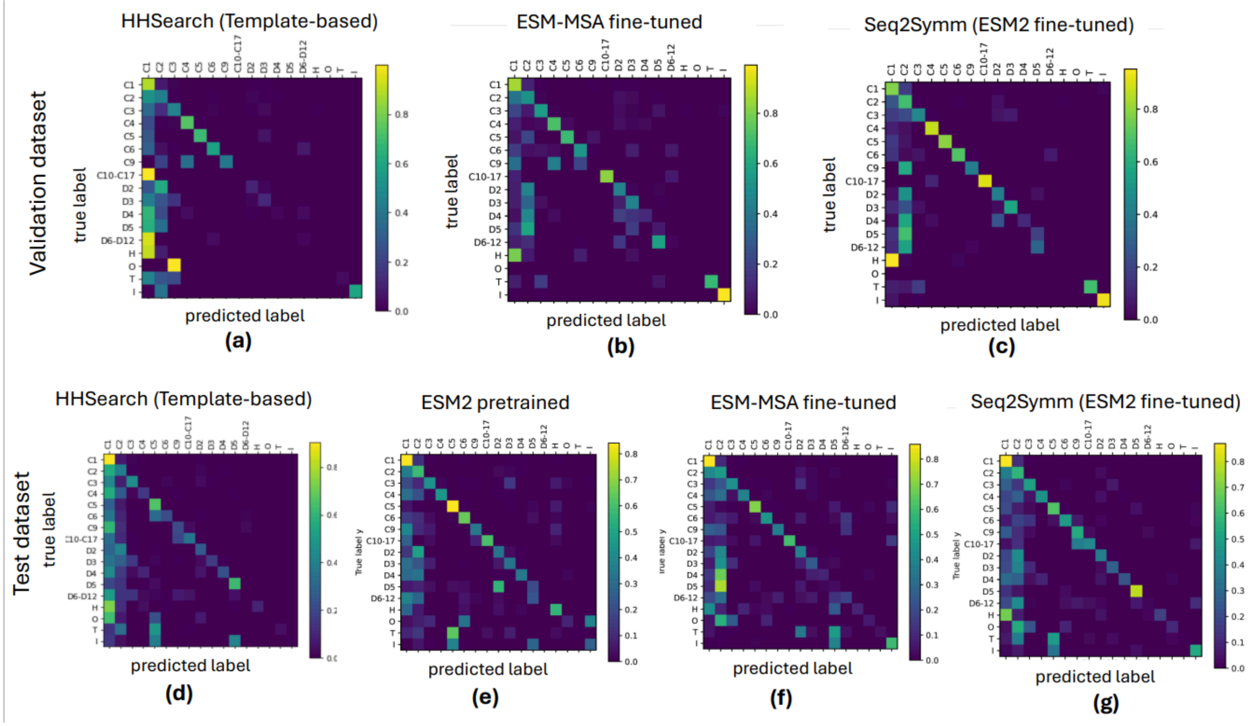

Supplementary Figure 5. Test AUC-PR, macro averaged across classes on the test split of the “no-homology” data splits. Here, all models are trained on the training split of the “no-homology” data split and the template-based method can only use hits from the training and validation splits to infer labels on the test data.

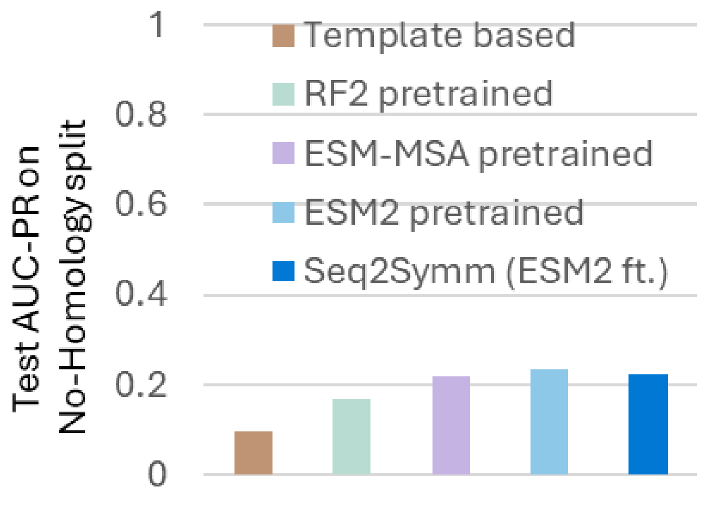

Supplementary Figure 6. Class-wise Test AUC-PR on the no-homology split

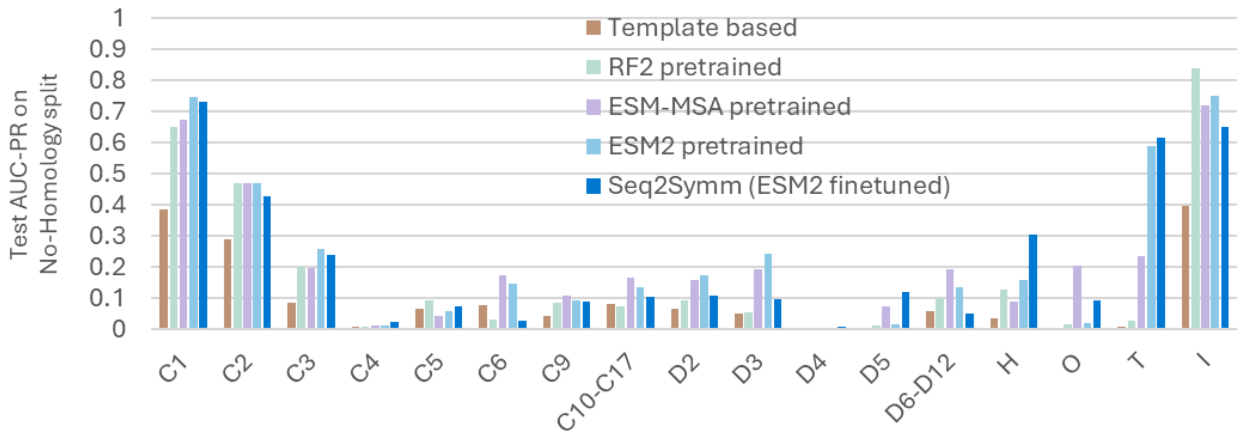

Supplementary Figure 7. AUC-PR of Seq2Symm on our *de novo* test set

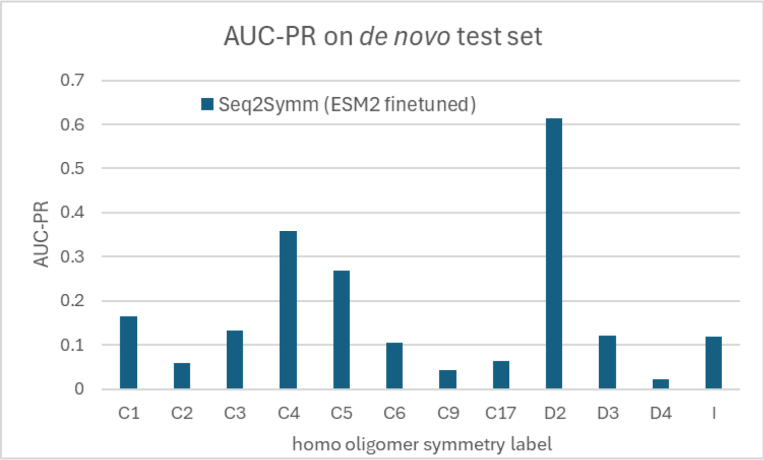

Supplementary Figure 8. We show the diversity of homo-oligomer symmetry within each protein family. **(a)** shows a histogram of the number of unique symmetry labels per protein family in the full dataset (PFam annotations are available for 66% of the structures). 50% of the protein families have more than one symmetry. While the remaining 50% of protein families have a single symmetry label, this data only constitutes 14.8% of all the protein structures from our dataset and 57% of these single-symmetry families have C1 symmetry i.e. they are monomers. **(b)** shows the entropy of the label counts within a protein family to give a sense of the label distribution within a family. A high entropy value implies that the symmetry labels are evenly distributed amongst all the unique symmetries observed for that protein family.

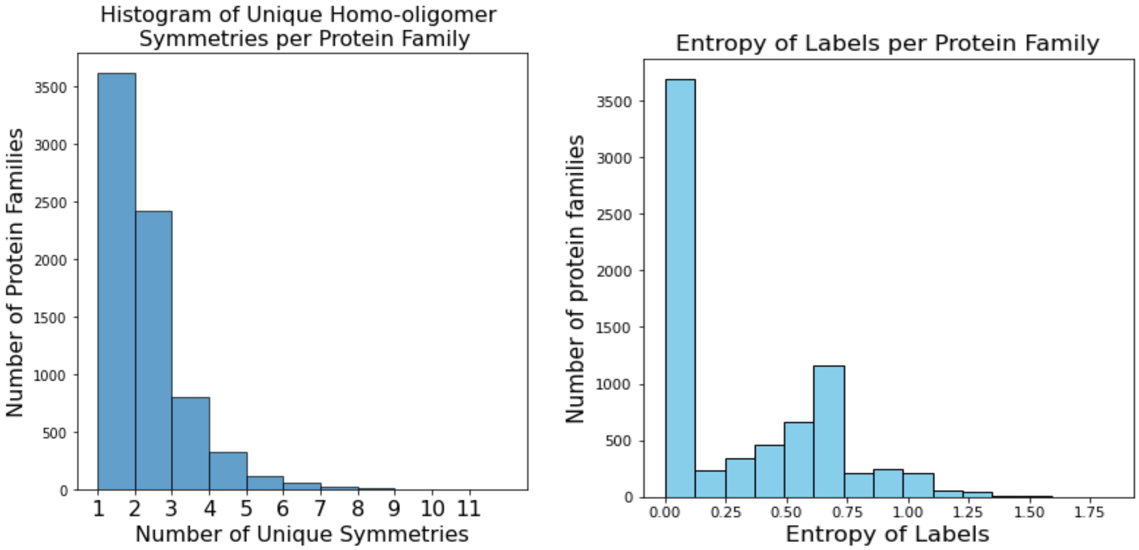

(a) (b)

Supplementary Figure 9. The plots below capture the protein family level performance of Seq2Symm by showing the distribution of the percentage recall and percentage precision over proteins from a family. **(a, b)** %Recall and %Precision respectively on test proteins from *seen* protein families **(c, d)** %Recall and %Precision respectively on test proteins from *unseen* protein families.

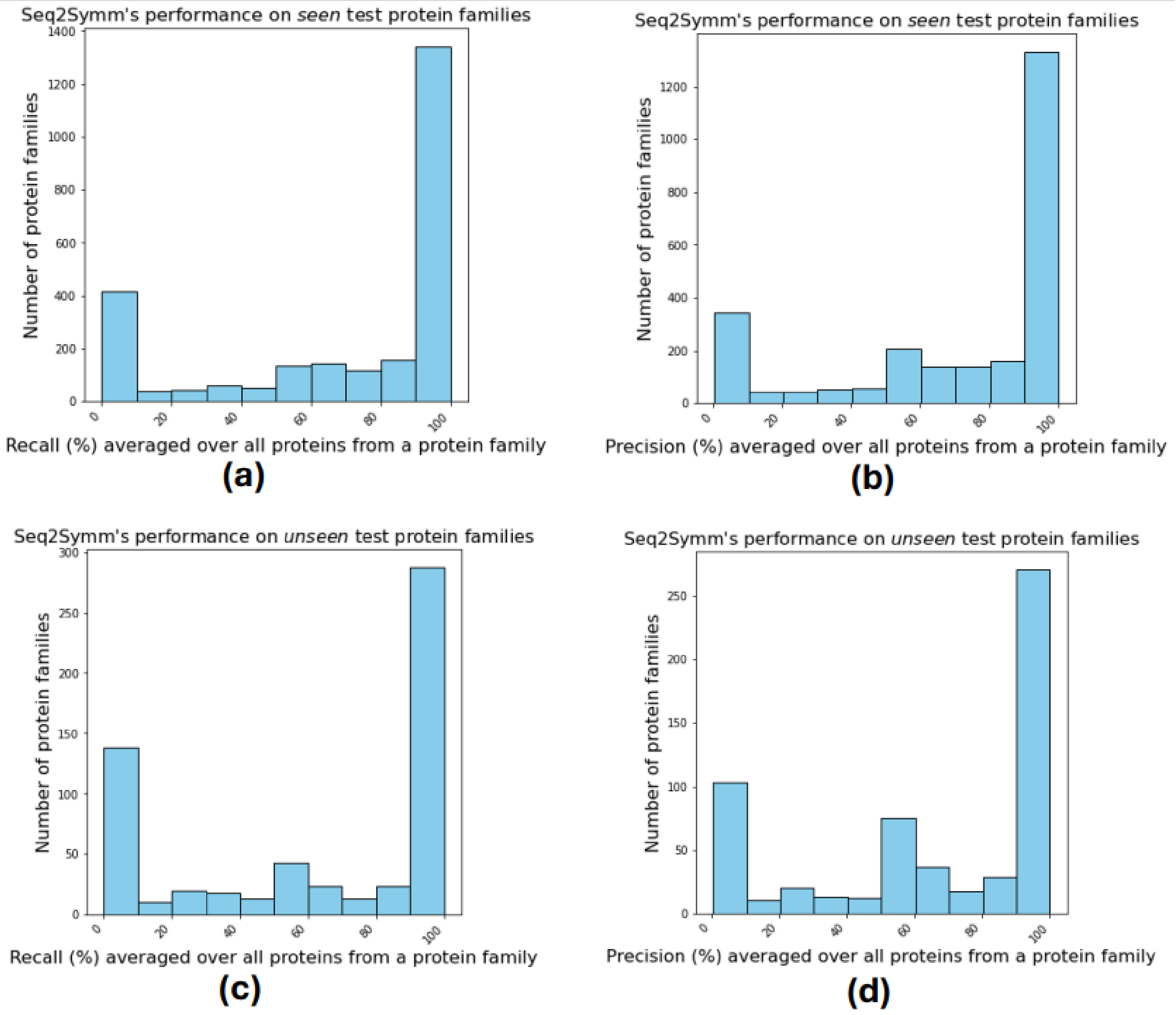

Supplementary Figure 10. (a) We look at Seq2Symm's predictions for each protein within an MSA for ~200 MSAs from the Test set. The number of unique oligomer symmetries are shown. **(c)** As anticipated, the number of unique oligomer symmetries is correlated with the number of protein sequences within an MSA. **(b)(d)** Analogous results are shown for 300 randomly selected MSAs from the train split.

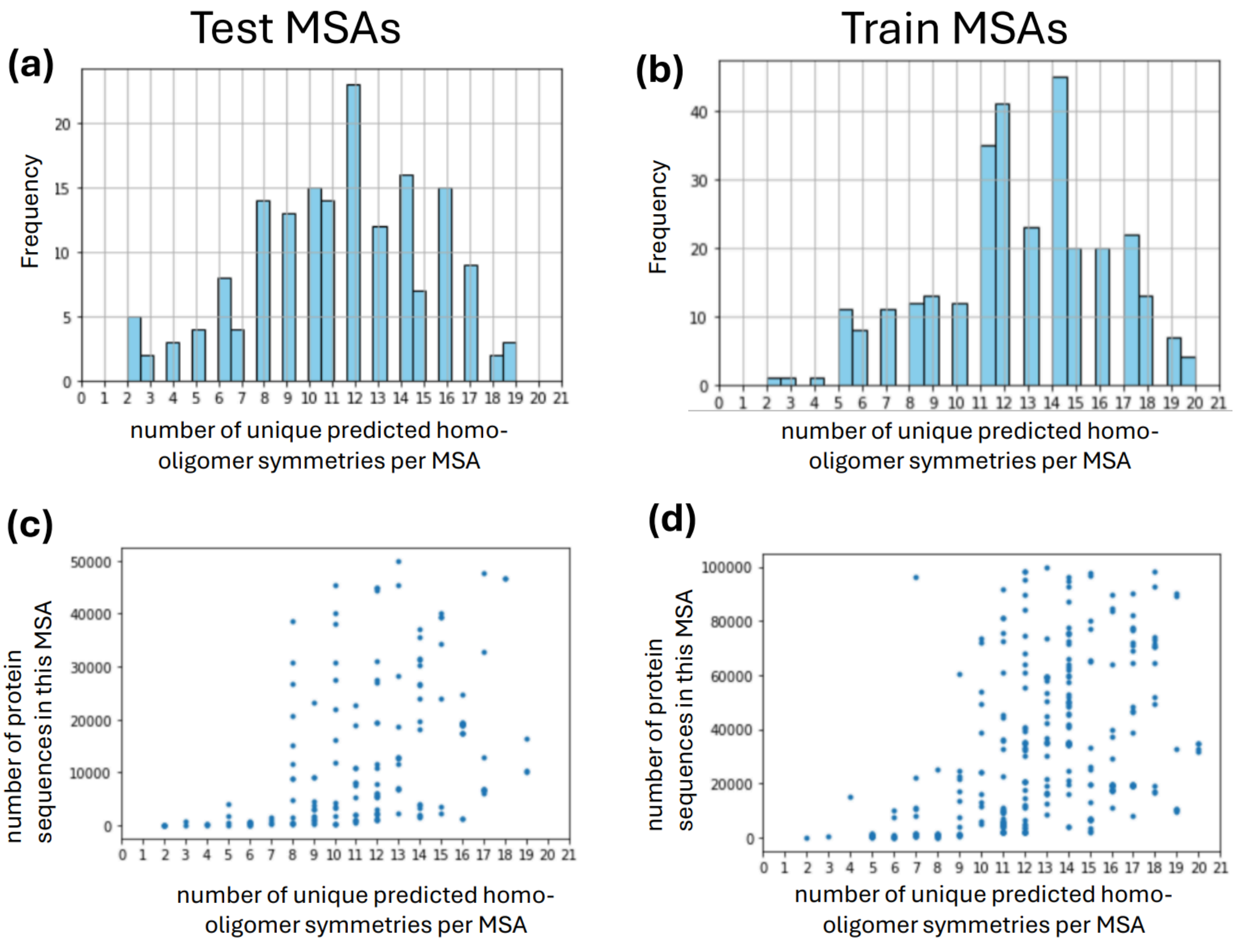

Supplementary Figure 11. Examples from our dataset showing the diversity of homo-oligomer symmetry labels within an MSA for four different MSAs. Cropped segments of the MSA, both in the length as well as number of sequences, are shown for brevity. We also show how a single protein sequence `A0A0C5GTQ` from metagenomic data is part of several MSAs, where each MSA has different homo-oligomer symmetry(s).

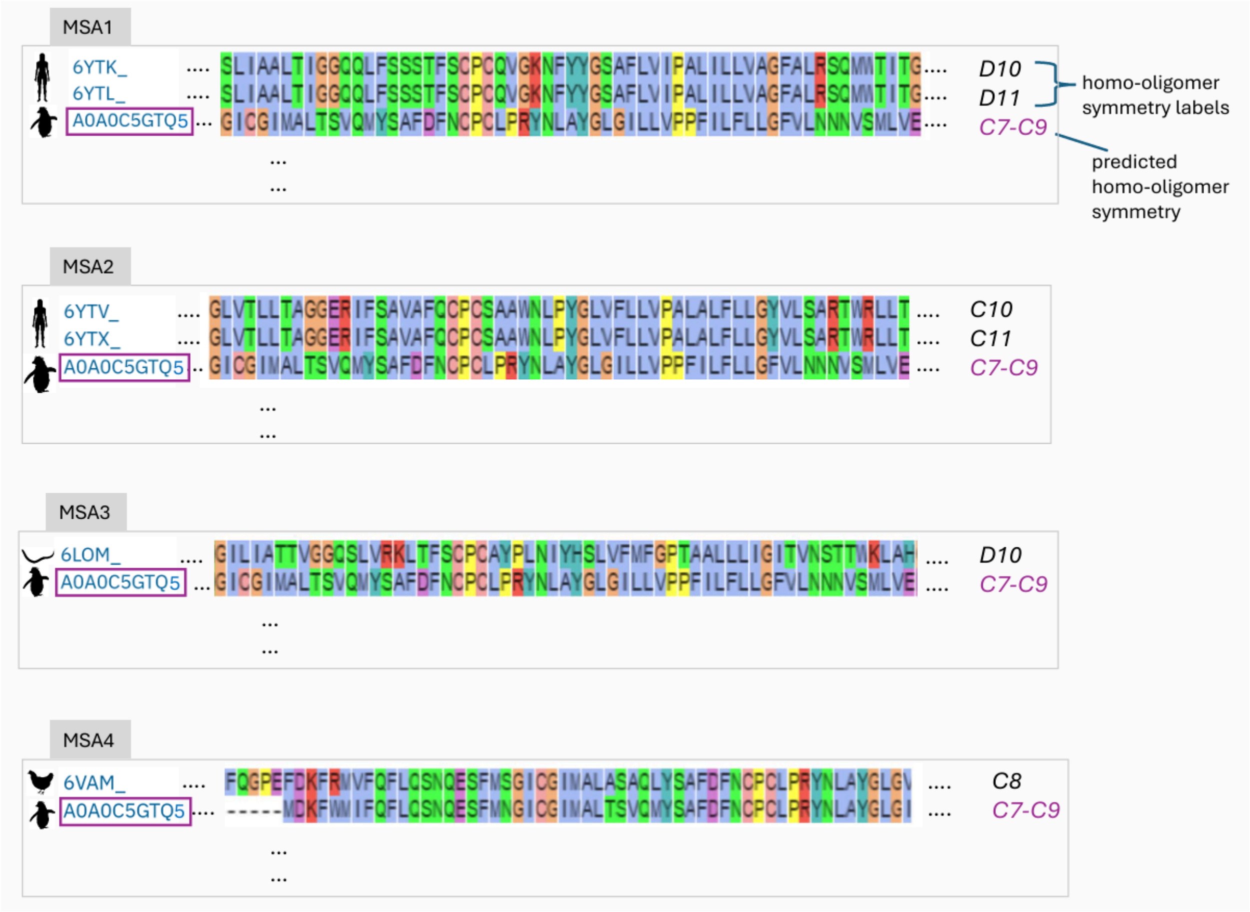

Supplementary Figure 12. Class-wise test AUC-PR in the "95% sequence identity" setting

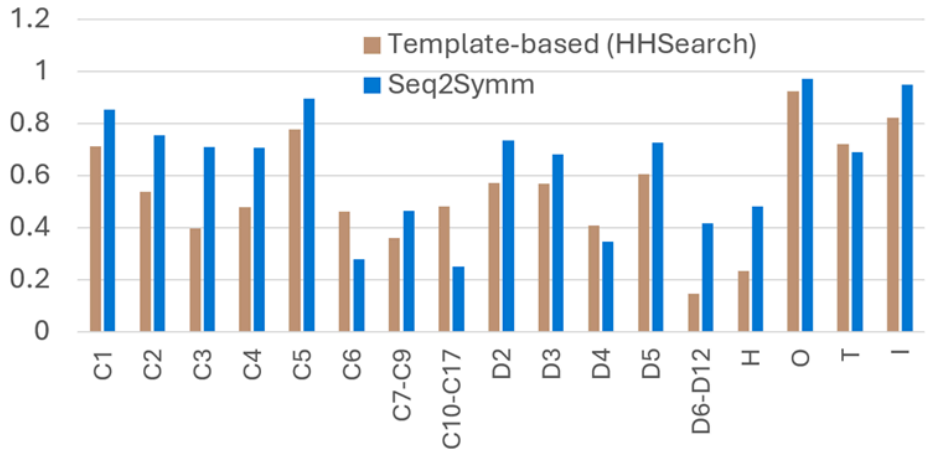

Supplementary Figure 13. Performance of the QUEEN model on our test dataset (a) before excluding proteins that overlap with the training data from QUEEN (b) after excluding proteins that overlap with the training. Once these “easy” examples have been excluded from the test set, the performance drops significantly for all classes except quaternary state 24. There are increases for either precision or recall, but not F1-score or AUPR for quaternary states 5 and 12. There is an anomalous increase in AUPR for quaternary state 24 and it happens since this is a very small class (only 30 examples left after filtering) and so we expect a higher variance in performance.

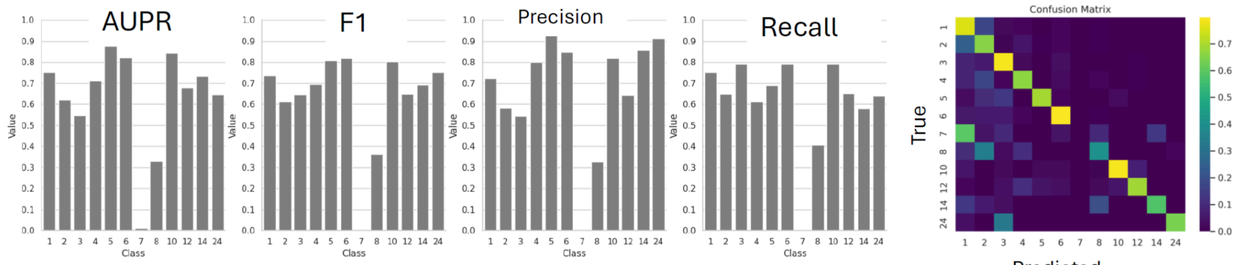

(a) QUEEN: Test set performance, ***before*** filtering out proteins from QUEEN’s training data

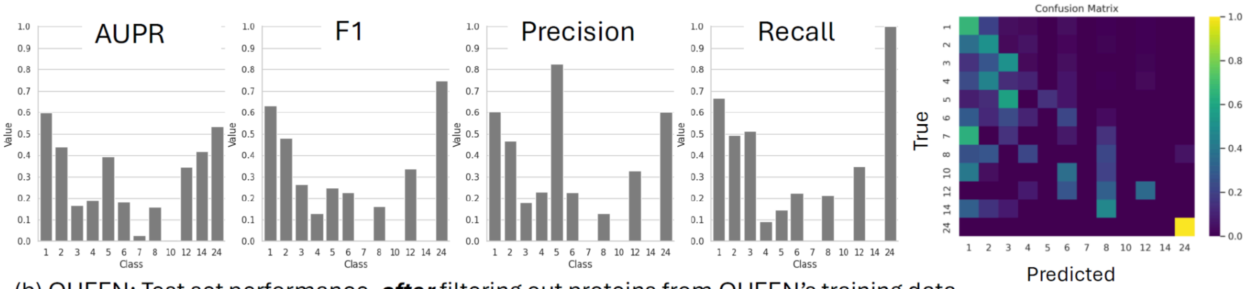

(b) QUEEN: Test set performance, ***after*** filtering out proteins from QUEEN’s training data

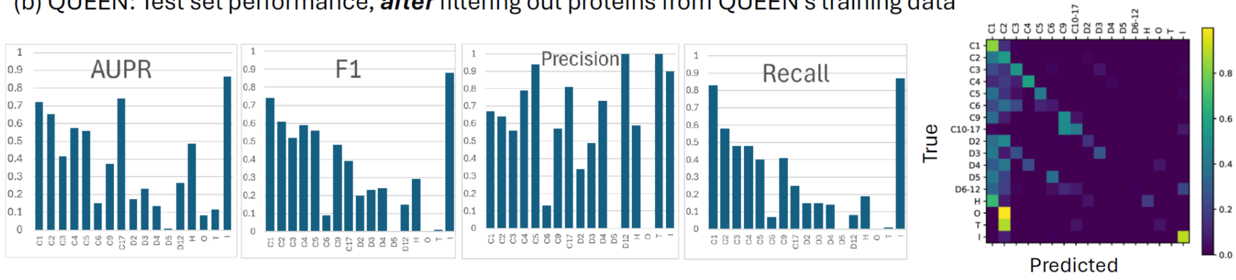

(c) Seq2Symm performance on the reduced test set, ***after*** filtering out proteins from QUEEN’s training data

Supplementary Figure 14 Prevalence of multiple homo-oligomeric symmetries per protein, in the five proteomes shown in main text Figure 4.

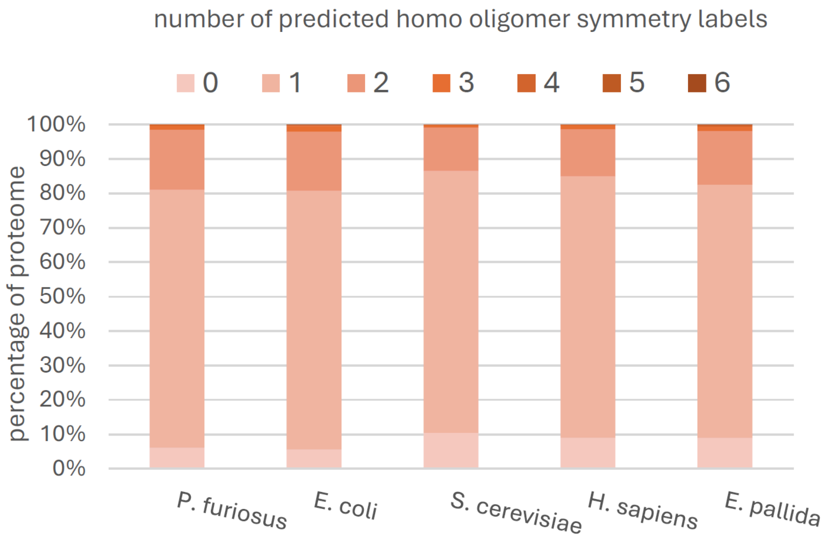

Supplementary Figure 15. (a) Predicted and true label distributions for a cluster of proteins from the validation set, where the model makes mistakes on the 'H' symmetry proteins. **(b)** Predicted and true label distributions for a cluster of proteins from the validation set, where the model overpredicts 'C2' symmetry over 'D2', whereas the true prevalence of these symmetries is the reverse.

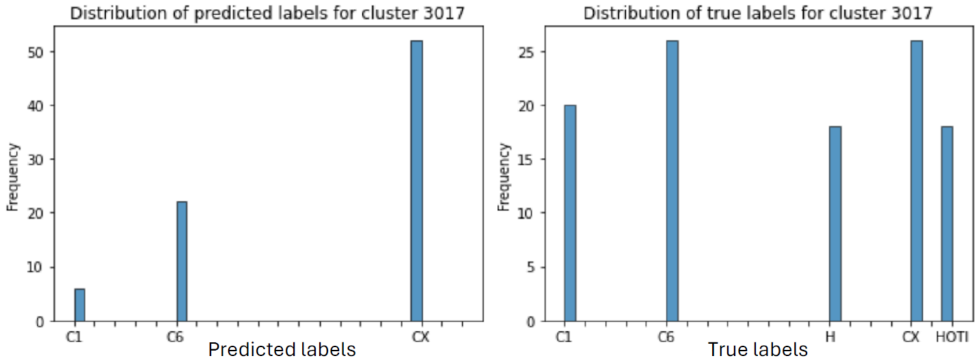

(a) Distribution of predicted and true labels for cluster 3017

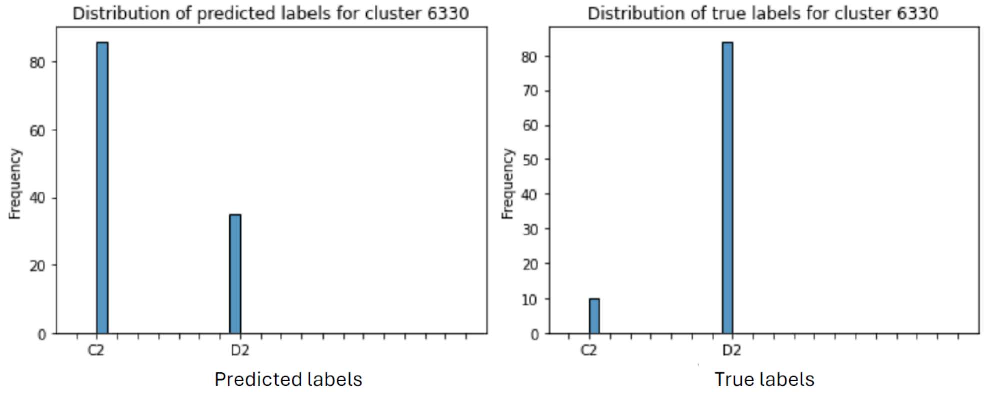

(b) Distribution of predicted and true labels for cluster 6330

Supplementary Figure 16. We show the errors made by Seq2Symm for each class across different bins of predicted probability (each bin is of size 0.1). Orange bars show false positive error % and blue bars show false negative error %.

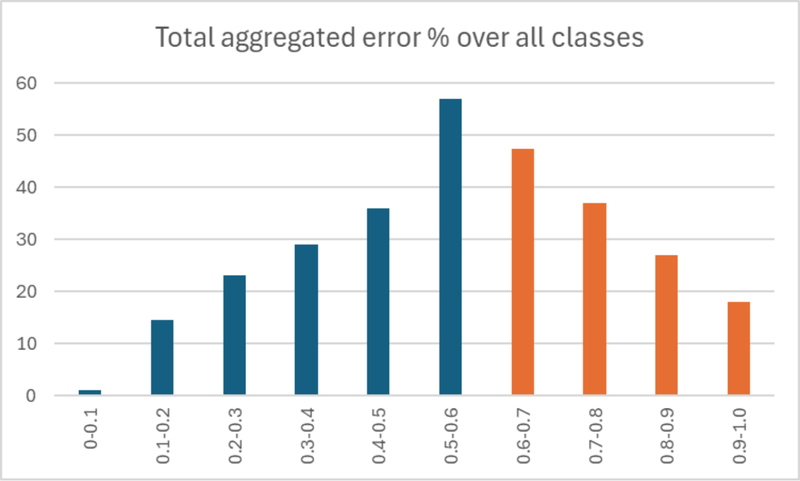

Supplementary Tables

Supplementary Table 1. Class-wise AUC-PR on the validation set

	AUC-PR on validation set							
Labels	Num of samples	Template-based (HHsearch)	ESM-MSA pretrained	ESM2 pretrained	RF2 finetuned	ESM-MSA finetuned	Seq2Symm (ESM2 finetuned)	Seq2Symm + distillation
C1	11107	0.593	0.734	0.783	0.831	0.735	0.725	0.746
C2	9488	0.444	0.628	0.618	0.560	0.566	0.604	0.659
C3	1406	0.320	0.413	0.516	0.547	0.525	0.585	0.557
C4	708	0.695	0.661	0.794	0.899	0.745	0.855	0.846
C5	95	0.394	0.535	0.793	0.651	0.631	0.793	0.793
C6	285	0.366	0.352	0.266	0.649	0.473	0.578	0.676
C7-C9	71	0.112	0.115	0.245	0.039	0.105	0.154	0.581
C10-C17	149	0.208	0.559	0.764	0.651	0.907	0.964	0.967
D2	3827	0.428	0.544	0.628	0.593	0.437	0.729	0.713
D3	1524	0.471	0.126	0.469	0.253	0.253	0.659	0.641
D4	326	0.062	0.108	0.171	0.272	0.219	0.188	0.318
D5	145	0.039	0.223	0.028	0.007	0.057	0.021	0.189
D6-D12	373	0.415	0.134	0.339	0.052	0.417	0.599	0.447
H	277	0.176	0.044	0.037	0.101	0.022	0.080	0.178
O	2	0.000	0.000	0.002	0.000	0.000	0.000	0.001
T	131	0.232	0.432	0.402	0.033	0.587	0.632	0.591
I	238	0.799	0.969	0.870	0.965	0.978	0.759	0.990
CX (C3 to C17)	2714	0.438	0.579	0.694	0.706	0.711	0.726	0.686
DX (D3-D12)	2368	0.330	0.293	0.557	0.376	0.320	0.675	0.597
HOTI	648	0.358	0.493	0.464	0.613	0.473	0.275	0.601

**Macro Average**		0.344	0.397	0.472	0.440	0.458	0.530	0.589
**Macro Avg excl. coarse grained classes**		0.338	0.387	0.454	0.418	0.450	0.525	0.582

Supplementary Table 2. Maximum F1-score achieved by the various methods on the validation set, obtained by varying the classifier threshold

	num of labels	Template - based HHsearch	ESM-MSA pretrained	ESM2 pre-trained	RF2 fine-tuned	ESM-MSA fine-tuned	Seq2 Symm (ESM 2 fine-tuned)	Seq2Symm + distillation
C1	11107	0.67	0.748	0.745	0.801	0.750	0.753	0.759
C2	9488	0.55	0.593	0.584	0.528	0.547	0.595	0.608
C3	1406	0.42	0.429	0.541	0.530	0.529	0.598	0.621
C4	708	0.69	0.716	0.803	0.862	0.759	0.880	0.882
C5	95	0.41	0.739	0.882	0.660	0.690	0.882	0.857
C6	285	0.45	0.532	0.321	0.558	0.549	0.641	0.686
C9	71	0.23	0.367	0.462	0.057	0.325	0.346	0.723
C17	149	0.54	0.654	0.776	0.571	0.951	0.951	0.951
D2	3827	0.51	0.581	0.633	0.617	0.439	0.703	0.656
D3	1524	0.48	0.160	0.512	0.339	0.378	0.717	0.697
D4	326	0.13	0.177	0.259	0.232	0.271	0.272	0.387
D5	145	0.06	0.343	0.138	0.027	0.214	0.082	0.294
D12	373	0.59	0.199	0.524	0.118	0.619	0.779	0.674
H	277	0.44	0.145	0.123	0.156	0.121	0.180	0.292
O	2	0.00	0.000	0.007	0.000	0.001	0.001	0.001
T	131	0.40	0.524	0.524	0.484	0.657	0.742	0.728

I	238	0.76	0.971	0.847	0.932	0.971	0.689	0.879
CX	2714	0.53	0.644	0.665	0.618	0.700	0.705	0.737
DX	2368	0.45	0.370	0.563	0.434	0.488	0.669	0.652
HOTI	648	0.53	0.578	0.533	0.543	0.508	0.478	0.590
**Macro Avg.**		0.44	0.474	0.522	0.453	0.523	0.583	0.634
**Macro Avg. excl. coarse grained classes**		0.43	0.463	0.511	0.440	0.516	0.577	0.629

F1-score of all methods on the held-out test dataset

We use the per-class thresholds that give the maximum F1-score on the validation dataset (See Supplementary Table 2 above) to compute precision, recall and F1-score on the unseen test dataset. The resulting F1-score is shown in Supplementary Table 3.

Supplementary Table 3. F1-score on the held-out test dataset, using the “best threshold” to classify, computed on the validation dataset

**Test data: F1-score based on thresholds selected using the validation dataset**								
	num of labels	Template-based (HHsearch)	ESM-MSA pretrained	ESM2 pretrained	RF2 finetuned	ESM-MSA finetuned	Seq2Symm (ESM2 finetuned)	Seq2Symm + distillation
C1	28724	0.71	0.76	0.75	0.80	0.76	0.77	0.75
C2	21875	0.56	0.57	0.58	0.59	0.55	0.58	0.61
C3	2670	0.32	0.42	0.51	0.38	0.37	0.54	0.55
C4	717	0.37	0.31	0.43	0.41	0.38	0.6	0.47
C5	1220	0.46	0.71	0.77	0.31	0.50	0.59	0.74
C6	584	0.51	0.30	0.30	0.42	0.62	0.66	0.66
C9	286	0.18	0.26	0.36	0.19	0.38	0.58	0.56
C17	382	0.53	0.40	0.45	0.49	0.67	0.44	0.52

D2	5176	0.35	0.40	0.47	0.45	0.38	0.45	0.46
D3	2893	0.27	0.21	0.32	0.28	0.16	0.41	0.42
D4	432	0.06	0.06	0.17	0.15	0.10	0.25	0.11
D5	384	0.21	0.02	0.04	0.07	0.24	0.33	0.05
D12	409	0.15	0.08	0.11	0.09	0.08	0.16	0.19
H	813	0.24	0.34	0.42	0.34	0.24	0.49	0.42
O	108	0.30	0.00	0.12	0.00	0.00	0.07	0.01
T	392	0.05	0.10	0.01	0.03	0.08	0.06	0.02
I	1744	0.52	0.38	0.08	0.55	0.62	0.59	0.59
CX	5859	0.44	0.54	0.58	0.46	0.35	0.61	0.65
DX	4118	0.27	0.32	0.30	0.36	0.23	0.34	0.41
HOTI	3385	0.41	0.39	0.42	0.46	0.49	0.45	0.51
**Macro Average**		0.36	0.33	0.36	0.34	0.36	0.45	0.44
**Macro Avg. excl. coarse-grained classes**		0.34	0.31	0.35	0.33	0.36	0.45	0.42

Supplementary Table 4. Statistics of the various homo-oligomer label combinations in our dataset, where the labels have been sorted by the number of examples in our dataset, with single labels highlighted for easier perusal.

**C1**	108210
**C2**	83812
**D2**	25861
**C3**	11582
**I**	9412
**D3**	8510
C1 C2	8315
**C5**	5113
**O**	3988
**C4**	3405
**H**	3382
C2 D2	2937
**T**	2889
**D5**	2813
**C6**	2547
**D4**	2246
**C7**	1303
**D6**	1055
**D7**	1051
**C11**	792
C1 C3	713
C1 D2	700
C2 D3	659
C3 D3	575

C1 H	89
**D11**	88
C1 C4	87
**C16**	86
C1 C2 D3	80
**D10**	80
C3 T	79
C1 O	73
C1 T	71
C33	66
C2 C3 D3	63
C2 C4	61
C1 C2 C3 I	60
D17	53
D9	50
C2 I	50
C1 C7	49
C1 C3 D3	44
C22	44
D39	41
D16	40
C17	40
C1 C6	39
C1 C2 C3	35

C1 C2 T	17
C2 C5	15
C3 C6	14
C1 C3 T	13
C1 C2 C3 D3	13
C2 D2 D6	12
C1 D6	12
C3 D2	12
C2 D2 D4	11
C1 C2 D5	10
C2 D2 D3	10
C1 C4 D4	9
C1 C2 D6	9
C9 D9	9
C2 D6	8
C1 D8	8
C1 C2 D4	7
C3 D3 T	7
C2 C3 D3 O	6
D2 D4	6
C18	6
C1 C2 C4	5
C2 C3 T	4
C2 D2 T	4

C12	401
C2 D4	397
D8	385
C8	378
C1 C2 D2	312
C9	300
C10	289
C1 D3	271
C5 D5	265
C4 D4	233
C15	206
C2 C3	195
C7 D7	189
C1 D4	155
C14	146
C2 D5	135
C13	105
C2 H	102
C34	102
C6 D6	99

C1 D5	35
C2 T	35
C32	32
C30	30
D3 O	29
C1 D7	28
C2 C4 D4	26
C26	26
C1 C5	25
C12 D12	24
C1 C8	24
C4 O	24
D12	24
C8 D8	22
C21	21
C5 I	20
C5 D3	20
D2 D5	20
C2 O	19
C2 C6	18

C2 D8	4
C4 D2	4
C1 C2 C4 D4	4
C1 C9	3
C12 O	3
C1 C2 H	3
C2 D3 O	2
C2 D2 O	2
C1 C2 D3 T	2
C2 C4 D2	2
C1 D3 O	1
C1 C3 C6	1
O T	1
C3 D3 O	1
C39	1
C27	1
C3 O	1
D2 D6	1
C2 C3 D3 T	1

Supplementary Table 5. No-homology data split (described in the text above): Statistics of data splits generated using a sequence similarity e-value cut-off of 0.1.

	train	validation	test
C1	84421	8815	11114
C2	67111	10232	8395
C3	9270	937	1421
C4	2867	194	171
C5	3360	475	1271
C6	1456	662	430
C9	879	671	429
C17	376	601	1101
D2	23705	1353	1890
D3	7755	601	919
D4	2250	142	90
D5	1793	791	455
D12	1710	191	934
H	2081	774	587
O	3426	129	538
T	2173	198	562
I	2980	4213	2207

Supplementary Table 6a. Seq2Symm's performance (both with and without distillation), stratified by whether the test protein has a Pfam annotation seen during training or not. We show class-wise test AUC-PR for the set of proteins with seen Pfam annotations on the left side of the table and for the set of proteins with unseen protein family annotations on the right side of the table.

	Pfam **seen** subset (2476 seen protein families)			Pfam **unseen** subset (589 unseen protein families)		
	No. of proteins	Seq2 Symm	Seq2 symm + distill	No. of proteins	Seq2 Symm	Seq2symm + distill
C1	26911	0.76	0.81	1813	0.56	0.61
C2	20013	0.66	0.63	1862	0.46	0.43
C3	2361	0.50	0.53	309	0.52	0.42
C4	703	0.60	0.46	14	0.02	0.01
C5	945	0.83	0.87	275	0.39	0.59
C6	553	0.53	0.75	31	0.01	0.02
C9	262	0.41	0.45	24	0.10	0.27
C10-C17	531	0.70	0.86	120	0.44	0.39
D2	4584	0.52	0.53	592	0.14	0.17
D3	2325	0.43	0.47	568	0.08	0.19
D4	382	0.17	0.08	50	0.02	0.01
D5	364	0.58	0.52	20	0.01	0.01
D6-D12	391	0.12	0.36	77	0.04	0.02
H	591	0.44	0.51	222	0.45	0.16
O	108	0.04	0.02	0	0.00	0.00
T	273	0.44	0.26	119	0.09	0.05
I	1706	0.65	0.56	38	0.46	0.39

Supplementary Table 6b. Seq2Symm's performance (both with and without distillation), stratified by whether the test protein has a CATH superfamily seen during training or not. We show class-wise AUC-PR for the set of proteins with seen CATH annotations on the left side of the table and for the set of proteins with the unseen CATH superfamilies on the right side of the table.

	CATH superfamily **seen** subset (total 1289 seen)			CATH superfamily **unseen** subset (total 491 unseen)		
	No. of proteins	Seq2 Symm	Seq2symm + distill	No. of proteins	Seq2 Symm	Seq2symm + distill
C1	27647	0.76	0.81	1077	0.54	0.62
C2	20879	0.66	0.63	996	0.36	0.37
C3	2449	0.52	0.53	221	0.35	0.24
C4	661	0.63	0.47	56	0.02	0.09
C5	1005	0.79	0.82	215	0.55	0.81
C6	557	0.53	0.74	27	0.03	0.08
C9	253	0.42	0.48	33	0.04	0.04
C10-C17	651	0.66	0.79	0	0.00	0.00
D2	4724	0.49	0.51	452	0.34	0.30
D3	2337	0.44	0.49	556	0.10	0.13
D4	400	0.16	0.08	32	0.03	0.01
D5	339	0.62	0.56	45	0.03	0.01
D6-D12	426	0.11	0.28	42	0.19	0.58
H	813	0.43	0.41	0	0.00	0.00
O	92	0.05	0.03	16	0.00	0.00
T	247	0.48	0.29	145	0.16	0.08
I	1738	0.66	0.57	6	0.01	0.00

Supplementary Table 7. Statistics of the Unifold test set

Class	Counts
C1	9
C2	60
C3	7
C4	1
D2	7
H	1

Confusion matrix of Seq2Symm predictions on the Unifold test set

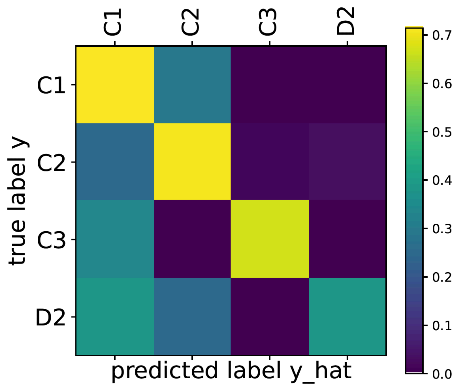

Supplementary Table 8. QUEEN performance showing various metrics on the Validation Set

(a) Before filtering for sequence-similar proteins with QUEEN's training data.

Class	No. of samples	AUPR	F1	Precision	Recall
1	10196	0.778729	0.72978	0.730354	0.729208
2	8229	0.606769	0.592664	0.557723	0.632276
3	2546	0.708442	0.629968	0.600071	0.663001
4	9881	0.784449	0.761101	0.880048	0.670479
5	475	0.771125	0.718016	0.945017	0.578947
6	5482	0.855001	0.828346	0.813908	0.843305
7	0	0	0	0	0
8	1350	0.853785	0.711322	0.592885	0.888889
10	675	0.463564	0.406032	0.935829	0.259259
12	1950	0.898241	0.918747	0.952643	0.887179
14	70	0.003458	0	0	0
24	5	0.00139	0	0	0

(b) After filtering for sequence-similar proteins with QUEEN's training data.

Class	No. of samples	AUPR	F1	Precision	Recall
1	3934	0.74621	0.692318	0.705945	0.679207
2	2939	0.488323	0.470228	0.411705	0.548146
3	510	0.38704	0.301676	0.287234	0.317647
4	1393	0.180037	0.184211	0.312178	0.130653
5	75	0.03539	0	0	0
6	415	0.05576	0.006795	0.00641	0.007229
7	0	0	0	0	0

8	45	0.008711	0	0	0
10	400	0.346344	0	0	0
12	160	0.035545	0	0	0
14	70	0.019932	0	0	0
24	0	0	0	0	0

Supplementary Table 9. QUEEN performance showing various metrics on the Test Set

(a) Before filtering for sequence-similar proteins from QUEEN's training data.

Class	No. of samples	AUPR	F1	Precision	Recall
1	25990	0.752065	0.736382	0.722019	0.751327
2	18678	0.621343	0.613754	0.582832	0.648142
3	4276	0.545904	0.643955	0.54393	0.789055
4	11452	0.713551	0.694947	0.801003	0.613692
5	5800	0.875434	0.808162	0.975848	0.689655
6	9982	0.821549	0.818394	0.848071	0.790723
7	775	0.010987	0	0	0
8	1305	0.33042	0.362146	0.326757	0.40613
10	2025	0.8415	0.804424	0.819252	0.790123
12	1000	0.679196	0.646445	0.642928	0.65
14	1085	0.734068	0.692308	0.857143	0.580645
24	430	0.64454	0.752394	0.913621	0.639535

(b) After filtering for sequence-similar proteins from QUEEN's training data.

Class	No. of samples	AUPR	F1	Precision	Recall
1	9075	0.598813	0.6324	0.602293	0.665675
2	6506	0.440828	0.48124	0.469257	0.493852
3	836	0.166755	0.266418	0.180059	0.511962
4	2523	0.190438	0.131222	0.229023	0.091954
5	1200	0.392637	0.247875	0.825472	0.145833
6	1199	0.181625	0.225684	0.227891	0.22352
7	490	0.026258	0	0	0
8	375	0.158518	0.161453	0.12987	0.213333
10	125	0.004897	0	0	0
12	200	0.345506	0.338983	0.328638	0.35
14	455	0.418858	0	0	0
24	30	0.533333	0.75	0.6	1

Supplementary Table 10. Statistics of the five proteomes discussed in Fig 4 in the main text.

	Proteome	Size
E. coli	UP000000625	4,400
S. cerevisiae	UP000002311	6,060
H. sapiens	UP000005640	20,596
P. furiosus	UP000001013	2,044
E. pallida	aiptasia@reefgenomics: sea anemone model for coral biology	26,042

Supplementary Table 11a. Seq2Symm's performance (both with and without distillation), stratified by protein structural class. We obtain these structural classes from CATH annotations, which are available for 75% of our test dataset. We show results for the following categories: Mainly Alpha, Mainly Beta, Alpha-Beta, Other (the total number of structures from each category are indicated in brackets and the class-wise distribution is shown in the column under each category). This table shows the first two (the others are shown in Suppl. Table 11b). Rows where the models' performance for a structural class is higher than that seen for the other structural classes are highlighted.

	**Mainly Alpha (12230)**	Seq2Symm	Seq2Symm + distill.	**Mainly Beta (8040)**	Seq2Symm	Seq2Symm + distill.
C1	6927	0.812	0.903	4158	0.713	0.739
C2	4380	**0.677**	**0.718**	2258	0.471	0.526
C3	312	0.359	0.428	634	**0.713**	**0.677**
C4	113	0.347	0.297	59	**0.756**	**0.780**
C5	70	**0.882**	**0.928**	635	**0.921**	**0.976**
C6	61	0.141	0.302	204	**0.872**	**0.937**
C9	8	0.047	0.092	56	0.510	0.422
C10-C17	180	**1.000**	**1.000**	0	0.000	0.000
D2	584	0.468	0.581	384	0.319	0.532
D3	312	0.266	0.401	179	0.100	0.235
D4	77	0.026	0.254	26	0.158	0.052
D5	5	0.033	0.278	10	0.005	0.048
D6-D12	23	0.008	0.787	21	0.003	0.669
H	48	0.109	0.258	19	**0.483**	**0.685**
O	16	0.001	0.001	6	0.001	0.001
T	30	**0.900**	**0.606**	2	0.000	0.000
I	0	0.000	0.000	70	**0.842**	**0.994**

Supplementary Table 11b. Seq2Symm's performance (both with and without distillation), stratified by protein structural class. We obtain these structural classes from CATH annotations, which are available for 75% of our test dataset. We show results for the following categories: Alpha-Beta, Other (the total number of structures in each category are indicated in the brackets and the class-wise distribution is shown in the column under each category). Rows where the models' performance for a structural class is higher than that seen for the other structural classes are highlighted.

	**Alpha - Beta (9627)**	Seq2Symm	Seq2Symm + distill.	**Other (16550)**	Seq2Symm	Seq2Symm + distill.
C1	3215	0.783	0.737	6455	0.738	0.698
C2	3217	0.534	0.590	7182	**0.730**	**0.659**
C3	595	0.636	0.655	477	0.322	0.166
C4	89	0.189	0.027	239	0.600	0.293
C5	40	0.113	0.126	115	0.230	0.331
C6	207	**0.778**	**0.819**	40	0.488	0.494
C9	35	0.552	0.628	57	0.161	0.162
C10-C17	0	0.000	0.000	20	0.057	0.186
D2	1594	**0.664**	**0.618**	1510	0.366	0.334
D3	1113	0.294	0.409	929	**0.616**	**0.618**
D4	127	0.357	0.062	164	0.183	0.070
D5	10	0.164	0.094	230	**0.535**	**0.502**
D6-D12	118	0.128	0.086	88	0.023	0.019
H	8	0.001	0.001	46	0.164	0.057
O	28	0.013	0.094	43	0.101	0.006
T	5	0.003	0.001	149	0.053	0.022
I	5	0.001	0.001	272	0.428	0.286

Supplementary Table 12. Seq2Symm's performance (both with and without distillation), stratified by transmembrane proteins. We show class-wise AUC-PR for transmembrane proteins (left side of the table) and non-transmembrane proteins (right side of the table). Symmetry classes where the performance of the models is higher on the transmembrane set of proteins is highlighted in bold.

	Transmembrane			Non-Transmembrane		
	Size	Seq2Symm	Seq2Symm + distillation	Size	Seq2Symm	Seq2Symm + distillation
C1	4151	0.783	0.881	24573	0.749	0.779
C2	2413	0.570	0.634	19462	0.648	0.616
C3	479	0.423	0.345	2191	0.525	0.541
C4	236	**0.919**	**0.873**	481	0.403	0.185
C5	235	0.798	0.742	985	0.713	0.792
C6	23	0.004	0.018	561	0.526	0.739
C9	56	**0.485**	**0.538**	230	0.392	0.367
C10-C17	448	**0.880**	**0.921**	203	0.364	0.397
D2	133	**0.683**	**0.567**	5043	0.473	0.489
D3	57	0.009	0.013	2836	0.375	0.419
D4	90	0.497	0.442	342	0.069	0.027
D5	15	0.034	0.190	369	0.568	0.511
D6-D12	29	0.694	0.565	439	0.086	0.283
H	329	**0.612**	**0.478**	484	0.334	0.393
O	0	0.000	0.000	108	0.044	0.022
T	3	0.000	0.001	389	0.331	0.195
I	4	0.001	0.001	1740	0.660	0.564

Supplementary Table 13. Statistics of the PDB 2024 heldout test set

Class	Frequency
C1	3
C2	103
C3	14
C4	4
C5	1
C6	6
D2	13
D3	7

Supplementary Table 14. Protein sequences from the PDB 2024 test set

PDB-ID	SEQUENCE	SYMM
8AND	SKLVGGFSEWKDPDAYTTKIVKAMESKLFEKLSLPNQPEVSFLRYREQIVSGVNYCMRVKIGSDFYDLHIYV<br>PLGSTGDIKSHLIQLTDLHLASELTHSH	C2
8BWA	ETGCNKALCASDVSKCLIQELCQCRPGEGNCSCCKECMLCLGALWDECCDCVGMCNPRNYSDTPPTSKS<br>TVEELHEPIPSLFRALTEGDTQLNWNIVSFPVAEELSHHENLVSFLETVNQPHHQNVSVPSNNVHAPYSSDK<br>EHMCTVVYFDDCMSIHQCKISCESMGASKYRWFHNACCECIGPECIDYGSKTVKCMNCMFGTKHHHHHH	C2
8CJW	TESQIPKMYEMIRDQMRTLASTHKIPLNIDHNCEVIGSIIMAACTNNRDLRPVDKYWFLMGPAGAEVMTEVEI<br>DIQPQLQWAKGAVHDPKYKGQWYPFLALLQISNKTKDTILWQKYPVTQELEISNSLEIYANGHGIKDRLKNSR<br>PRSVGPLVHLLHLKRLQENPPKNPKTKKPLESPAVNGIRKSIVGHLKRQCIGETQKAMINQFEMGRWESLST<br>FAASLLAIKPRIENHFVLTYPLIANCEDFAGATLSDEWVFKAMEKISNKKTLRVCGPDEKWISFMNQIYIHSVF<br>QTTGEDLGVLEWVFGGRFCQRKEFGRYCKKSQTKVIGLFTFQYEYWSKPLKSAPRSIEGSKRGQISCRPSF<br>KGKRPSYNNFTSIDTLQSASGSQTVSFYDQVREECQKYMDLKVEGTTCFYRKGGHVEVEFPGSAHCNTYL<br>FG	C3
8CMP	GHMAEVLVVTSKVKKLIKEKGQMNTSAETIDVLSKAIEQLCLKGVESAKADGRKTVMARDIVIDHL	C2
8CQN	GAMGNKEQKNNNNVKEVSDSVQEDGLNDLYNNQEKQKSFTKNFGERKYEDLINPIEPIIPSESPKNKANIPN<br>ISIAHTEKKETKKENLIPSTNEEKEADAAIKYLEENILKNSKFSELIREVRVIKDEYALIKADLYDVIGKINNKKTSL<br>MENPKNNRDKINKLTQLLQNNLKIDSELEQLINMIDMAENEISSAAFFFDNAQKRLKESIIKRLESKNNRSYAL<br>KLSRQALSDARSALSNLESFASKRIEPMVRKEEIKELIKHAKTVLESLNKK	C2
8FM6	MADPLTPAISDRICKHMNEDAASAIALYAQVFGQQTDVTMAQMQAIDPTGMDLVVESEGGSKTIRIEFEQPLK<br>DSEDAHQVLIAMAKQARSVGKNSAENLYFQ	C2
8FRF	MTTPVLELLEQIQQGSEEQQKEALARLQELLEAGADPNMANSEGTTPVLLLLEIIQQGSEEQQKLALALLQEL<br>LEAGADPNMANSEGTTPVLLLLEIIQQGSEEQQKLAAALLKELLDAGADPNMANSEGTTPVLLLLEIIQQGSR<br>EQQALAMSLLLLLLLAGADPNMANSEGTTPKELLKEIQQQGSDEQRLLAEVLLQLLEAAGGSWGSHHHHHH	C2
8GAD	SMEEEIEEAYDLVEEAEKTGDTSLLKKAKELLDKVAEEATKSGNPILLIRVIIILIKIVRNSGDPSVAALARELLEK<br>LEEIAEKEGNRFIEAMGEALRTQIERAL	C2
8GKV	GGMKNLIAELLFKLAQKEEESKELSAQVEALEIIVTAMLRNMAQNDQQRLIDQVEGALYEVKPDASIPDDDTE<br>LLRDYVKKLLKHPRQ	C2
8HAU	QQANSLLDLMTIRAFHSKILRRFSLGTAVGFRIRKGDLTDIPAILVFVARKVHKKWLNPAQCLPAILEGPGGVW<br>CDVDVVEFSYYGAPAQTPKEQMFSELVDKLCGSDECIGSGSQVASHETFGTLGAIVKRRTGNKQVGFLTNR<br>HVAVDLDYPNQKMFHPLPPNLGPGVYLGAVERATSFITDDVWYGIYAGTNPETFVRADGAFIPFADDFDISTV<br>TTVVRGVGDIGDVKVIDLQCPLNSLIGRQVCKVGRSSGHTTGTVMAYALEYNDEKGICFFTDILVVGENRQT<br>FDLEGDSGSLIILTSQDGEKPRPIGIIWGGTANRGRLKLTSDHGPENWTSGVDLGRLLDRLELDIIITNESL	D3
8IHA	MNSIQIADETYVAADAARVSAAVADRCSWRRWWPDLRLQVTEDRADKGIRWTVTGALTGTMEIWLEPSMD<br>GVLLHYFLHAEPTGVAAWQLARMNLARMTHHRRVAGKKMAFEVKTVLERSRPIGVSPVT	C2
8IL8	MDTPLRDKSYFDERATKEMATHLQQVQRDTRETMAFACRILAMTEQEAGLAGQISVRSERPGAYWTLRFG<br>LGFDEATPEDFIEVDRDLNTLSGEGMANPATRFHLWVYEARPDVNSIIHTHSPWATVLATARQPLVISQMDMT<br>PLHNDCAFLGEWPGVPIADQEGVIISKALGDKRAIILAHHGYLTAGKSCQEATYLSVYLERAARLQVRAQAAF<br>GPLTPVDDTLAAEAHDYLLKPSIVNATFDYWSRQTQGIAPLTKTR	C4
8IRK	MGSSHHHHHHSSGLVPRGSHMMMLAQQWRDARPKVAGLHLDSGACSRQSFAVIDATTAHARHEAEVGGY<br>VAAEAATPALDAGRAAVASLIGFAASDVVYTSGSNHAIDLLLSSWPGKRTLACLPGEYGPNLSAMAANGFQV<br>RALPVDDDGRVLVDEASHELSAHPVALVHLTALASHRGIAQPAAELVEACHNAGIPVVIDAAQALGHLDCNVG	C2

	ADAVYSSSRKWLAGPRGVGVLAVRPELAERLQPRIPPSDWPIPMSVLEKLELGEHNAAARVGFSVAVGEHLAAGPTAVRERLAEVGRLSRQVLAEVDGWRVVEPVDQPTAITTLESTDGADPASVRSWLIAERGIVTTACELARAPFEMRTPVLRISPHVDVTVDELEQFAAALREAP	
8IW2	MSFIFIPTVRTLHQAELVLNHKNTYLRVNSSHMEVPQLVEFIHQLVDKYPGQKIYVDLQGSKIRISRSQPNLILTKDQSVELTIKAPTKDTKAIHIGNPNTIKLLSQGTHVKIDDGRMEIVVNSIKDSETAIATVIKGGELKPGKGFNLQPHPFVQNQLSERDAEIVEKLKDVKEVCFALSFVCVVEEIQDLKKRSNGKYIVAKIEREMDLERLKAISSQCNEIWICRGDMGVQLGFVGMAKFVREYTTFMKQLNCPSIMAGEVMEHLCDNTIPTRSEICYLGNLIADGYNGIVLSDETVFGKYPQQTMDFCYDFVQQYLN	C2
8IXP	MTARTERGRALAFVWLMVEGAQVAAGGVAGYVRNLLDEQDALRDHLAERGWSVEFVLGEPFYDPGAPGYDEERWRRVREHLAARGGRAVRLVSDSDGLDGWGEERFFHALSATGAQLVLDTAERCDAVVAVSGTSAFARVPGMVQRQGGELAAKVLHVHTFGLATHDTAHVPSPAEIAADGDVAFWTRQSDRVSVGYISRYTAELYARTYAIPAAALLPNRSAIPRHAPRFGVLTEERINERIAGLGLPAEGEFVVMWGRNSAPGLDKGYHLLLEAARDLPGVVPVIATRRPDPGLRRLADRYAVPAVLLDDQPFTHLSALLQSPRTLAAAFLGEAEPGAVSPMEAMWVARESGALVIAADTGNLPEVVDDGAAGIVTRRTAADVADAVRRVRKLTADERRRMRAAAAARVRARFDFAANVRELADAAVDRLAEVSKLAAALEHHHHHHHH	D2
8J2W	DILAAGREELMAALAEGDEHAAVDLAMRLLDGGVPADVVLLELVADAQVEIGVLWQANRWSVAQEHAATAISERVIAAVGDRAAAAPTRGHVVVACLDGEWHALPARIVAEVLRGRGWRVTFLGASVPAAHLVPYLEEHGPDAVALSCTLPRGLPRADQVVAACRATGTPVLVGGLGFGPDGRWARVLGAGTWAPTARAAADLLDRPEWPRTALPAPPRPADPEYAALRARRAELVDAGLAALHEWFPPLRDYDARRLDATLDDLGDIVDHLAASVYVDDPELFGEFVTWTAEVLAARGVSPASVEVALEAIARVLDDHPRTRHHLDHGRRALAAHLEH	C2
8J4C	MIWTGLLVGFLFGIVLQRGRIAFNSAFRDVLLFKDNYLFKLAVFTLALEMILFVLLSQVGLMQMNPKPLNLVGNIIGGFVFGLGMVLAGGCASGVTYRVGEGLTTAWFAALFYGLGAYATKSGAFSWWLSWVGQFKSPLSVEESAYYVKGAGPTISSVLGLNPWIPALVIAALFILWAFGTKTTSRETKFNWKIASVCLALVAGLGFITSTLSGRKYGLGITGGWINLFQGFLTNSPLNWEGLEIVGIILGAGVAAAVAGEFKLRMPKNPVTYLQVGIGGLLMGIGAVTAGGCNIGHFLTGVPQLALSSWLASIFFILGNWTMAWILFRRAATPTPVAEAAPSSAEDRVLPFQVATGAVALQTAPRVKKAMANYQVSKEIDVRGEVCPIPDVEAKRAVQSANDGEIILVRIDYPLSKERIPETVKKLGSEVLEIEEAAPGEWNIYIKVKKGSSGENLYFQ	C2
8J4I	MAQNLKDLAGRLPAGPRGMGTALKLLLGAGAVAYGVRESVFTVEGGHRAIFFNRIGGVQQDTILAEGLHFRIPWFQYPIIYDIRARPRKISSPTGSKDLQMVNISLRVLSRPNAQELPSMYQRLGLDYEERVLPSIVNEVLKSVVAKFNASQLITQRAQVSLLIRRELTERAKDFSLILDDVAITELSFS	C6
8J5E	STSDRLKAVEQNLYDVGPRDSGGREGPGHYIAISGNTAAGKTTLIETLAGSLRAAGADAVGVSERVFHHRYLKLMFSASADFAFPIQLSFMLERHLLLLDNLVRRGRTMVMERSHLDDAMFVREHVASGAITAAQQRAYTEVSGELNARIPNPDLIVLMNPEPELSLERLARAEAEGSRPREFPSDAAKRAWVHRWYDLYQELHDDYRRRAVDGDLRGTELLELDAAASPEEKIATVTARARSLVVG	C2
8J6H	RYRVEYHLKSHRKDEFIDWVKGLLASPFVLHAVSHEGDYNDDLATTQRVRSQYADIFKDIEGLIKDKIEFDSRNMSQDEIEDGASSQSLNILGQSRLNLLVPSIGTFFTELPLEQAFLWEDSQRAISARRMVAPSFNDIRHILNTAQIFHFKKQENLHNGKVLRLVTFDGDVTLYEDGGSLVYTNPVIPYILKLLRCGINVGIVTAAGYDEAGTYENRLKGLIVALHDSTDIPVSQKQNLTIMGGESSYLFRYYEDPEEDNFGFRQIDKEEWLLPRMKAWSLEDVEKTLDFAERTLNRLRKRLNLPSEISIIRKVRAVGIVPGERYDEASKRQVPVKLDREQLEEIVLTLQNTLESFAPSRRIQFSCFDGGSDVWCDIGGKDLGVRSLQQFYNPESPIQPSETLHVGDQFAPVGSANDFKARLAGCTLWIASPQETVNYLHRLLETDHHHHHHHH	D2
8JG7	TSLAVTEPEVNDEFTGDKEAYMASVLARYRKTLVERTKNHLGYPYNLDFDYGALGQLQHFSINNLGDPFIESNYGVHSRPFEVGVLDWFARLWEIERDDYWGYITNCGTEGNLHGILVGREMFPDGILYASRESHYSVFKAARMYRMECEKVDTLMSGEIDCDDLRKKLLANKDKPAILNVNIGTTVKGAVDDLDLVIKTLEECGFSHDRFYIHCDGALFGLMMPFVKRAPKVTFNKPIGSVSVSGHKFVGCPMPCGVQITRMEHIKVLSSNVEYLASRDATIMGSRNGHAPLFLWYTLNRKGYKGFQKEVQKCLRNAHYLKDRLREAGISAMLNELSSTVVFERPKDEEFVRRWQLACQGDIAHVVVMPSVTIEKLDNFLKDLVKHRLIWYEDGSQPPCLASEVGTNNCICPAHKAA	C2
8JI1	MEIYLVTGNMNKKEEFLKMMDEELNVEFVNINLEEIQAQDIVEINEHKVKTAYNILKKQDNNKNKKRYVITDDTGLFISKLNNFPGPYIKWMQKALGSKGIADVVSRLDDNTCHAICTYSVYDGKDVHSFKGITNGKIVEPRGNNKFGWDNIFQPESLSKTFGEMTFDEKQNLSPRFKAFVQLKEFLMNEHKKYNNEF	C2

8JRC	AMAQREIRLVDNEYPSPSMTHPPIPLSDIALTGIFEISKILTSPARLEITLANVVNLLQSFLQMRNGVVSLLADD<br>GVPDITVGVGWNEGSDNRYRARLPQKAIDQIVATAVPLVADNVSAHPMFTAADAMALGATDEIRVSFIGVPIRI<br>DSRVVGTLSIDRVRDGRSHFRMDADVRFLTMVANLIGQTVKLHRVVARDRERLMAESHRL	C2
8JS5	APTRVTHPPDDGRGEHFRVRIEGFGVGTWDLDLKTWALDWSDTARTLLGIGQDQPASYDLFLSRLEPDDR<br>ERVESAIKRVSERGGGFDVSFRVAGTSNAGQWIRARAGLIRDEAGTARHLSGIFLDIDEEKQVEGALRTRET<br>HLRSILHTIPDAMIVIDGHGIIQLFSTAAERLFGWSELEAIGQNVNILMPEPDRSRHDSYISRYRTTSDPHIIGIG<br>RIVTGKRRDGTTFPMHLSIGEMQSGGEPYFTGFVRDLTEHQQTQARLQELQSELV	C2
8JSF	GMEQKLYKNYADDIAHYLKQGKKNNLQKGLSYEHFSKNLSSHPKMQWVDKTKNEANFRSLSALNTITGQIT<br>KYEEKLGAHPSFSHLKNTNDSEYHYIVSMFVDVRNSTGLFKKFDPDVVANICRTIQLATIHTCWYFDGYVHR<br>LQGDGLMVYFGGKGTTKQKAVDNALMAASFISYFVKNDLKNLFEEQGVSRIYTRIGLDFGDDEDTLWHNAGI<br>GECSEVTTTSLHTSLACKMQAQAESNGVVVGDNILPYKSSDKNYFTYKKYKKNGSELPYVYEIPEEYFRYK<br>QHDFNWEKFLKNHPQIQEDEDGNLTFINPSLPPNPRVQQNINHLQQNVSGYKPYLR	C2
8JSK	MIERFEGETGMRLLVEALQMNKMVRGNVKIAQELAKKIVLEKVSAGDELIQQDTETNDIYFIISGSLSIIVNGQ<br>QIAIRGPNDHIGEMAAIQPTQKRSATVQAVEQCLVAKITEADFSHLAKNNAELYKSIAQELARRLQERNKLVTT<br>HHLEHHHHHH	C2
8JSZ	GMKPNQFLNSSFSIINEQIEIYERGVTTQKVNQIPKTDDIRIQSSDKPWHWLEIPDLICVFVDMKGSTQLSVTR<br>QDRTMASAYQLFTNTAIQIFHDFDTPYIDIKGDGVFALFNSNQIYRALAATVTFKTFVKEVFTPKIKQKTKGIIVG<br>GHYGIDQKTVLVRKIGLKVNQNRQDPYRYNEVWAGKPINMAAKLASLANIDELLVSDRYFNNLKSDFVLKSC<br>GCTNGIPQENFSELWLPKNVTEENKFDFNKAYSLTSAWCPIHGRFYCQNILNLDNAKS	C2
8JTO	QSSVTLYGVLDAGITYQSNVATPSGSGKSLWSVGAGVDQSRFGLRGSEDLGGGLKAIFTLESGFNIGNGRF<br>NNGGGMFNRQAFVGLSSNYGTVTLGRQYDATQDYLSPLSATGTWGGTYFAHPLNNDRLNTNGDVAVNNT<br>VKFTSANYAGLQFGGTYSFSNNSQFANNRAYSAGASYQFQGLKVGAAYSQANNAGANTTGATDPLTGFNI<br>GGTNAASIQGRSRVYGAGASYAYGPLQGGLLWTQSRLDNLANGAPTIRADNYEANVKYNLTPALGLGVAYT<br>YTNAKANGESTHWNQVGVQADYALSKRTDVYAQAVYQRSSKNANASIYNGDLSTPFSTSINQTAATVGLRH<br>RFHHHHHH	C3
8JVS	GPMPGKKVVARVEEILHDPGRTAPVARVKFEDGTKKLIIAPEGVKVGDVVEVKKV	C2
8JXK	EFMLELAILGLLIESPMHGYELRKRLTGLLGAFRAFSYGSLYPALRRMQADGLIAENAAPAGTPVRRARRVYQ<br>LTDKGRRRFGELVADTGPHNYTDDGFGVHLAFFNRTPAEARMRILEGRRRQVEERREGLREAVARASSSFD<br>RYTRQLHQLGLESSEREVKWLNELIAAERAAPNPAEQT	C2
8JXO	MSECCELCVCQKEPGTFGALIAVNTITAIILVAAGAYMAWKTAAGLGWNTRPHGPEGPPEENWLSPGISILCG<br>VMYAFKAIDWASYNDTGESTAFSLNQVWYSDYLITCPLLVLDFCITVNLRYKLVFSSSIACLLAIAVSTFIVDAP<br>YRYYMYGIGLAGFICAGYALWNEINAQREKIPDSAWWYLSAGRLIFFAGWPFFPLLWTLSFHTSGVINEEWY<br>FILHAILDILCKAVFGFFMLGFRLELEELDFKAIEAEQAKLEGDKEAQALKNDKDTGEVLRHYNRASSGFFGQ<br>GIPDDQGSVSGGSVLMSRARRQHMLYRREASYLSMDPMAEKIRELEMLKKKIQKEVDSRSKKMQREMTAR<br>FAIQDDSDDEDGGRGAGSARRRKNARRG	C3
8JYV	SGRPMFAVAVNEFIRSAGQDSLCGVPDINSSGDFMPLHIIVKEVPKVLPCCRRPKIKRTPYTLNDILDEPCPN<br>QLKSSDLVTFTEPLVSNVKASSSIGLQILKHFDSGAKGSKNFITSASLGTVVKAETIDITKVLAKVRTAKAKVEN<br>DLVSRVMKTKRLCLGLVVETACVAAAGKLTEADNWEISGHTNANIGEAVVTATAELDKNLSRKIEIPPGTALAY<br>SFMDLEILEDRSLRVSSSAGA	C2
8JZL	QHQKASNHSLHNLYNLQRDLLTVAATVLGKQDPVLTSMANQMELAKVKADRPATKQEEAAAKALKKNLIELI<br>AARTQQQDGLPAKEAHRFAAVAFRDAQVKQLNNQPWQTIKNTLTHNGHHYTNTQLPAAEMKIGAKDIFPSA<br>YEGKGVCSWDTKNIHHANNLWMSTVSVHEDGKDKTLFCGIRHGVLSPYHEKDPLLRHVGAENKAKEVLTA<br>ALFSKPELLNKALAGEAVSLKLVSVGLLTASNIFGKEGTMVEDQMRAWQSLTQPGKMIHLKIRNKDGDLQTV<br>KIKPDVAAFNVGVNELALKLGFGLKASDSYNAEALHQLLGNDLRPEARPGGWVGEWLAQYPDNYEVVNTL<br>ARQIKDIWKNNQHHKDGGEPYKLAQRLAMLAHEIDAVPAWNCKSGKDRTGMMDSEIKREIISLHQTHMLSA<br>PGSLPDSGGQKIFQKVLLNSGNLEIQKQNTGGAGNKVMKNLSPEVLNLSYQKRVGDENIWQSVKGISSLITS	D3

8K1B	DFDHVYSGVVNLSTENIYSFNYTSQPDQVTAVRVYVNSSSENLNYPVLVVVRQQKEVLSWQVPLLFQGLYQRSYNYQEVSRTLCPSEATNETGPLQQLIFVDVASMAPLGAQYKLLVTKLKHFQLRTNVAFHFTASPSQPQYFLYKFPKDVDSVIIKVVSEMAYPCSVVSVQNIMCPVYDLDHNVEFNGVYQSMTKKAAITLQKKDFPGEQFFVVFVIKPEDYACGGSFFIQEKENQTWNLQRKKNLEVTIVPS	C2
8K3G	MKPDIALTGLLTVLAAASAAAQPVNNNNNNKNAPFGYKSGSPESIKNLKDKIQNVVWILLENRSFDNILGGFKRPGFDNPANNGPFCIPQNVSNPNSPKWCTKAKDFDSVLNDPSHSVTGNNMEFYGTFSPDNAAIASGKLQPSQQGFVDMQLVSYPKLDPQVAAEQVMGYYTEDEIPTIANLVDEFTVFNRWFSCVPGPTNPNRLCALAGTAAGHGTNDNSFDVSGIDIKGIFQVADEKGVSWKNYDGTNGAFLPDALFFNYTAKYKKQNVVPLENFFQDAYLGLLPQLSYINPSCCGLDTNSMHPTGNVSFGQVFVKQIYEAVRNGPQWDKTLILLTYDETGGFYDHVPPPLAVRPDNLTYTEKAPDGSTYTLTYNRLGGRMPTFLISPYAPKGYVEQEGIDPATGNSSVYSATSVLKTLGYLWDLEDLTPRVSHSPAFDHLIGPQLRSDTPTTLTTPHTFPTDV	C2
8K48	HHHHHHAENAGIYGSQSRDDFDRDDVEQYFNYMGMLAVEGTYSKMEALLNLNIHPVDILLMLAATEGDRPKIEELLKAGADYSVKDADGRTAIDRANSEEIRDLILGYSTQKA	C2
8K6Q	GPGSEFMDEKTKKAEEMALSLTRAVAGGDEQVAMKCAIWLAEQRVPLSVQLKPEVSP	C2
8K74	MPKRLGFFTRLLDQGSAQTRYRLAAEQIRHAERVGFDSAWIAQHHFHEQEGGLPSPLVFLAHVAAQTDRIRLGTAIITLPMENPLRVAEDAAVLDLLTDGRLEVGFGSGGTPTSFLPFGLTSEQRGAVFADHLHLIHSAWRGDTLSHPDNHLYPPAPQLAARIWIATFSVEGAIRAGQAGHGLMLSRTQPRPPGEPRLPLDAIQNPIIDAYLSALPDGVAPRILASRTAFVADSHAHALQVAEPGLRKQAAQHREAGHRIEGDSVTDYLQQLDAHVGDPEHVIASLAQDSVLARATDISFQVHSVEPSHRDTLRSIELIAQHIAPHIR	C2
8K7M	NNMFLVVALDGGREADAVAVMKAAKERGIKIILWLAGDVERLKRLFEKAKELGTDIAGIILDGAPLEKLRPVIKLAAEFGAALFLANMPDAATAEEAIKIAKEEGLEVYLLADLDNLDTVLALAKKYGAKVIAKVDKVEDLKKIVEKVKAHGTDILAGILISPLKPEMVDTLKKAIDELPGVKTVFLSGVSANPALAVEVTKFLLEKGIAVGVLERVPPEEVVALLDAGALEHHHHHH	D2
8K89	GEFMPDEKKDAMYWEKRRKNNEAAKRSREKRRLNDLVLENKLIALGEENATLKAELLSLKLKFGLISSTAYAQEMQKLSNSTAVYFQDYQTSKSNVSS	C2
8K8F	GLEAAVDAAYEILLEELEKHGVRTIVVVGTGELALVLALAGVRLARERGVKTIVLVRDAAAAHRLLAALAAALGLPAPASADAAALAAADAALWAEHGLRVRVADLTDPAALRAALEALFAEHGRDDTLVLPAGEAALAALEPVLRELGLEEMAAVAREVYARLRAALAAARALEHHHHHHYARLRAALAAARALEHHH	C4
8K8G	GAEAAAAAAVTAELRAFRAAGGTVELEDLPVTPETLARAEAALARLPPESVAVETYTVPAPTPEAFLAALEAALARLAAEGLPAILLRVVDADGNLVGSILVAAAGPPAESAAATGRVLTIYVASSPEGLKVARGLAIETRDAGGLALAIGASGAWALAGLAGALALARRLAEAHGAPVRVVTIGDPANPTDAALAAAIRAAYAAALEHHHHHH	C2
8K9C	GAMDSPLQSTLSSAASPSQAYETYIENGLICLKHKIRNIEKKKLKLEDYKDRLKSGEHLNPDQLEAVEKYEEVLHNLEFAKELQKTFSGLSLDLLKAQKKAQRREHMLKLEAEKKKLRTILQVQYVLQNLTQEHVQKDFKGGLNGAVYLPSKELDYLIKFSKLTCPERNESLSVEDQMEQSSLYFWDLLEGSEKAVVGTTYKHLKDLLSKLLNSGYFESIPVPKNAKEKEVPLEEEMLIQSEKKTQLSKTESVK	C2
8KA6	GKEELEKLAKELSKVWPELGKLVEEVIKLIEGRSKDPKAAVEGLIETMRRAADLLIEKVLELNPALKDDPARTAALVERLLAGTGEIPSFLSEAGRVLAEAAVAMREAADRLRAELAAGNEDLSAAADEALAVFVEAVRRVAAALLEHHHHHH	C2
8KBB	SSGLVPRGSHMEIKNGLCTQKYTKVYAEDKEKWKFNAPHHFIVGKADCEDEYIEPIEYVNFQEGPIKEYGINGVNNEDLILMVITRLQAFQDSPYKCRENAMAITKLQECLMWLGKRTLDREVKGIEGTSEI	D3
8KBE	MGSSHHHHHHSQDPMKELSTIQKREKLNTVERIGSEGPGGAYHEYVIKSNSMDSQGNYDVYETIKFQKGARKEEKSQHGVIDSDLLEIVRDRLKSFQAGPFSSRENACALTHVEEALMWMNRRVEDRIERNVLGTNTK	C2
8KBG	MKELSTIQKREKLNTVERIGSEGPGGAYHEYVIKSNSMDSQGNYDVYETIKFQKGARKEEKSQHGVIDSDLLEIVRDRLKSFQAGPFSSRENACALTHVEEALMWMNRRVEDRIERNVLGTNTK	D3

8KBI	SMTFGQALESLKRGHLVARKGWNGKGMFIFMRPEDSLPTNMIVNQVKSLPESFKRWVANNHGDSETDRIK<br>FTAYLCMKAADGTIVNGWLASQTDMLANDWVIVE	D2
8KBJ	SMTFGQALESLKRGHLVARKGWNGKGMFIFMRPEDSLPTNMIVNQVKSLPESFKRWVANNHGDSETDRIK<br>FTAYLCMKAADGTIVNGWLASQTDMLANDWVIVE	C2
8KC1	GKVVLDAVTHPSKIEEAEKLLEEYRERLGGGLEGRVIADPKADPNTGNVHLKTEDGFEVDSTGKDIKTSLDA<br>ALAALEWLEHHHHHH	C2
8KC8	GVGLHFRGAEPWEEKIRAAAELGVTTVRLDATGPIEETVARIRAAAEAGITNLVLEPGPLALDELKAYRDAAP<br>DTFLTVHIGDNPVLDDATIADIKAAGADAIGIPAKNIDDLIANLEKAKAAGLKVLVGLLPGPKELVEEKVRAAAAA<br>GAAALLLDLSDLEVAKAAIRVADEAGLPVLAGGGFDDVDSFVAAAEEIRALNPRATLLLEARGLATADIVAAME<br>RALEHHHHHH	C3
8KCE	MGSSHHHHHHENLQFQGGGNRGMSRNQDAYAEKMDMTLDALNFLLGVEHLASAFYVQAVNNFTADDFKA<br>AGLAQRDYDQFVGVRNNEVDHRDTLISVIKSLGGKPNPPCKYTFPVTDVASVLKVSRTLENADKPAYLGALR<br>DIKSVELRTSVQGALSGDSAHAAFFAYLTGKAPAPGPVDGPLTQRHIATLAQDFIVSCPYPAPKPFPKLTLSP<br>QSGPVGTVVATTCAQDVDTNGVMCAIISGNQGTLMQRPGQAKDGSGAATCTIPPGVKGILFIAWVRGRDVL<br>NVGVDDSSTVCGPNYFLLSALGDAVPGV	C2
8KD8	MATQSSTELPQINMTTAEPTSANKRTYPWVKLPHPYLTSYAIHVVSESTPRVLQLRLHDDQKGQALPEPLHS<br>ASLTYTDIAFDEAAQEIPDNDNSPWARSRRAPGTSFHWTGQEPPTLGQIWNVIHALFLTYPQHEIVRLDLNG<br>SGKDIIREECLRTGLAVPFPSPRVPFGTENRPSETDTLILLRSAFWQGAGSPVGPR	C2
8KDK	MSDLAAVDDWSTLRRIAIDAVSTGRNPELKWLADHPEGTPAYALHLADPLEGAPEGLRQCLREAWDEPLDS<br>YVLSHHGLPELRQAMERWFADDENWPRRRRLLTTATMTGTGPAMYDLLRTIKAREPEGPMAALVPRPGWD<br>YRLFAHDVGYEPIGYHVPFTSPTGPEPGDLDRAVEQTRAKGLRPTVLVLNPQHYATGGNWTPEFVRYALSL<br>ADTLGMWVLVDNAYHGMTAAGTQPTSTVRLALDGGFEERLIHVRTLGKQFACNGWAVGSVTAMPDVIDEFA<br>HRWRGFREYPGHAREQAAFAGWLNNPESRKWADERREAIRSNGDALLDALAEVSNTTRHCHGGSPFVLF<br>EVPGGWSQEDFRQRLFADTGVLLASAQIPYAPDWVKVFLGRRPDRFLPAVEALRTRPSRAWQPRLEHHHH<br>HHHH	C2
8KE9	MTNIVGRIGFLTTGKLGIKLRGVLIDESTTPNTTYLPGIESFFNITANVLTSVSYPETETQNVTATFSIYSVDGSS<br>NPVFPALLSFDAIVPNVASVEFDVLAPTGVVNNQLDTSALRIAKIIANDPALAQKVAGAPYPRGAYSATETYLY<br>GEMVSYFGKNYISKSLSPIINILPTVTDSWYELVITLPESVSVIATGSDTAYGTGWNGSLLVPTQNAVYDKIVTV<br>DAAIATANTNITNLGTAKADLSYVNTQLSADQVVLDALSSGKADLSYVNTQLNSKANLNGAVLVNATTATPPIS<br>DNDTSLATTQHVRSFNHSRLAFNAFRGGQQGVPSLSYVTTTAQFNSSSVRSGWGDNFSSNRWLVGEGGT<br>YLITVTTRFATVGGTPPTYFDALLFVGLSGSGVENFLTRSQSVYPSFGYTLSWVGILTFNTGQNVFLNYQVNA<br>VGGGSYSVVLEDVRFSGIQLG	C3
8KGS	MEAFEISDFKEHAKKKSMWAGALNKVTISGLMGVFTEDEDLMALPIHRDHCPALLKIFDEIIVNATDHERACH<br>NKTKKVTYIKISFDKGVFSCENDGPGIPIAKHEQASLIAKRDVYVPEVASCHFLAGTNINKAKDCIKGGTNGV<br>GLKLAMVHSQWAILTTADGAQKYVQHINQRLDIIEPPTITPSREMFTRIELMPVYQELGYAEPLSETEQADLSA<br>WIYLRACQCAAYVGKGTTIYYNDKPCRTGSVMALAKMYTLLSAPNSTIHTATIKADAKPYSLHPLQVAAVVSP<br>KFKKFEHVSVINGVNCVKGEHVTFLKKTINEMVVKKFQQTIKDKNRKTTLRDSCSNIFIVIVGSIPGIEWTGQR<br>KDELSIAENVFKTHYSIPSSFLTSMTKSIVDILLQSISKKDNHKQVDVDKYTRARNAGGKRAQDCMLHHHHH<br>HHH	C2
8KHG	MSESAFAPWIGRQEETHDQLSRNLVKRIAATFGEPTPAHGEALPPLWHWAFFQDPVEAAGLGVDGHPARG<br>GFLPPADDRNRMWAGGRLEFHQPLRVGGEASRTSTILRVEEKHGRSGALLFVTLRHDYRQDGQLALSEEH<br>DIVYREPTPPKLGGTEALPEGDWREALEPDPVLLFRYSAVTFNGHRIHYDWPYVTDAEGYPGLVVHGPLIAT<br>LALRAFCRANPQARLRRFAYRGLRPLICPEPFEVGGRLLAAGKAEVWVGNGAGLAQRGDVEFD	C2
8ON5	MDNKNNKPTDQEILKTSRAVGEIPSADNLKNRFKARSIPLETDFTNLIDLAEVGRLAIGQSPSQQSKTPGTGM<br>ELTSDGKLQVKAGAGVDIDNNNRITIKSGHGIKVDGNGISVKPGSGIKVDSNGVNVNIDDFWEEIRNKIMPKG<br>TMLPIYGTPNPSALPTGWEWCDGKDGRPNLKKGKYNLLSGQSSGTDTFWADNKNGDTEINVLFVYYMIKV<br>V	D3

8OS5	IPPWEAPKEHKYKAEEHTVVLTVTGEPCHFPFQYHRQLYHKCTHKGRPGPQPWCATTPNFDQDQRWGYC<br>LEPKKVKDHCSKHSPCQKGGTCVNMPSGPHCLCPQHLTGNHCQKEKCFEPQLLRFFHKNEIWYRTEQAAV<br>ARCQCKGPDAHCQRLASQACRTNPCLHGGRCLEVEGHRLCHCPVGYTGPFCDVDTKASCYDGRGLSYR<br>GLARTTLSGAPCQPWASEATYRNVTAEQARNWGLGGHAFCRNPDNDIRPWCFVLNRDRLSWEYCDLAQC<br>QTPTQAAPPTPVSPRLHVP	C3
8OX2	GSEHLVTTATFSIGSTGLVVYDYQQLLIAYKPAPGTCCYIMKIAPESIPSLEALTRKVHNFQMECSLQAKPAVP<br>TSKLGQAEGRDAGSAPSGGDPAFLGMAVSTLCGEVPLYYI	C3
8P4W	MATFTTEQAGYQMQAILQVIGYDLLIVVTGGTNPHIGDVTTLTASTVPETVKFPSHDGRFHKDNFISERMAKR<br>IQRYLAGSCTITAGIHVNQITKAQIAAAAPMTDDLSRQIISWLQAHPVQAEKPEYYGQDEQPR	C2
8PHA	MNHKVHHHHHHIEGRHMTTLSPSEGKAQIRALLNLINTSAEQAIAEYDKQECDIPSLTSGEPHPMDDRLPSL<br>ELKNTLRILEGACAQLCVTLAPPAHTMLNYSMDVLVPSCISTVIQAGVAPLLAKHPKGLHIDVLSKETGIHPQK<br>LATILRLLILNYCFQEVESNVFANNRLSLTLLPETSVVDILDLKTGEMHRKATLWVYDALVDPDFGPTYDGNKS<br>PLVYALRREGFDGSLYDYLQTQPGAVARFARAMLGFSVSRGLMNLLNVFPWQELAPGSTVCDLGGGNGNT<br>SIEIAKKFPHLKVHLQDLPDTIEEAKVFWKEEYPDAIKDSRVAFTPIDFFKQAPVPDQDIYYISQIVHNWGDED<br>CITLLKNIRSAMSPKSRLLINDYLASHLDKTSIANQHPSLPRAPYPLSPGFGRGMARTYTGDYTMLVVCNSRE<br>RSLEDFIELCSAADLKFVRVWDLAETSVTEFVPAHHHHHHSR	C2
8PJQ	GSHMNGLIYAVGGYDGNTHLNSVEAYDPERNEWSLVAPLSTRRSGVGVAVLNGLIYAVGGYDGNTHLNSVE<br>AYDPERNEWSLVAPLSTRRSGVGVAVL	C3
8PKS	GSHMLLYAVGGFDGTNRLNSAECYYPERNEWRMITAMNTIRSGAGVCVLHNCIYAAGGYDGQDQLNSVER<br>YDVETETWTFVAPMKHRRSALGITVHQGRIYVLGGYDGHTFLDSVECYDPDTDTWSEVTRMTSGRSGVGV<br>AVT	C2
8PME	GQQANSLLDLMTIRAFHSKILRRFSLGTAVGFRIRKGDLTDIPAILVFVARKVHKKWLNPAQCLPAILEGPGGV<br>WCDVDVVEFSYYGAPAQTPKEQMFSELVDKLCGSDECIGSGSQVASHETFGTLGAIVKRRTGNKQVGFLTN<br>RHVAVDLDYPNQKMFHPLPPNLGPGVYLGAVERATSFITDDVWYGIYAGTNPETFVRADGAFIPFADDFDIST<br>VTTVVRGVGDIGDVKVIDLQCPLNSLIGRQVCKVGRSSGHTTGTVMAYALEYNDEKGICFFTDILVVGENRQ<br>TFDLEGDSGSLIILTSQDGEKPRPIGIIWGGTANRGRLKLTSDHGPENWTSGVDLGRLLDRLELDIIITNESLQ<br>DAVQQQR	C3
8PUZ	MDKLREKINAARAETDEAVARAEAAEAKLKEVELQLSLKEQEYESLSRKSEAAESQLEELEEETKQLRLKAD<br>NEDIQKTEAEQLSRKVELLEEELETNDKLLRETTEKMRQTDVKAEHFERRVQSLERERDDMEQKLEEMTDK<br>YTKVKAELDEVHQALEDL	C1
8Q28	GGSLINFTDGFESTGVNQQPSGWGNFVGWQSNNPNNNIGQSVYALVDNTRAFTGNNSVHFKGGAAPAQIV<br>RTLPAGLDKVYLKAMVYMSKKLGNEAGDNHEHIFGVRGNVAQADNEVRFGQIKGHVGTNEMPSDDISPPQ<br>SQWYSGPEIAADTWHCVVVEMLGGNRPYHQLHAYLDNQLIHSIDSISDWNNGGVNGNTQWLDGKLNYAFF<br>GWHSFSNNNADVWMDDIEISDQPISCDSRELEHHHHHH	C2
8QGR	MEKPYMIGANSNPNVINKSTTYTTTTQADEQDKPKYTTRLEFDTIDMIRFINDRGIKVLWEEAYFCPCLNPDT<br>GHPRVDCPRCHGKGIAYLPPKETIMAIQSQEKGTNQLDIGILDTGTAIGTTQLEKRISYRDRFTVPEVLMPQQ<br>MIYFVNKDRIKKGIPLYYDVKEITYIATQDGTVYEEDYEIKNNRLYLNEKYENHTVTLKILMTLRYVVSDILKESR<br>YQYTKFNQPKSKFENLPQKLLLKREDVIVLQDPYKVNDGIEEDLEIQVDDPKASASNPSNLGGFFGGAFK	C6
8R4Z	MNLLDSIKSENTGFETTLIKGIEPIRQFVLAISIYHLFDTKLFSLLIKHEVASPEVACNELGMEKEKLLGLFRYLK<br>NEGILLETIDGFSLSKEGHALAPFEGWYVMLVGGYATTFLQMGERLQEGAGWATRDATKVGVGSCGISHFD<br>AIPLTRSLMAQAPGTCTKLLDLGCGNGRYLAEFCKALPQIQAWGAEPDRGGFEEAVDLIEKEGLSHRVHISH<br>SGAVEFLDSDFDFEPDFIVLGFVLHEILGQAGRPAVVNFLKKIVHRFPAINLIIIEVDNQFDNAGAMRHGLALAY<br>YNPYYLLHCFTNQLLVQDADWLDIFAEAGLSLVTRETTSDQVDSTGLEIGYLLRRA	C2
8R9R	MGSSLMTEHKKRIDPVGAMLESKLLEAEFSPAVAAKLAALSQHYTPAELVRALPQSLANMLDNQGDDIVRQG<br>GVVALVGPTGVGKTTSLAKLAARFAAHHGPEQVALITTDHYRIGAYEQLATYGKIMGCPVKQAHDLNELEQIL<br>YQFRNRKLVLIDTAGMGQRDMRLYQQLDNLTANSRIPIRSYLVLSATGQRRVLQDAVNHFKRIPLSGAVLTKL	C2

	DESVSLAGALSVLIQSGLPLSYVTDGQRVPEDMKVADTLMLAQQALATLDSTEQQSLQDTAWSDNMACAFE<br>HHHHHH	
8RC6	AENYHLKWDSHLTYLNSSIATLYKNEKFADVVLYSSYNSSGIPSDIPTVGISAHKFILSASSQFFATMFETAPIT<br>NPNGVLYVVLPPDLSHRAIQILVQYMYSGEATVSNDILNEVLRGGEILKIRGLCRT	D3
8RK4	MTLYMGPNTGLLINGLPGEGHYSDLIRMWRWDDFLRQPVVKGRVATLPTTGQAEGDTYIFTGSGSNQNRL<br>ARWWATGATTAIWEYMPPRLGWRVQVANETTPSGQVKTYEYSGTAWVELVGGMSDAPSDGKAYARESGA<br>WTELGSAAKSALNVLPFMNLMPDMGRFAGTAANPLATMFTTSWTPSSFLNGWNGATVADGGKFAFDNSTN<br>GGAGPALNARVQALLAAMGRTWTSVSRYGVEFFTAVLTAGSQTTTGSAGADGVTRYLCCSNGSKTVFNAG<br>GWATVVMWLRVESGSAHISSAPYTTHRLWINGAVAAPGVVLPANQWVHLRFSMQSYNGYDNACPYIYASA<br>GAQIAFACPAWFGGLVDPGIHVAPILTINGASA	C3
8RK8	MAQETYFYGQGEIDAAPIVNGVLGKWRWIQDVSAMSIQLAVEKVEHKESYSGQKALVRSFPIGKTATVNITL<br>HSIGPDNLALTLYGKVVAKAAGSVTGEVLPADLVAGDVIRLANFGVSELVITDSASSPAPLDPQYYALRADGAY<br>GEVQLLGLPTPAPTQPFKAAYEYAATKQVGMFTAPQPTVALRYKGINLAEGGAPVIVELYKVATDPLQELALIS<br>DGNTVAGMQISGGILLDTSKPDTGDLGRFGRIIQLG	C6
8RK9	MSDPFDYLFLEPLLIERIRSEVPGLAIVSGVPDLATLSEQDQPAPSAYVVYLGDETGTGADHQGGQRAIQTV<br>GQQWAVVLVVHYADSSNSGEGARREAGPLLGRLVKALTGWAPAIDVAPLARSARQSPATYASGYLYFPLVFT<br>ARFVYPRIKSWKP	C6
8RKD	DYYHATKTTIFNALLNTIDLSQLAQLDLKQAGEEIRDIVAELVAIKNVSMSVAEQEHLVQDIINDVLGYGPLEPLL<br>ARDDIADIMVNGAHRVFIEVGGKVQLTNVRFRDNLQLMNICQRIVSQVGRRVDESSPICDARLPDGSRVNVI<br>APPLALDGPTLTIRKFKKDKLTMKNLVEFASISPEGARVLGVIGACRCNLVISGGTGSGKTTLLNTMTAFIDPT<br>ERVVTCEDAAELQLQQPHVVRLETRPPNLEGSGAVTMRDLVKNCLRMRPERIIVGEVRGPEAFDLLQAMNT<br>GHDGSMGTLHANSPREAISRIESMITMGGYGLPSKTIKEMIVGSVDVIIQAARLRDGSRRITHITEVVGLEGD<br>VIVTQDLFVYEITGEDEHGKVVGKHRSTGIARPRFWDRARYYGLERELAEALDAAEA	C6
8RON	GSHMSVTAGIIIVGDEILKGHTQDTNTFFLCRTLRSLGVQVCRVSVVPDEVATIAAEVTSFSNRFTHVLTAGGI<br>GPTHDDVTFEAVAQAFGDELKPHPKLEAATKALGGEGWEKLSLVPSSARLHYGTDPCTGQPFRFPLVSVRN<br>VYLFPGIPELLRRVLEGMKGLFQNPAVQFHSKELYVAADEASIAPILAEAQAHFGRRLGLGSYPDWGSNYYQ<br>VKLTLDSEEEGPLEECLAYLTARLPQGSLVPYMPNAVEQASEAVYKLAESGSSLGKKVAGALQTIETSLAQYS<br>LTQLCVGFNGGKDCTALLHLFHAAVQRKLPDVPNPLQILYIRSISPFPELEQFLQDTIKRYNLQMLEAEGSMK<br>QALGELQARHPQLEAVLMGTRRTDPYSCSLCPFSPTDPGWPAFMRINPLLDWTYRDIWDFLRQLFVPYCIL<br>YDRGYTSLGSRENTVRNPALKCLSPGGHPTYRPAYLLENEEEERNSRT	C2
8RPR	MATHDIAAQHLADGIAASGPAPDLAAAAAFLEMGDRLGVVAHLDPDRTLETAEVAAALDLPEPALVRYLDAVE<br>SAGLVIREGEGRYRACPDFDTIRHQAGYISWTMNANRPFIENARDFFTDWDKAARTHVRDYREVAVSSQW<br>MGSHAFYPTALATIIDAAPRKVVDLGAGTCRLLIEVLGAVPGSTGVGLDFAADACRAAEQAVAQAGMTDRLT<br>VVERTIQSVATDPGVLEGADVIHAGFVFHDMLPEEEDVCDQVLANCRESLAPGGFLAITDAVPYLRNDRERR<br>FSAAVSYYHGEFMRRRLQSEEEWVERLRGAGFSDVRALTLAFPTGRLFLAHR	C2
8RQJ	GPGADRAQDHNNNNTPRNSNYVVEEEEVSEEEEEAIMMPDFGDHVDTSIFGQILEMDEGDDHDFSAPLVL<br>NFFEQAEETFQKMETALNNKDLPELSKLGHFLKGSSATLGFTKIRDSCQLIQQYGHGLNVDGSSEPDEGVC<br>LKKIAEALASAAVDTVALHKMMREFFEY	C2
8RUA	GIDPFTMTPSEDFVVTDRGGIVENSHRVHAAVVDAKGRLLYALGNPTRMTLARSAAKPAQALAILETEGVAG<br>YGFDDADIALMCASHSSEDRHIARTRAMLSKIKAEEADLRCGGHPSLSEMVNRSWIKQDFIPTAVCSNASGK<br>HVGMLAGARAIGAGTDGYHLPDHPMQGRVKRTVAELCDLDAGDVEWGTDGCNLPTPAFPLDRLGRIYAKL<br>ASAADGSDAGEGQSTRCAALAHIFRAMARHPEMVAGEGRYCTMLMRAFDGALVGKLGADASYAIGVRASD<br>ATRQLGTDGALGISVKIEDGNLEMLYAVVTELLERLGIGSPDVRSQLASFHHPQRVNTMGVTTGGVSFPFKL<br>RGSKSNVDDPRLAAVAR	C2
8RVS	MNLLDSIKSENTGFETTLIKGIEPIRQFVLAISIYHLFDTKLFSLLIKHEVASPEVACNELGMEKEKL<br>LGLFRYLKNEGILLETIDGFSLSKEGHALAPFEGWYVMLVGGYATTFLQMGERLQEGAGWATR<br>DATKVGVGSCGISHFDAIPLTRSLMAQAPGTCTKLLDLGCGNGRYLAEFCKALPQIQAWGAEP<br>DRGGFEEAVDLIEKEGLSHRVHISHSGAVEFLDSDFDFEPDFIVLGFVLHEILGQAGRPAVVNFL	D2

	KKIVHRFPAINLIIIEVDNQFDNAGAMRHGLALAYYNPYYLLHCFTNQLLVQDADWLDIFAEAGLS LVTRETTSDQVDSTGLEIGYLLRRA	
8RWU	SLNEHEGEVAYDKKEDAEISMHMNEQDKLDVPSLVEICKQQLIVILKDMCADSNSSDEKASFMY HLNRLRSAVTVVDLHNYIAVFGPCLSYNKLPSTWNISVCDYLKQQLNILRAADSQQSSSNHVSY LELHNDYEDIIHDKKGNATTTASNSMQGNMNSNNLNSQLSMKGSSIHMNSANSTSNVSGNATG NASGHISINA	C2
8RXA	EYKTNFIDLTREALSLILQDLKNNVIPKIPVGIEKRERYKNSLRLCLKSARNTQHMNELEPYLELF SECIKNSKLPSHMSLKDQLFYLDKLLENLYFQG	C2
8RXO	METLVNERDINIKNENSKDNNKEMMIGMSHMNHDDQDLIYEKKDDIESLRLHYTDQEKLDVPSL VEICKQQLIVILKDMCSDCSTTDEKTSFLYHLNRLRSAVTVVDLHNYIAVFGPCLSYNKLPSTWNI SVCDYLKQQLNILRAADSQQNA	C2
8S1J	GARTKPDKWIRDEIERLDPHVDYARIWQLTMTYYVDDFLMNLIYTLGIPAFTQPPLGSIMMGQVT RKAVDHGQKRADDTLQHFWRWFEYGPADERAQASLAQVNKIHQALAKRQPGTFPARDVIYTS SWIGVAFHRLRLAAGLPGLSDKQRIAAHHFWAGFGSIFWSEDGYVTNYPDSFEAMLKFVEDYE AEDWEKVESGRILGQAINEQFYDAYFPGQLRALGEQLVLSLQTPGIRRLMDMGDPDPQAQKIV LMMLNQYLTLIEDVLPDPELSRPERARLEGIRPPQHIDPPIAKILCPFKGISH	C2
8S4S	MSKYERPLKRESQIKEFELGTHAAVIEKVQKKRSQKGNDMFLLSLLGKSNEKGVYFLTFGNDYT EDNLRYILASIQDNGVEIPDVDFGYNRETFEFLKGKDVYIQVEEQEYKGKVKHAVTNFLTQDEFE ESEEMEFSESNTEEDW	C2
8S86	WEYGDLHLFGPNQRPAPCYDPCEAVLVESIPEGLDFPNASTGNPSTSQAWLGLLAGAHSSLDI ASFYWTLTNNDTHTQEPSAQQGEEVLRQLQTLAPKGVNVRIAVSKPSGPQPQADLQALLQSG AQVRMVDMQKLTHGVLHTKFWVVDQTHFYLGSANMDWRSLTQVKELGVVMYNCSCLARDLT KIFEAYWFLGQAGSSIPSTWPRFYDTRYNQETPMEICLNGTPALAYLASAPPPLCPSGRTPDLK ALLNVVDNARSFIYVAVMNYLPTLEFSHPHRFWPAIDDGLRRATYERGVKVRLLISCWGHSEPS MRAFLLSLAALRDNHTHSDIQVKLFVVPADEAQARIPYARVNHNKYMVTERATYIGTSNWSGNY FTETAGTSLLVTQNGRGGLRSQLEAIFLRDWDSPYSHDLDTSADSVGNACRLL	C2
8S9K	GHMGKVTKNSSSIKVVKLLVRLSDSVGYLFWDSATTGYATCFVFKGLFILTCRHVIDSIVGDGIEP SKWATIIGQCVRVTFGYEELKDKETNYFFVEPWFEIHNEELDYAVLKLKENGQQVPMELYNGIT PVPLSGLIHIIGHPYGEKKQIDACAVIPQGQRAKKCQERVQSKKAESPEYVHMYTQRSFQKIVH NPDVITYDTEFFFGAAGSPVFDSKGSLVAMHAAGFAYTYQNETRSIIEFGSTMESILLDIKQRHK PWYEEVFVNQQDVEMMSDEDL	C2
8SBH	MIRIYASSTIGNYILPAVIARYRHDYPQLPIELSVGNSQDVMQAVLDFRVDIGFIEGPCHSTEIISEP WLEDELVVFAAPTSPLARGPVTLEQLAAAPWILRERGSGTREIVDYLLLSHLPKFEMAMELGNS EAIKHAVRHGLGISCLSRRVIEDQLQAGTLSEVAVPLPRLMRTLWRIHHRQKHLSNALRRFLDYC DPANVPRHHHHHH	C2
8SBQ	GPGMETLELQGAKLRYHQVGQGPVLIFIPGANGTGDIFLPLAEQLKDHFTVVAVDRRDYGESEL TEPLPDSASNPDSDYRVKRDAQDIAELAKSLSDEPVYILGSSSGSIVAMHVLKDYPEVVKKIAFH EPPINTFLPDSTYWKDKNDDIVHQILTEGLEKGMKTFGETLNIAPIDAKMMSQPADTEEGRIEQY KRTMFWLEFEIRQYTHSNITLDDFTKYSDKITLLNGTDSRGSFPQDVNFYINKETGIPIVDIPGGH LGYIQKPEGFADVLLNMWG	C2
8SDE	GPTVEEAKAEKETELSLQKEQLQLKIIEIEDDVEKWQKEKDRIKSFTTNEKAILEQNFRDLVRELE KQKEEVRAALEQREQDAVDQVKVIVD	D2

8SDI	GPGGVEALEDALAQIKSVNNALQERVEAVAADVRTFSEGYIKAIEEHRDKLLQQLDDIRIQRETA LQLQKAQLEQLLADMRTGVE	D2
8SDJ	GPSGGEVALEHKKKIQKQLEHLKKLRKSGEEQRSYGEEKAVSFLKQTEALKQRVQRKLEQVYY FLEQQEHFFVASLEDVGQMVGQIRKAYDTRVSQDIALLDALIGELEAKE	D2
8T7L	GFDEFPIGDEQDAGILTVAGVYFQPVDMEPAGNSLSKNEADCHMEADISANEKGATLGYGAGD FVPYLHVKAYIQKVGSSKVQEVAFMPMNASDGPHYGANVKFEEGLGKYNIKFEIKAPGNDYLL HVDKETGVTGRFWTEPIVVEWKDFEWTGPQW	C2
8TDF	MKKFGHTAPSDKLNILGVGIGGRGSSVLRGLESQNIIGLCDVDWKYADHVFKRYPAAKKYNDY RKMFDEMLKSADAVMVATADHTHAIIAADAMTAGKHVYVEKPLTHTVYESRLLTKLADKYKVATQ MGNQGASDEGVRKVCEWIWNGEIGEVRKVETFTDRPIWPQGLSRPEDDQRIPKTLNWDAFIG PAPYRPYNAIYTPWNFRGWWDFGTGALGDMACHILHPVFKGLKLGYPTKVQGSSTLLLNESA PMAQTVKFVFPARDNMPKVAMPEVEVYWYDGGLKPARPEGLPAGKDLNMAGGGVIFYGTKDT LICGCYGVNPYLVSGRVPNAPKVLREIKESHQMDWVRACKEDADDRVPSASDFSEAGPFNEM VVMGVLAVRLQNLNRELLWDGPNMRFTNIPDDATISAVIKDGFHIKDGHPTFDKTWTDPVNAQ QFAQELIKHTYRDGWKLPDMPRHHHHHH	C2
8TDI	MEKVRYGIIGVGNQGGAYAGFLTGTGNVPGMPAAPCPPHCALGALCDIDPQKEEMCKEKYPD VPFYKDWKDMVASGDVDAVITTVPHYLHTEIAIYCLEHGMNVLVEKPAGVYAKSVREMNECAAA HPEVTFGIMFNQRTNKLYQKIREIVASGELGEIRRSNWIINNWYRPDSYYRLSDWRATWGGEG GGVLVNQAPHQLDLWQWICGIPTTVYANCINGSHRDIAVENDVTVLTEYENGATGSFITCTHDLL GTDRFEIDLDGGKIVVEDSKKAYIYRFKETETAVNARDMDWMQIAMLTSSNGNSDDKMFEVEE FENTDGWGYQHTTVMENFAQHIIDGTPLLAPGSDGINGVRLANAIQLSGWTGEKVANPVDEDK YLAELNKRIEAEGKFPVREHHHHHH	C2
8TGG	MLRTILDAPQRLLKEGRASRQLVLVVVFVALLLDNMLFTVVVPIVPTFLYDMEFKEVNSSLHLGH AGSNCLQGTGFLEEEITRVGVLFASKAVMQLLVNPFVGPLTNRIGYHIPMFAGFVIMFLSTVMFA FSGTYTLLFVARTLQGIGSSFSSVAGLGMLASVYTDDHERGRAMGTALGGLALGLLVGAPFGS VMYEFVGKSAPFLILAFLALLDGALQLCILQPSKVSPESAKGTPLFMLLKDPYILVAAGSICFANM GVAILEPTLPIWMMQTMCSPKWQLGLAFLPASVSYLIGTNLFGVLANKMGRWLCSLIGMLVVGT SLLCVPLAHNIFGLIGPNAGLGLAIGMVDSSMMPIMGHLVDLRHTSVYGSVYAIADVAFCMGFAI GPSTGGAIVKAIGFPWLMVITGVINIVYAPLCYYLRSPPAKEEKLAILSQDCPMETRMYATQKPTK EFPLGEDSDEEPDHEE	C2
8TI8	SNASDKINVWTTSRDSAVCGDIELKKTSTTRLIFRPEIVNNNKNPKASVRGCFIFQKKGRNALW DDYKELDMNKLKAEEWIKLEINSDAMLTLTKEIQKHYAVHEKYGVRYGAFHLFKDNPDIEKLIEM FESNTDLLTQLMEDDKSEALEKTLEWIVTNDNPDKIIDRLKNLKEQDLDQLNTLIGIANLKKVLSV WESNKLTNTSEKFWQSVLKENTWILSQIFSNPTVLINDEAYVGGKTVKNDSGKLVDFLYANPFS KDAVLIEIKTPSTPLITPTEYRTGVYSAHKDLTGAVTQVLTYKTTLQREYQNIDYNNYRQGIKTDF DIITPCCVVIAGMFDTLTDTAHRHSFELYRKELKNVTVITFDELFERVKGLIKLLEG	D2
8TIA	MKSSHHHHHHENLYFQSNAKEQDLDQLNTLIGIANLKKVLSVWESNKLTNTSEKFWQSVLKEN TWILSQIFSNPTVLINDEAYVGGKTVKNDSGKLVDFLYANPFSKDAVLIAIKTPSTPLITPTEYRTG VYSAHKDLTGAVTQVLTYKTTLQREYQNIDYNNYRQGIKTDFDIITPCCVVIAGMFDTLTDTAHR HSFELYRKELKNVTVITFDELFERVKGLIKLLEG	D2
8TKA	MASSLRAAISKIKRDDVGQQVCPNYVMLRSSVTTKVVRNVVEYQIRTGGFFSCLAMLRPLQYA KRERLLGQRNLERISTRDILQTRDLHSLCMPTPDAPMSNHQASTMRELICSYFKVDHADGLKYI PMDERYSPSSLARLFTMGMAGLHITTEPSYKRVPIMHLAADLDCMTLALPYMITLDGDTVVPVA PTLSAEQLLDDGLKGLACMDISYGCEVDANSRPAGDQSMDSSRCINELYCEETAEAICVLKTCL	C2

	VLNCMQFKLEMDDLAHNAAELDKIQMMIPFSERVFRMASSFATIDAQCFRFCVMMKDKNLKID MRETTRLWTRSASDDSVATSSLSISLDRGRWVAADASDARLLVFPIRV	
8TS1	MVAGEVHRFRTSDVSQATLASVAPVFTVTKFDKQGNVTSFERKKTELYQELGLQARDLRFQHV MSITVRNNRIIMRMEYLKAVITPECLLILDYRNLNLEQWLFRELPSQLSGEGQLVTYPLPFEFRAI EALLQYWINTLQGKLSILQPLILETLDALVDPKHSSVDRSKLHILLQNGKSLSELETDIKIFKESILEI LDEEELLEELCVSKWSDPQVFEKSSAGIDHAEEMELLLENYYRLADDLSNAARELRVLIDDSQS IIFINLDSHRNVMMRLNLQLTMGTFSLSLFGLMGVAFGMNLESSLEEDHRIFWLITGIMFMGSGLI WRRLLSFLGRQLEAPLPPMMASLPKKTLLADRSMELKNSLRLDADLEDNWETLNDNLKVIEKA DNAAQVKDALTKMRAAALDAQKATPPKLEDKSPDSPEMKDFRHGFDILVGQIDDALKLANEGK VKEAQAAAEQLKTTRNAYIQKYLSNSLEVLFQ	C5
8TWQ	MKNGFYATYRSKNKGKDKRSINLSVFLNSLLADNHHLQVGSNYLYIHKIDGKTFLFTKTNDKSLV QKINRSKASVEDIKNSLADDESLGFPSFLFVEGDTIGFARTVFGPTTSDLTDFLIGKGMSLSSGE RVQIEPLMRGTTKDDVMHMHFIGRTTVKVEAKLPVFGDILKVLGATDIEGELFDSLDIVIKPKFKR DIKKVAKDIIFNPSPQFSDISLRAKDEAGDILTEHYLSEKGHLSAPLNKVTNAEIAEEMAYCYARM KSDILECFKRQVGKVKD	C2
8TWT	MGSSHHHHHHSSGLVPRGSHMMKAILQLILEKRQEFEKLPCFEFVRDETISPEERLILYPCIAAF ALNFRDLNRYDYRDDNSSDYYQKIINIHTQEDAKHWEWFLNDLELLGFDKTMRFSEALRFVWS DDLLHTRRLCHNIAVLSHDLEPVMKMVVIEAMETAGLVIFHALAKPGESIAKATRRKYLYVADSH VEVETGHAVGTENIITILEQTQLSSEQEEKAKEIVNKVFQWSTNLIGEFERYVKAHRSEKAQPTA AY	C2
8TX9	MDKPDIATIIDSHFEEMTDLEQEIARYFLQAETITDDLSSQQVTQKLHISQAALTRFAKKCGFTGY REFIFQYQHQAENQANQVSKHSPLTKRVLRSYSNMREQTQDLIDEIQLERIAQLIEDAERIYFFG TGSSGLVAREMKLRFMRLGVVCEALTDQDGFAWTTSIMDENCLVLGFSLSGSTPSILDSLLDAK EMGAKTVLFTSVPNKDSQTYTETVLVATHSQPSYIQRISAQLPMLFFIDLIYAYFLEINRESKEKIF NSYWENKKLNGYRRQKRVRKS	D2
8U8M	SDVEISDSFEKAMWNHVSQNAARLTGKFLMTEQFWAELLQSQPNIPIKSAHIVLHHFNEMMLE NLWKQAMDPDAKLQILKDLSVPLSYHQRKWILDNDRLDVALSLDGYVVSWDIVR	C2
8UGZ	SLYERLGGEQKIARIAADIFDTHATNPTVASRYKDSDRERVIKMVTEFLSAGTGGPQDYTGKSM PEAHRSMNINEAEYLAVIDDIMVALDKNEVGDQEKQELLMIAYSLKGEIIGA	D2
8UP1	SGTVTFDITNISHKAIDIILKVVLGIAEHEGTEVTFHSERGQLQIEVKNLHEEDKRLIEQAIEAARLA DSPDPESVARAVELLTKVAKASTNTELIQFIVKELLELARKLTDPKDLAKVLDSISELLTELALKTG DPTAALAAMVAHIAELVVRLALMAERTHPGSEIVKKAVKLVQEVAEEVLEAAQLMLEKPNSDEVA KKLEEVAKKAIEACIELQQILEAWAKERGDQDLLREVREHKLQILTIAVAYKAAQMGVTVLKHTHG WVVFLVILGLHKQQAEQLLRFVHRVAHALGVTLSITFSGDIVVIAVTVGASEEEKKEVRKIVKEIA KQLRHAETEEEAKEIVQRVIEEWQEEGGSG	C4
8UPB	ANLYFQSDREEFQWLVEEFIRVLERGDVEKAREILRLLKEVAEKVNDPLLRLLFRIARRLVEEL	C2
8UTK	MPLWQVFYLLNTCIKRTGDPTCKKLAKALRECLKKGDLKACNELADKAVKYINSLE	C2
8UVT	GPGRAPPSPDLRADEPKTPCLVGGAHAFILKISSFCGLAPLRFEPRSQEYAVTISKGKCFYSYIL VTFLVICTIYGLVAEIGVGVEKSVRMSSRMSQVVSACDILVVAVTAGVGVYGAPARMRTMLSYM ENIVAVDRELGRHHSAATERKLCALLLLILLSFTILLVDDFCFYAMQAGKTGRQWEIVTNYAGFYF LWYIVMVLELQFAFTALSLRARLKLFNEALNVTASQVCKPVKKPKNSQLSVYATSVRPVSCKRE NVIVETIRVRDKDDAFVMMKTADGVPCLQVPPCEAVGRLSRMRCTLCEVTRHIADGYGLPLVIIL	C4

	MSTLLHLIVTPYFLIMEIIVSTHRLHFLVLQFLWCTTHLIRMLVVVEPCHYTIREGKRTEDILCRLMT<br>LAPHGGVLSSRLEVLSRLLMLQNISYSPLGMCTLDRPLMVTVLGAVTTYLVILIQFQRYDS	
8V6Q	MHHHHHHSSGVDLGTENLYFQSNASNNLSEINLDVEGSIVTVKAGDLFRQDGFKVIAFNEYFDT<br>QVDDVIISHNSLNGLYIDNYLAGSVSDLDHRISNHQFEEDELLEVNHKRKVGKTQKYSLGTIFVN<br>NDYLLTAFSKFDDKNRAFLTMPDYLAFLINFWDKVNRIYAQKSVSVPIFGSGITRIKEHKNISDED<br>LLKIMLWTFRISEMRFKFPAKLTIVIHKDKIDKINLLDIKSARNGL	C2
8V7W	SGTSAVLQSGFRKMAFPSGKVEGCMVQVTCGTTTLNGLWLDDVVYCPRHVICTSEDMLNPNY<br>EDLLIRKSNHNFLVQAGNVQLRVIGHSMQNCVLKLKVDTANPKTPKYKFVRIQPGQTFSVLACY<br>NGSPSGVYQCAMRPNFTIKGSFLNGSAGSVGFNIDYDCVSFCYMHHMELPTGVHAGTDLEGN<br>FYGPFVDRQTAQAAGTDTTLEHHHHHH	C2
8VJN	MHHHHHHMAKNSNPSAFDRDFGYLMPFLDRVAAAASDLEDASARAELTRLMVEEKARWQRIQ<br>ELLG	C2
8VKT	MGSSHHHHHHSSGLVPRGSHMASMKKEEKIAILQEIIRIKSVNGNEGEVAAYLNKLLARHDITGE<br>IVSYRDGRDNLIARYQKGQSGKVLGLSGHMDVVAAGDESSWTYAPFAAEIHGNRLYGRGATD<br>MKSGLAAMVIAMIELKESGKPFNGTVKLLATVGEEVGELGGEQLTKAGYVDDLDALIIGEPTNYS<br>LMYTHMGSINYTVTSHGKEAHSSMPDQGYNAINHLNEFITKANAEMNHLAETIENPVLGKTIHN<br>VTLISGGNQVNSIPSHAQLQGNIRSIPEYPNDKIIALLQSIVNELNQETDYHLELMIDYNKIPVKAD<br>PDSPLIHSIQQQFSQPLPLVGAAATTDAAEFTKANHSFDFVVFGPGVVTLPHQVDEYVEIDNYL<br>DMIEKYQGIILSYLA	C2
8VVA	MSKTNVRIGAFEIDDAELHGEHQGERTLSIPCKSDPDLCMQLDAWDADTSVPAILNGEHSVLYR<br>KHYDRQSDAWVMRLA	C2
8W0K	SRPQSTLRRAITAAYRRPETECLPPLVEAATQSKEIRDAAASTARKLIEALRGKHSGSGSSGSM<br>MGEQFVTGETIREALKRSKELEEKGFSYSYDMLGEAATTAADAERYYRDYESAIHAIGKASAGR<br>GIYEGPGISIKLSALHPRYSRAQAARVMGELLPRVKALALLAKNYDIGLNIDAEEADRLELSLDLL<br>EVLCLDGDLSGWNGMGFVVQAYGKRCPFVLDFIIDLARRSGRRIMVRLVKGAYWDAEIKRAQL<br>DGLADFPVFTRKIHTDVSYIACAAKLLAATDVVFPQFATHNAQTLAAIYHMAGKDFHVGKYEFQ<br>CLHGMGEPLYEEVVGRGKLDRPCRIYAPVGTHETLLAYLVRRLLENGANSSFVHRINDPKVSID<br>ELIADPVEVVR	C2
8W33	MHHHHHHGKPIPNPLLGLDSTENLYFQGIDPFTMSTDKFEPVPLPEILIFPNRLLSAETTEKLLNR<br>VYDVPHVRQVNISGEGVPAMVGSGPGKGLPVEHEGRKVINVKGREIELQLLVGRVFVEIDDIDV<br>VEKAIEAIDEICQELLPFGYNLEVGRYSKYRPTVTDYKKGKR	C2
8WAC	MGQSITWNNKQTDIQPGETIPLNITYDAGVGNTVYYVSVVLQEMNASWQTQNNYNTTYPVSG<br>SNQPNASTIDFNYTIDSNIPLSENLPSGNFYLLKIFISVNTDGAFANDNTQITLLNNLEHHHHHH	C1
8WCM	HHHHHHGSGGDYFPVISVDLQSGRRVVSVEYIRGDGPPRIPYSMVGPCCVFLMHHRPSHEVR<br>LRFSDFYNVGEFPYRVGLGDFASNVAPPPAKPFQRLIDLIGHMTLSDFTRFPNLKEAISWPLGE<br>PSLAFFDLSSTRVHRNDDIRRDQIATLAMRSCKITNDLEDSFVGLHRMIVTEAILRGIDLCLLPGF<br>DLMYEVAHVQCVRLLQAAKEDISNAVVPNSALIALMEESLMLRSSLPSMMGRNNWIPVVPPIPD<br>VEMESEEESDDDGFVEVD	C2
8WCN	MGSDYDIPTTENLYFQGSMLARDSLVQAGLPDNPYARQLRNGFRWLRFEKELENEFREFLSW<br>NSLMQRRAAIGVAFLIWALFIVADWMMVDIRLHPSLFEQLLGVRLGMIGLLLVVWPAAFLPSLRK<br>VGDAIAPYCLLLINLAVLACDVLFEWHGVPRFTQLGATLGILAVFFPLGLAFWACVRLALLCLALN<br>LAVFLLFGGEENLRTNLLNTLYNGLVVLICSFALYLQDYAQREQFLGRRLLGMMAEQDSLTGLVN<br>RRYYELLAQRALEQGAREEKGVALILVDVDDFKAYNDHYGHPAGDAALRQLGVVLRQGARRPL	C2

	DIAARLGGEEFAVLLYDSEEGNTLAIAERLRQAVEALGIEHLGSSAGPCLTISLGVAYSTSGMGLD<br>ALYREADRALYEAKDAGRNAVRVAFRQHDRLEGSFLSAWSHPQFEKGGGSGGGSGGGSWS<br>HPQFEKLEHHHHHH	
8WEX	MGHHHHHHMSNARIGIDMSKIYAPKSIDLSSFQLPDEKLQTKYGLPHKVEFCKSCVISNQRPNS<br>AVEYEHKKESKKHTIHFDDEGICDACRVAERKKSTINWEERDRQLRELCDRFRSKDGSYDCVV<br>PGSGGKDSFYAAHILKYKYGMNPLTVTWAPHMYTPWGWRNFQSWIHAGFDNHLFTPNGRVH<br>RLLTRLAVENLFHPFQPFMIGQKAYAPKMALLHKIKLVVYGENEAEYGNPIGDTESAKRDWKYF<br>TADDKSKIFLGGTSVQELKSDFGLNDNDLDAYLPADPQQIEEQQVEVHYLGYYLKWHPQSCYY<br>YSVEHGGFEASPERTPGTYSKYNSIDDKIDDFHYYTTLTKFGIGRATYDASQEIRSGDITREEGV<br>ALVKRFDQEFPERFAEEIFKYLSINLKEFPIASQMFEQPIMDRAYFMALADTFRSPHLWKKDGEQ<br>WKLRHQVTNLEKTKAEYLDLETV	C2
8WQ8	MFEKLYSAIIYSDEFKKILLGRGVDDLEIASAYIAFLYEDLPIIGKNLCAAFLRMGLDAVYNVMPSG<br>KVYSPRHKLYPISRYGIDGVCINCDGGKIILRISNKGYDPEDLLESKGLESRIFVSKNFKKKSMEII<br>EKIWDVNKIRLIARKEILERISAGGILHMIRLEHHHHHH	C2
8X39	MSVKIGDIDGNGEISSIDYAILKSHLINSNLTFKQLAAADVDGNGYVNSIDLAILQMYLLGKGGTS<br>DIGKNRIYTYGDIDNNGIVDENDYILICNHINGTGQLSDASLFAADADGNNVIDQTDRILIEKYITG<br>RITHLPVGNQLEHHHHHH	C2
8X5B	APAWTTFRVGLFCGIFIVLNITLVLAAVFKLETDRSIWPLIRIYRGGFLLIEFLFLLGINTYGWRQAG<br>VNHVLIFELNPRSNLSHQHLFEIAGFLGILWCLSLLACFFAPISVIPTYVYPLALYGFMVFFLINPTK<br>TFYYKSRFWLLKLLFRVFTAPFHKVGFADFWLADQLNSLSVILMDLEYMICFYSLELKWDESKG<br>LLPNNSEESGICHKYTYGVRAIVQCIPAWLRFIQCLRRYRDTKRAFPHLVNAGKYSTTFFMVTFA<br>ALYSTHKERGHSDTMVFFYLWIVFYIISSCYTLIWDLKMDWGLFDKNAGENTFLREEIVYPQKAY<br>YYCAIIEDVILRFAWTIQISITSTTLLPHSGDIIATVFAPLEVFRRFVWNFFRLENEHLNNCGEFRAV<br>RDISVAPLNADDQTLLEQMMDQDDGVRNRQKNRSWKYNQSISLRRPRLASQSKARDTKVLIE<br>DTDDEANT	C2
8XGL	ADPHHHHHHYINSMSAPASVQRGQAFTAQLNSSIYVQNYDDFGVVWGLAPPNLNTSACVGCV<br>GRRIGYTNLFGDKADVQVPPSGTVGVQVTVPADQAPGEYLLIAGASYLVGASGVTGFNYFNTT<br>VQVCE	C2
8XH6	SIGLALLLLLLALLFWLYIVMSNWTGGALLVLYSFALMLIIIILIIFIFRRDLLCPLGGLGLLLLMITLLLI<br>ALWNLHGQALYLGIVLFIFGCLLVLGLWIYFLEILWRLGATIWQLLAFILAFFLAIILLIIALYLQQNW<br>WTLLVDLLWLLLFMAILIWMY	C2
8XTE	GAMGAASPASIIQELASAAKQYENNESGAREALIAQSRALIASLEVPSEFIQHTFWSQPALSAIV<br>RLATDVNLFQYLKDAQEEGLNAEALASKTGMDVSLFARLARHLVAMNVITSRNGVFYGTALSNG<br>LAAENYQQSIRFCHDVSRPSFGAFPSFFKGNGYKTPALGTTDGPFQSAHKVDISFPQWLVGNP<br>PYLQYFNSYMSAYRAGKPNWCDNGFYPVADRLLNGFDASVSDVLLVDVGGGRGHDIATFGSQ<br>FSPLPGRLVLQDREQVINSIPADESRQFEATTHDIFTTQPVKHARAYYMHSVPHGFGDEDAVKI<br>MANLVPALAKGYSRVLLNEIVVDEERPVMSATNMDLIMLAHMGAKERTEADWRSILTRAGLKVV<br>NIYSYPGVAESLIEAELA	C2
8Y75	MKKILIVSFLGKGRYYETFYYSIEHSEKMVKKRLSPLANAILEKENGNDVEIIFFVTNEVKNEFLY<br>DENNEYAKNILNELNEIKNYGIKVSYRDIPKGKNYEELEIIMEEIEKLLLDFKGNKVIFDLTHGLRH<br>MAIFTSSTVFYFKNLMEKANKLEMKIVYGAYEIGEEIEKNLKKVPILDITQTLELSDLTIALEEFERY<br>GITERMIIVLKNIQKIVAKNKLCNLNELKFSSLSRELKLFEELLKIPSPPEKIANSIYKINDILESSIRE<br>FKLCSKNSENLFFIKPIQKFLVDFQKIVLEKLPLDKKINKYSNIATLEKVEFMKNIIKLLINWKMYSE<br>AVIHLRELLIDIKLIENGKYFYYNNKDFREKYWMYSYNIVDTKDKELPKKIEELLKNVKGWRNSVA<br>HGGRANTSINQKTLEENLENALSMIDEILLSMKDLKVNS	C2

8Y7F	ITQTLELSDLTIALEEFERYGITERMIIVLKNIQKIVAKNKLCNLNELKFSSLSRELKLFEELLKIPSP<br>PEKIANSIYKINDILESSIREFKLCSKNSENLFFIKPIQKFLVDFQKIVLEKLPLDKKINKYSNIATLEK<br>VEFMKNIIKLLINWKMYSEAVIHLRELLIDIKLIENGKYFYYNNKDFREKYWMYSYNIVDTKDKELP<br>KKIEELLKNVKGWRNSVAHGGRANTSINQKTLEENLENALSMIDEILLSMKDLKVNSKKIYLLNS<br>TIMPIPKDNQEGKFYILKLTKNEFKVILENAIKDDVLDSAIGHESVIEFIKDKFELTVPLKRKEIYFEK<br>GESALVIKLEKRPEEGKIYTKEEMDFMEENNLIGYYYIYREG	D2
8YEK	MKTIIALSYIFCLVFADYKDDDDAMGVASSAVITANWISFLAISASFIILLVISLRYKGPGGTESFYN<br>GFKEQNMLTVFINLWCALAYFAKVLQSHSNDNGFAPLTVIPYVDYCTTCPLLTLDLLWCLDAPYK<br>ISSAVLVFTCLVIAVACSLAVAPFSYCWFAMGMVLFTFTYVFILSIVRQRLDFFTLCARDSNAKQS<br>LKHLKTAVFIYFGIWLLFPLLWLLSYRAANVISNDINHIFHCILDVIAKSVYGFALLYFKMYFDKKLIE<br>SGIDEDDFAKFSKVVTTHRSEEKQKRKPAVSQSPKMYYDEAAYQDGEVESNLQSKIRKSLSRK<br>DKTPGSPSRSPMTPRTAYSTRKSPGMHLNDHPAWGSLQASAHSWENEREGPVGDHDDEEFS<br>EFCQSLPKKAIPPLNSQPHMYNSEDEDDGAYLERAKMAYQKAKERHDNANGQRERRDRSGS<br>RESDFENLYFQ	C3
8YEL	MKTIIALSYIFCLVFADYKDDDDAMGTTSAPSLSDPNWQYGMGGWNNPRLPNFNLHDPTVIGV<br>DWLGFLCLLGASLALMYKLMSFKGPDGDQEFFVGYREEKCLSIYVNLIAAITYWGRICAHFNND<br>MGLSLSVNYFKYLDYIFTCPILTLDLLWSLNLPYKITYSLFVGLTIACGVFCNAFEPPARYLWFMF<br>GCFIFAFTWISIIRLVYARFQQFLNEDAKKIRAPLKLSLTLYFSIWCGYPALWLLTEFGAISQLAAHV<br>TTVIMDVAAKSVYGFALLKFQLGVDKRDVWLDELKSVRYRDVVPQIRPSKTREGRMEYSEDGD<br>FMRPSKGKRAEGDYMNPRWDHHDDGRRLPDSREMDEQVHEKDQEISSTMKQIADLNKQLSA<br>MQESEAVENLYFQ	C3
8YLA	MEVWEHSRPIADDTIKKTPSFTTLPIRINKQNDVADAATRRALRDWDYYLHDGLAERALISISEL<br>GNLGAFAYPEVPPERLAIVTYLTDLGILHDDGYEAMDMDQARTEHREFGALFDPHEQLPSRRG<br>TRAAKLKKLVSQILLEAIRIDRDMGMYMFDMYNKGWLSVAGGEGKVPQFKSVEEYQAYRRDDF<br>GIRAFWPMVEFGMAMRLSDEDKKLIEPVMEPIDKAIIWTNDYWSFDREYHESITNGSRLTNVVE<br>VVRQIENKSIDEAKAAVRQLLVNLEQQYLERKRAIYAQNPSIPSHLRKWIEVVGITVAGTHFWAS<br>CSPRHHAWRNNSRNGLKPA	C2
8YLZ	HHHHHHMFHRAVAPATVPAQTPPAPHTASEGMTSMQHARTAGATGREPPPPLLPPPPLLPPPL<br>PAGTTPAPDRAPGGAHPGNPRHVGVILDGNRRWAEGHGTTLEAAYARGAARVADLVSWCETE<br>GIAVVTVWALSRDNLRRDAAAVGRILDATAVGLAGIAASGRWHIRLIGETDLLPAAPARRLRSVA<br>DRTATGSPGTLNVAVAYDGRADIAGAVRGLLRSGVTGGTAAAVRESDVERYLSTAGLPDVDLVI<br>RTSGERRLSGFLPWQTAYAELHFTAALWPAFSFDDFTVALRSYRARRRRFGF	C2
8YM1	GPSRATPARKQMDKPEWKRVPNSEEDVRKCFGPRSVSRNFGDSDLVQHGVEAKHFPTIAELL<br>PTQAALAFGSEITTKESGEFVEVTYHYVMKVPKTDKNLPRFLEQVSAYSK	C2
8YMK	MENITSGFLGPLLVLQAGFFLLTRILTIPQSLDSWWTSLNFLGGTTVCLGQNSQSPISNHSPTSC<br>PPTCPGYRWMCLRRFIIFLFILLLCLIFLLVLLDYQGMLPVCPLIPGSSTTSTGPCRTCTTPAQGT<br>SMYPSCCCTKPSDGNCTCIPIPSSWAFGKFLWEWASARFSWLSLLVPFVQWFVGLSPTVWLS<br>VIWMMWYWGPSLYSILSPFLPLLPIFFCLWVYI	C2
8YPJ	MTKEKISVTVDAAVLAAIDADARAAGLNRSEMIEQALRNEHLRVALRDYTAKTVPALDIDAYAQR<br>VYQANRAAGS	C2
8Z1E	SADRAASDLLIGMFGSVSLVNLLTIIGCLWVLRVTRPPVSVMIFTWNLVLSQFFSILATMLSKGIML<br>RGALNLSLCRLVLFVDDVGLYSTALFFLFLILDRLSAISYGRDLWHHETRENAGVALYAVAFAWVL<br>SIVAAVPTAATGSLDYRWLGCQIPIQYAAVDLTIKMWFLLGAPMIAVLANVVELAYSDRRDHVWS	C3

	YVGRVCTFYVTCLMLFVPYYCFRVLRGVLQPASAAGTGFGIMDYVELATRTLLTMRLGILPLFIIA<br>FFSREPTKDLDDSFDYLVERC	
8ZAO	PFYDSRPPEGWPKGSINDMDYPLLGSICAVCCVFVAGSGIWMLYRLDLGMGYSCKPYKSGRA<br>PEVNSLSGIICLLCGTMYAAKSFDFFDGGGTPFSLNWYWYLDYVFTCPLLILDFAFTLDLPHKIR<br>YFFAVFLTLWCGVAAFVTPSAYRFAYYALGCCWFTPFALSLMRHVKERYLVYPPKCQRWLFWA<br>CVIFFGFWPMFPILFIFSWLGTGHISQQAFYIIHAFLDLTCKSIFGILMTVFRLELEEHTEVQGLPL<br>NE	C3
8ZLH	MMFGSTKPESGDSKWRSQLDRFVKENQQDLAALFWGLWLENGDSQGTIGIDLQPTPHFVYC<br>PKDAVEKLNNNVENRLQELLGIIEHNQPEIEVLMIGIGKGEIKLIQFAPEPPPPVCFEQVGKDIDG<br>LLELLEQRMSGEIVV	C2
9ARD	SQFNFECFVIEDNKEVLYNSVSRFLPKKRRLTFKLSIYPGPGIGDLKIIFCKRNHGQEAKDDLSE<br>DYSISIEDNKLIRVKNADNLSLLRKDGCYVLTVPEETLFRGLHTMEVIVRGNHETLFYRNIIGVYIK	C2
9AZO	MSKTIKVALAGAGAFGIKHLDGIKNIDGVEVVSLVGRRFDQTKEVADKYGIAHVATDLAESLALPE<br>VDAVILCTPTQMHAEQAIACMKAGKHVQVEIPLADALKDAQEVAELQKQTGLVAMVGHTRRFN<br>PSHQWVHKKIEAGEFNIQQMDVQTYFFRRTNMNALGQARSWTDHLLWHHAAHTVDLFAYQA<br>GSPIVKANAVQGPIHKDLGIAMDMSIQLKAANGAICTLSLSFNNDGPLGTFFRYIGDTGTYLARY<br>DDLYTGKDEKIDVSQVDVSMNGIELQDREFFAAIREGREPNSSVQQVFNCYKVLHDLEQQLNA<br>D	C2
9B4E	MASCVGSRTLSKDDVNYKMHFRMINEQQVEDITIDFFYRPHTITLLSFTIVSLMYFAFTRDDSVP<br>EDNIWRGILSVIFFFLIISVLAFPNGPFTRPHPALWRMVFGLSVLYFLFLVFLLFLNFEQVKSLMY<br>WLDPNLRYATREADVMEYAVNCHVITWERIISHFDIFAFGHFWGWAMKALLIRSYGLCWTISITW<br>ELTELFFMHLLPNFAECWWDQVILDILLCNGGGIWLGMVVCRFLEMRTYHWASFKDIHTTTGKI<br>KRAVLQFTPASWTYVRWFDPKSSFQRVAGVYLFMIIWQLTELNTFFLKHIFVFQASHPLSWGRIL<br>FIGGITAPTVRQYYAYLTDTQCKRVGTQCWVFGVIGFLEAIVCIKFGQDLFSKTQILYVVLWLLCV<br>AFTTFLCLYGMIWYAEHY	C2
9BE2	GSHMSLDINQIALHQLIKRDEQNLELVLRDSLLEPTETVVEMVAELHRVYSAKNKAYGLFSEESE<br>LAQTLRLQRQGEEDFLAFSRAATGRLRDELAKYPFADGGFVLFCHYRYLAVEYLLVAVLSNLSS<br>MRVNENLDINPTHYLDINHADIVARIDLTEWETNPESTRYLTFLKGRVGRKVADFFMDFLGASEG<br>LNAKAQNRGLLQAVDDFTAEAQLDKAERQNVRQQVYSYCNEQLQAGEEIELKSLSKELAGVSE<br>VSFTEFAAEKGYELEESFPADRSTLRQLTKFAGSGGGLTINFDAMLLGERIFWDPATDTLTIKGT<br>PPNLRDQLQRRTSGGN	C2
9C9Q	MTDPNSPYFEKVLGSLFARQVEPAKDYAWDMGSTLPTPDDLMRRFIVKDTLITIFRRHGAVEAP<br>TATLYPKSSHYGPNAVHLLDRNGTVLQLPFDLVMGHARSLARIASGPVPQRAYSFGNIFRDRQD<br>GGQPDVYGEVDFDIVTTDAMDLAMKEAEVIKVLDEIIAAFPTTSSTPMCFQLGHSDLLQLIFDFC<br>NVEHGARQAAAEVLSKLNIRNFTWQKVRSELRSPLVGISATSVDELQRFDWRDTPTKAFTKIRN<br>LFEGTEYYDKVSSTLAHLKEVYEYSKKFKVNTKIYIAPLSSINEAFFRGGILFSCLYDRKVMDVFA<br>AGGRYDGLIKAHRPRIGSRFEERHAVGFSLNWEKQLAKPVPKTTGKAFLKKAAEEEAQGIFSA<br>KRCDVLVASFDPSILRSSGIELLQMLWAHGISAELARDARSPEDLLTTYRDESYSWIVIIKQESQL<br>KIKTMHRKDVPDADIQAKDLLAWLKAEI	C2
9EMU	WSHPQFEKIEGRMSDGRESFLEVMRSVYERYLVGVPGVSEVWLIRHADSYTGLEDYDGDPRD<br>PALSEKGRAQARLLAARLAGVPLHGVWASGAHRAQQTASAVAAEHGLRVRTDARLREVRTNW<br>DDGRPSELKPHGVYPFPEPEKEVAERMRTAVTAAVAATPPAPDGTTRVAVVGHDSALVILMGSL<br>MNLGWGQLDMILPLTSVSVLAVKDERMVVRSIGDATHLAAAPSDVI	C2

9F9Q	AMADNDTDRNQTEKLLKRVRELEQEVQRLKKEQAKNKEDSNIRENSAGAGKTKRAFDFSAHG RRHVALRIAYMGWGYQGFASQENTNNTIEEKLFEALTKTRLVESRQTSNYHRCGRTAKGVSAF GQVISLDLRSQFPRGRDSEDFNVKEEANAAAEEIRYTHILNRVLPPDIRILAWAPVEPSFSARFS CLERTYRYFFPRADLDIVTMDYAAQKYVGTHDFRNLCKMDVANGVINFQRTILSAQVQLVGQSP GEGRWQEPFQLCQFEVTGQAFLYHQVRCMMAILFLIGQGMEKPEIIDELLNIEKNPQKPQYSM AVEFPLVLYDCKFENVKWIYDQEAQEFNITHLQQLWANHAVKTHMLYSMLQGLDTVPVPCGIGP KMDGMTEWGNVKPSVIKQTSAFVEGVKMRTYKPLMDRPKCQGLESRIQHFVRRGRIEHPHLF HEEETKAKRDCNDTLEEENTNLETPTKRVCVDTEIKSII	C2
9FEK	GPMAKKRTYQGKVPLHDNYGPEAKYAVEAEALLPTTKFEEEIARGLELGLPGADSIKDRRIPTF SRGELPHFAGINTFIKAPYVEDVRKCGQYDVAILGAPFDGGTTYRAGTRFGPQGIRKISALYGTY SFELGVDLRESVSICDVGDIFTIPGNIEKTFDQVSKGVGHVFASGAFPVVLGGDHSLGFATVRG VAQHLNGKKLGILHFDRHVDTQDTDLDERMHTTPWFHATNIPNVPAKNLVQIGIGGWQAPRPG VKAGRERQTTIMTVTDCVEMGIENAAKQALEVAFDGVDAVWLSFDVDCLDAAFVPGTGWPEP GGFLPREVLKFLQIIADTKPLAGMEIVECAPPYDAAEITSLMATRVICDVLACQVRSGHLGNRKK R	D3
9FS9	MASAVLVTGEVSNVDLDKTTITISEDGKTFNYNYEEAIFKLHNNVVSQSKFESLLFGATVTASKD DKGVLTLNIIDEGVDALEHHHHHH	C1
9FSA	MVEKIGDVEGFKVIDNGEPTADIVVGSTAAAADVVSAANVAAKVGSMMFKEGEGGSDAKAPVA FKAPLAVLDTEVSLDAANKKLILVGGPVANALTKELADAGKIEMTVESPATLAVVAGAANGNDVL VVAGGDRAATAEAANALIEMLLEHHHHHH	C2
9G2U	MNIMDFNVKKLAADAGTFLSRAVQFTEEKLGQAEKTELDAHLENLLSKAECTKIWTEKIMKQTE VLLQPNPNARIEEFVYEKLDRKAPSRINNPELLGQYMIDAGTEFGPGTAYGNALIKCGETQKRIG TADRELIQTSALNFLTPLRNFIEGDYKTIAKERKLLQNKRLDLDAAKTRLKKAKAAETRNSSEQEL RITQSEFDRQAEITRLLLEGISSTHAHHLRCLNDFVEAQMTYYAQCYQYMLDLQKQLGSFPSNY LSNNNQTSVTPVPSVLPNAIGSSAMASTSGLVITSPSNLSDLKECSGSRKARVLYDYDAANSTE LSLLADEVITVFSVVGMDSDWLMGERGNQKGKVPITYLELLN	C2
9ILV	MDHRTSIAQAMVDRISKQMDGSQPDEYFNNLYGNVSRQTYKFEEIREFPYVAVHIGTETGQYL PSGQQWMFLELPILVYDKEKTDIQEQLEKLVADIKTVIDTGGNLEYTVSKPNGSTFPCEATDMIIT SVSTDEGLLAPYGLAEINVTVRYQPPRRSLRR	C6
9INK	GPHMGVQTCNASSPDFQLCVRASLQQLIPELASGVPSIGAEGVDPLRGLPPIVHNSNGFKVQL DDVSISGLSATLINDVNVDLTSNTIRIQATVPGYITATGIQTTDAEIMGIPLKGSGPFTISLANPSLA VTLTGAPSAGPNGQTYLRLTSASAAIEPGTPTADIKGFFPQFPPLEAAASAFASVVAPDVVQSLK PTLDKWLGGVALQRAQAVFSSVSYDALFPGRTPAAVGLYRAVPGLHTLPLPLSAFAYHK	C2

Supplementary Table 15. Protein sequences from the Denovo test set

Source publication for the protein structures:

HALxxx, PMID: 36108048

HExxx, PMID: 37433327

ID	Protomer sequence	Protomer length	Symm-etry
HALC1_006	DKIAFFKRLKEELEKDPSDENVEKLIETLNEEEKKILEEIKKEYPNEPLSEIFYKLIEKLLELSE	65	C1
HALC2_067	DKLVRVLSSSMIYYAERMTKGSTDPSDYDKALDDFYNYFLEQPFVDKETLEKAYELARKRLEELL	65	C2
HALC3_104	KRIDEIESKLKHLEEFTTHLIKLMETMLELLKLVSDGKSDSEEYKELLEKAEEYLKQATEAAKKI	65	C3
HALC3_110	LEQILEELTELLERVDEIPLREALKRMLELLVRVTQELKEVKDKVESLEKHLEELDKRVEEIEKK	65	C3
HALC5_169	LLLEVMEKVFDEEQLKLIKEAAEREGNSPVVISSIATLLLLERIEKIVKEIHDEVKKNNEKQEKK	65	C5
HALC2_062	MARVEYSYEKLNDTHYKLKLKVTYEYRKSPEARRLAEDLVQAFVDALSSLPFITVEYEVEEVEVE	65	C2
HALC5_176	MDPKELEREALKNIIKLPKLIQDFKDSVMKELNKIIELLEERRREIDEPLLPIIRKLQEELQKKE	65	C5
HALC4_140	MEEVVLTSHNELHKKLDEVHDKIMSKLDEIHEKLDEIISKLDEIESKLHEILNIVKEIKEILEKK	65	C4
HALC4_135	MEKFKEQLLEEVKKIVLETMTKVMEHLEKWFVTLAEIIITKSEEKLEELKETMEKSIEELRKEAE	65	C4
HALC2_068	MIKVPEDLERIGRELRARGLDTKRLLEEGPKLYPELSIPDLMAIALYDHLNLDPEFLYRLLQQSR	65	C2
HALC1_004	MIVSLEKHPGGVHIITLSSEENLENFVKELKKLGAEVERLPEPNTVRVRAPEEVVEEALKNTKFK	65	C1
HALC2_063	MKVYEFPYPETGKKIIVIQGEKNIVIVVGNTAVVYYEGKWTYKENVTEEDIEKAKTEEGAKELAK	65	C2
HALC1_008	MLTPEELLERLRRHLEEEHGVVVPEERILSVEATPTEVTLTWSRGDGRTGTARYTSDGRFEVEDP	65	C1
HALC4_136	MSPYKKAIEITKRLLELLLSNPELAKKNLGGIATLISLLALISALDGTLDEKDIEPYIKKLEESL	65	C4
HALC3_118	MTRLEQLLAQGVDPFEVLREKIEKLKEIWKKYEEAKGEEKERYRDELLKLMMEVLELMVELLSRR	65	C3
HALC2_059	MVKPITEEDVREAATAASPDYEVGEAKLIDEENNLWFVTLYKGDQKIYALIEDKNGEFTVHQIEL	65	C2
HALC1_005	NEKEFLLQLKEELDKDPSEENVLSLIKTLNEEQKKILEEIKKKYPNLPLSKIFELLIDELLERLE	65	C1
HALC6_220	PPIPPPSFKLEISPAFLELVQLVIDLHPNDEEVRKELIENLISRIGKSDNVPPETISLDISEAALELFEWIFEKFPDDEDVHRRLIESFINKRKFSSSSPLDTPSLDISERFIELVKYILEKYPEDEEIKQKLIDSLLNLLGSY	144	C6
HALC3_109	REEIEEAVKEAELKVLAIVLVALRSVSHYEPLSRLYESFLDALKKALSEEELKEVEKEAERIEKK	65	C3
HALC2_065	SEEEKPIVIDLNKTIERDGRKVKLVRATITVDPETNTITIDIEYEGGPITKEDLLEAFKLAASKL	65	C2
HALC2_064	SKLKEQEELIDEISEKAKEFLLEIKEKYPGELSEERYPGRVVLTYVNEEKGFSITVTIELLNKEK	65	C2
HALC3_114	VDEKEVKERFEEIESRLEELESKVREVEKKVEEVKKESDEKIDQLKTEFETKYNQINNEINTLKN	65	C3
HE0368	SEEEDRKLLKEILAEPEVLELLLEAVKVVEPVYEQFLKALELQNEAWSLILKKFAEKKAK	60	D2

HE0370	DFKEKLKKLQEEMDKLLKKVEETLKEIAKEEENSMIYLAALTTLILAHQAKLNEKLEKANLELLKKEGHKELAKFIEELI	80	D2
HE0374	NEEVKKLLEELKKVSLEKTKEICKLLGLSEEVTELVLKSIEAQNESLEAFIEALEALKKA	60	D2
HE0376	MEKKEKLKKELERELALCETEADKLFILAAVNLMLALLELGREETAKRILKLMEKILELN	60	D2
HE0377	SAALEEAKALLEELAELGLPLSVALLLVALAEELRRLGLPAERAREFVLEVARRIVELLR	60	D2
HE0378	NNSRLENLLLLAKTYVEAALALLRGESPVPHFERAAAELEARGDEESAALVRAAAARERA	60	D2
HE0381	MVKELVDVIIEAQFDLSLRIAKVLGLPEEEAKAIRKRVEEKGDYVEAIAELNQKILKLLE	60	D2
HE0384	MEEKLEKALKFSEEVAEKVKELFKDEPLEKKVLALLVVYNAAKLLLAILSAEDPEKMKKLLKEINEREEKDIEELIKEDK	80	D2
HE0385	MVEKVEEIRKKLLELIEELAKEFLKDPELLELYEKLLKKVEEMRKIIEEKPEYKPLALLLEIQMSTLIFCTILLMAMMME	80	D2
HE0386	SLEEKLAALIAATEKKVAATEEVMKTFGFPESLIEELRAKLAELHTLDPEGQFIQIIEIQGLLNYLEQQALAELYKLLLK	80	D2
HE0389	EVEERLREVEELVDRYRERLLEAIDGPLEEIIALEVDFLREFLELGLPEKALLAAFNILNLLVNIEALMKDEPKEVRKEVREKILDLLEEILKLMIRAEA	100	D2
HE0390	MSKEEKMMEEMMKLCKEMKEKEGLKLSTMNLMMNIVFAYLEGEKVEEIRKACEKTLEMLK	60	D2
HE0391	MRTITVAPEYQEIWDAVDALMKKKGISEMDGLKMFGLMVALIDIYPESHEPIIEMLKSLI	60	D2
HE0392	MLESAKELLELLKKAGDELGLEIAKEYLKLMEKYKEYAPLIANNMLNALMNLCLLEGREELMVEVIEKIAKLMKEILKLK	80	D2
HE0393	MLESAKELLELLEKAGDELGLEIAKEYLKLMEKYKEYAPLIANNMLNALMNLLLKEGRLELAVEVIEKIAKIMKEILKKL	80	D2
HE0396	MEEEIRELTKEALTKLAELWKLALIALGVDKEAVDAAVAELKALLEESKDLPPLEFTKRIVEKCTAITKRFLDQAPLESLLFLAAVNLLLALDLLALQAL	100	D2
HE0397	MKEELKKKLKELLEEFEKLPLETQKLFAESLRVQAKLAGYEKLAKILEELIKLLEEMIKK	60	D2
HE0399	DADIEIDALLLKANLILRKLPLEKQAAFLKAVEALLKEMGLPQEAIDKVKEAAKILEAAI	60	D2
HE0400	SSALEELKEELVRLVEMSVEEILERAEELLKKAKKEGKLLELAKALLTLSLALLKLLLES	60	D2
HE0401	SSALEELKEELLRLVEMSVEEILERAEELLKKAKKEGKLLELAKANLTLNLALLKLLLES	60	D2
HE0402	SLEELREEALEALEELLELLGMSEAERHLARATLLLLLERHPELAPLLLKALRRLLEELK	60	D2
HE0409	ELKELIEKLIKKYKEISEKMLKELESMTSKEKLKEVLLKIIDLQAELDKEMVKTTFEILS	60	D2
HE0414	HKEELLKMVREMSEKIVKKIEELLKYLPEDTRKLVTELLKTLEELLKRAIEEGRVVEAVLALAALLILLALVLQQVEEEA	80	D2
HE0415	MEERVKVVEAAAELCRKRGLEEEAKLLEEAAELIKKGAKIEEITAKLLAAIKEAMKRGDILTAQALLAVHQLLLLLEQLD	80	D2

HE0416	MEERKKVVEALAEVCRKAGREEAAKLMEEAAKLIEEGASIEEITAKLLEAIKELMREGDLLLAQ AALAVHQLLLLLELLD	80	D2
HE0417	GEKIKELKEELKKKLEEAKKKVEEAEKEGWTLEKVAEIAELLTEALKIVIELLKLLLEQQ	60	D2
HE0419	SELLERLIKSLRELIELLRKQSKEIQILVIKTLIVNAVFSEEEAEKELERTEKEIKELLE	60	D2
HE0420	SEMKELSLEMLKLAVKMMKNLSVEKVAAILALMLAIAIIMGDEETAELAEEMLRKAIEMKKEGKS TEEVIKEIEKIIEEK	80	D2
HE0421	REKLIEEVTELFKKALEKLSLEEQELLFKALEATLKMAGKEEEAKLMKEAAKLLKEEIEA	60	D2
HE0423	EKVEEILKKIDEIEEKLVAKAKTELEAQAIRALTEATKLLILLNLELAKEAVKEFAKVLE	60	D2
HE0424	MKHNLRVLAKLAALAAVLAVGAMTALLLLAGFEEERKEELEKLKKIIEELEKKETIEEVLKLTLEE LEKGLKELKEEVEE	80	D2
HE0425	DKKKLIEAVLETAREISEKLKELAKLFEKAGEKDAYILVLQHLVNSKVNHAAILIKLGAEEEEVEKL VEEAVKAIKEALK	80	D2
HE0426	ELELKLKLLALINAATAKLVAMAKAILEKDLEEAAEEFKKLTAEVEALLKKAEELIKKAL	60	D2
HE0427	MAEERLLALHEERLELYREIGASNKELAIIALLLAVEALILGFEEVAKHYLELAEKLFKE	60	D2
HE0428	SRALQLLKENTCLTLLLLAVEEGAPEEAIEKLEKLLEELRKTGSTEALIAALKLTAEILE	60	D2
HE0429	MLEEILKLLRELEELVKDDPELTIAVLSLRIMIALKYDKEKAKEALEEFVKVVKEMLERE	60	D2
HE0432	MEIVELMAEAVKLLIEAGHYELVAALAETAALTAELVGVPEEAIELFRKAAELAKKRIKE	60	D2
HE0433	EERRRLLLQLAFLLRLARALSEFLDAASRERVEARIAELEARLPEEELAAAEEEVTRRSQTVEG QAELALEAAEALLDAV	80	D2
HE0434	SEKEEKLKKLAEEALELCETVKEKAIVALAFAIIALVLGYLEVAKEYLEKAKELLEKLLA	60	D2
HE0443	MEKLNEAYIKLLELLNEFQLTTTKLLLEGKLEELYKKLEELSKKINEAIEELLKLLKELI	60	D2
HE0444	EQALNDALVKLLELLNEFQLTTVKLYLEGKLEELYKKLEELSKKIQAATQELLALLKALI	60	D2
HE0448	KKVEIAKKAVREVAKVIKELLEELKDDIDFLEFVLALINSIVNKALLEVVESKLLGKSIDEVIKEVEK KEEEAIKRIEEV	80	D2
HE0449	EEAKKAAKEAAEAYKELIKLIDEGAAPEEIRAAVKKAQEKALELDTLIIDLGDPLLRAILRAKAEN NLSNTELALEVLEA	80	D2
HE0453	MEEAKEKMSKLLEEIAKIAKKNEDKEKQALIYLELAAIALILGDKELAKKFLEEALKILE	60	D2
HE0454	MKKLVEELAALVREIAKQLSPRDAALLLALLAAICLLVGDLETAEEAVEELKALLAKQKA	60	D2
HE0455	KEERVEELVREEDEYCEELEKEGKKLLAYLRRAAMCLVAALALLGYEEELKRLAEVLKRL	60	D2
HE0456	MVAKLAAAAVVLVLLLVPEEDRPEAVKLLEEILERVKAGESREELLKRAKELLDEALKEL	60	D2
HE0461	MKEAKLEFECGPASAVVATFTACLTTLLLKKPLDETIDIVKEVAKLLEEKLKEEEKVKVSITLKEE NGKRTLTITITEEL	80	D2

HE0482	DTKEKAETFAKIVAELVKQGFLETAAAFTREVLAELPEEDKLEVALAMTKAFLEELEKKE	60	D4
HE0485	EEEKQKELYKTIVESAKEISEKYGIDETVAQQQLLQAVALLSLLDEETIKRLTEEVLKMK	60	D4
HE0489	WEEKARRFLRIELIKELLHLYVLARVLGLEETAKLLAKAIKTLIEEDPESEKVLEEVKSLLNETGR EILERLLEEVEKEE	80	D3
HE0490	ELKKRVVRYVATRLFTEILSLEPLIGRETALELLLETARILYKASGELELILEVAREEMRRAGVPE EDIEALLAELRAWA	80	D3
HE0492	MEERLEKLNKLETVKEYLKKAVYLGTGDEVLEELLKYVPEEYKELILKVREAAELEKALL	60	D3
HE0497	SEEIAKDLRLMAEISHDNLATLAIILIREGEDIDFLLEIAEDLSELSRLTMRYVAEKLKA	60	D3
HE0499	KLLEVAVLKAIAELLGLAILDPRAIPLAREALEKLRKIHPSEIIKEMCDMGERILELIEE	60	D3
HE0501	KMAEMLVEHVILELERYEERGNAAAMIYTFIERLGKLALLGPEALRLAAAEFAKLPSPLAPELAA ACETAAALLERLLAA	80	D3
HE0502	KMAEMLVEHVILELEQYEARGNEAAMIYTFIEHLGKLALLGPEALELAAARLAKLPSPLAPELAA AAEEAAALLKRLLAA	80	D3
HE0505	DLLKEAEELVKKILETDPEANPAALNLYTILKTYVDIGAEKQAKKILELLKVVAEHLEKK	60	D3
HE0513	DAEALVERAAELLLERVRAGVDLEKALTEVLLAVLVRLTEEDAERFVELVVARFAETAELVRTLC EAALLKAKALVEALD	80	D3
HE0517	ERRERARLMMELLAEIAEEAAILQLAAQLAVLMYLSSLVGEDPEELLEIFEEIVLARVQTEFQRE VAERVLERLREFVEK	80	D3
HE0521	AEEVLALVAARVMRQLAEDALLAAELGLEEVAAALVELMKALAEEVAKRVSPEVAERVKRVLAL LLTRLEKEIAEAKAAE	80	D3
HE0537	SSFQEEAEKTFENLLELLLIATNLDTRVASELAEALVYIMGLLEENKEEFFEKFLKLSKYLNNVTL ARAIKTAIELGDKEFTEKLLKEFNKIIEEKKKLV	100	D4
HE0620	MKKIVIEAEMIPENHETILAVVKELVEEGVENLEIVLKVTPETTKEQSVELVKKIIEILK	60	C6
HE0621	AAAEIIKLNLTITENAKFQLLQDYAKFLGDKEELKKITEEANKWFEERMKELEERIKKLE	60	C6
HE0625	MVKRKFTLKLTTGASDDMTEVVPEFLAMIKEAAELFDEVTIKVTTESPEMARAVMEGVGILIKE GVDVTLEIELGSNVKARVEVLKTLAEELKKIKEEIE	100	C6
HE0626	MIKRHFTLKLTTGASDDMKEVVPEFLAMIKEAAELFDEVTLKLTTENPEMARALMEGVGILIKE GVDVHLEVELGSNVKARVEVLKTLAEELKKIKEEIE	100	C6
HE0633	ATTTTTTVTLDSASGERTLTTTTTVPAGTTLAEMVALAEAQLAAASEFDRRTATVTVTEE	60	C6
HE0639	SKAEELAKEMIELLRLVKELLLETTPEFPETTLIALAIVLNAVVTTLNALLDKVPLELLKELVEVMK DTVEGLKEKLKAL	80	C6
HE0644	MVSISLSGKTSEELKVMAKLAEYLGEEKTAEILKKLIPEVKELEAKGETSLEVSVTITFE	60	C6
HE0649	KKSVTIRVTADAAVLAEFTALLLEWLDLDAREVLEAFRKWLEEMKARGIKDDYTVELTVE	60	C6
HE0657	EELIKELKEKQKETQAELEKEYKEYKEKGESESAAIMKATSTQVATKYEIEIAKLKLELL	60	C6

HE0662	MEAILEAIRRLCEAGIKVTVVLAGPISLMEAIVETCIEVGVDEVVIDTSKDHLELIEADK	60	C6
HE0690	MLKVKIKVKDNPAVARGVLRLADKLKKAGVDVEIEIDLYGDEEQALATLAAMEAEVEELA	60	C8
HE0897	SAEALKLARLAALVGLVGAAVATADPANPLFLELLKNPDPFVLQEALTAALVAAILTGEAARCNAL ALGLAARLSREDAARAALRAAELAYETAALLASL	100	I
HE0898	SFTEEELEKLKEAVKLAVEALLLTPKDFEKALKLLEEVTLKVVEILRRDPLEALKAAFKSTTAIAKL YVAHASKDVSEAQAIAAEAVKALLDLYEKALKE	100	I
HE0899	SFTEEELEELKKAVKLAVEALLLTPDDFEKALKLLEEVTLRVVEILKRDPLEALKAAFYFNTQIAK LYVAHASGDVSEAQKEMAKFVKYLLDLYEKALEE	100	I
HE0900	AFTEEQLKALQESLKLVIEAAELMPDDFEKAIELVEEVARRLVEIFASDPLAALHLAFKFNTAIAK AIVANASKGKEEAMKVLVELAKYVFDLLVAALEA	100	I
HE0902	MFTEEEIKKIRESLKLSVEALEVTPKDFEKALELLEEVAINLMEIFKDDPMKALKIAFKFTNAIAKL YVAHESKDVADAMAIMAEVTKYILEILEKVLEE	100	I
HE0908	ELAEKERLARINIGAVLAGLAMAVVGACKELAEAGMVMAIMSVVAVELAQDPEAAVELAERCIE MAGGLSEEVKKMIKAAVRALKGVVEAEEAKIKKFKA	100	I
HE0915	EEELLEEILEFFEKLSDPKALVAFMLEKAPPDPGLKTALRIALSGPNITVGKALTLGKAIAEVSAG RYGGAGMIAGAAGGFEIGDEEVKRECKLALLKYL	100	I
HE0916	SKEVEEALLKALAAALIAAVAANKAAAVEVLRAIAEEGLDVLVAVVREAMRALADSPLVATLREA AREVAAELPPEERARFLAALEAAAAEVEAEAARAR	100	I
HE0917	SAEVEQALLEALAAALIGAVAASREAAKAVLRAIAELGLDVALAVVRRALRALAESPLVEVLLEA AREVAEELPPEERARFLAALARAAAEVEEEERERR	100	I
HE0918	EEAVKKALIEALAAALIAAVAASKEAAKAVLKAIAELGLDVAVEVVEKALRALADSPLVAVLIEAAK EVAEELPPEERARFLAALAEAVARVEAEAKEAA	100	I
HE0919	MEAVQQALLEALAAALIAAAAASAAAAKAVLRAIAELGLDVLVEVVRRALRALADSPLVATLLAA AREVAAELPPEERAEFLAALERAAAEVEEEAARAK	100	I
HE0920	SEEVRQALLEALAAALIAAAAASREAAVAVLRYLVELGLEVALEVVRLALRALRGSPLRERLLAA AEEVAAELPGEEAARFRAALARAAAEVAEEAAREE	100	I
HE0930	VKELVAELAELAGEADAELRAQIALLLAVIALAPEEQKEELAELALRVVREALEAAASPAVPVAL AAAAAAFARSEDPEAVRREAEAIITGLRLAKEGLA	100	I
HE0935	MKELLAEIAELAGEADAELRAQAALLLAVIALAPEEDKEFYAELALRVLRAGLEAAASPAVPVAL AKAAAALARGEDREAVERTIEAILTGLELTKKGLA	100	I

Supplementary methods

Metrics

Multiclass classification accuracy does not give an accurate estimate of performance in class imbalanced data settings as the majority classes dominate the performance. We instead evaluate all models on precision, recall, and F1-score that are all defined using

a fixed classification threshold of 0.5. For a given class, we consider all examples and count the number of correct and incorrectly classified ones like in a binary one-vs-rest classification setting. We further report Area Under the Precision-Recall Curve (AUC-PR) as it provides a comprehensive measure of the model's overall precision-recall trade-off at various thresholds. We also show the confusion matrices that give us a glimpse into which pairs of classes are harder for the model to disambiguate. Since it is infeasible to define “confusion” for examples with multiple labels, we restrict the confusion matrix analysis to structures with a single homo-oligomer symmetry label. These examples comprise roughly 6% of the structures in our dataset, 10% of the labels in the test set.

Maximum F1-score of all methods on validation data

Using the precision recall curves on the validation dataset (from Supplementary Figure 2), we compute the maximum F1-score achieved by each method shown in Supplementary Table 2. This gives us a sense of the best point on the P-R curves. We record the classification threshold at which the F1-score was maximum on the validation set and use this threshold to classify the held-out test dataset and report the F1-score at this threshold in Supplementary Table 3. Note that this threshold might not be the one that gives the highest F1-score once we compute the P-R curves in Supplementary Figure 3.

For the template-based baseline, which can only produce binary predictions (0 or 1 for each class), the “classifier threshold” is varied by varying the value “*k*” in the top-*k* hits considered as matches. As expected, smaller values of “*k*” result in a higher precision (for instance *k*=1 implies that we only consider the first template-based match that satisfies the sequence-identity cut-off) and higher values of *k* result in a higher recall at the cost of precision.

We find that the ESM2-based approaches have a higher F1-score overall (F1=0.63), with the finetuned models doing the best. However, the template-based approach has a strong performance too (F1=0.54) that is obtained at a higher recall albeit lower precision. This is in contrast to the fine-tuned models that generally have a higher precision and lower recall at the same or higher F1-scores.

Comparison of the QUEEN model on our dataset

To evaluate the pre-trained QUEEN [6] model which only considers the multiplicity, on our benchmark data, we collapse labels across different symmetry classes -- for example, the multiplicity 24 comprises proteins from C24, D12, and tetrahedral symmetry classes. Some symmetries cannot be mapped at all, such as the helical symmetry which has a variable multiplicity, or classes like C9 for which the pre-trained QUEEN model [6] does not predict. These examples are dropped from the validation

data. Additionally, multi-labeled examples have ambiguity in mapping and have been dropped. In total, we keep 88-92% of examples after this initial mapping and filtering step.

To prevent data leakage we removed proteins from our validation and test sets which were highly similar to those used to train the QUEEN model (30% identity and 80% coverage) [22]. We removed 16,403 proteins from validation and 34,575 from testing based on sequence similarity to QUEEN training data, leaving 9,941 and 23,014 proteins respectively in those sets. In Supplementary Figure 13, we show the performance of the QUEEN model both before and after the filtering step that removes the QUEEN training proteins. As expected, model performance drastically drops after data leakage is accounted for.

In Supplementary Table 8a,b and Supplementary Table 9a,b we show various metrics on the validation set, before and after filtering for sequence-similar proteins between our validation set of proteins and the QUEEN training dataset.

Training

The supervised models that use the pretrained feature embeddings were trained using sklearn's linear (`LogisticRegression`) and neural network classifiers (`MLPClassifier`). Since we operate in a multi-label multi-class classification setting, we use the `MultiOutputClassifier` wrapper class for converting the base classifiers to multi-output settings. The ESM-MSA and ESM2 models were fine-tuned using the pytorch lightning framework and RoseTTAFold2 was fine-tuned using pytorch's distributed data parallel framework.

Weighted data sampler for training

In order to prevent overfitting or "memorization" by the neural networks of the larger protein clusters that have thousands of proteins and to ensure that we sample a diverse set of proteins, we use a weighted data sampler where examples are inversely weighted based on cluster size.

Pretrained model hyper-parameter tuning

The hyper-parameters and regularization parameters were tuned on the validation set using `GridSearchCV`. For the neural network architectures, we try one and two hidden layers of various sizes.

Fine tuned model hyper-parameter tuning

To determine the optimal hyperparameters for the ESM class of models, we performed an iterative search in hyperparameter space to reduce model overfitting and address the challenge of using imbalanced data (i.e., substantially more C1/C2 training examples compared to rare symmetry groups). We assessed models based on Area Under the Precision-Recall Curve (PR-AUC).

We varied the L2 regularization parameter (we tried the following values: 0.1, 0.2, 0.5, 0.7), the dropout rate (values tried: 0.1, 0.2, 0.5, 0.7) used in the final symmetry group prediction layers of the model, the size and number of layers (values tried: 2,3,5) in the final symmetry group prediction head. We experimented with a multi-headed model architecture that had a separate set of trainable weights for each symmetry class or a single head for all symmetry classes. To reduce model overfitting in the MSA-based models, we also tried a random sequence selection step in the MSA construction step, a weighted sampler which assigned a higher weight to rare classes.

The best ESM-MSA model used high dropout and L2 terms (0.5 and 0.2 respectively), a separate prediction head for each symmetry group, and both random sequence selection for MSA construction as well as weighted sampling. The best Seq2Symm model used a dropout of 0.2, L2 regularization of 0.01, a learning rate of 5e-4, number of fine-tuned layers = 2, batch-size = 16, with weighted sampling and used the BCEWithLogits loss function. The Seq2Symm model trained with distillation data had a L2 regularization of 0.001, a learning rate of 1e-4, number of fine-tuned layers = 2, batch-size = 16, with weighted sampling and the margin-based loss function.

Other training settings

Our homo-oligomer dataset is class imbalanced due to the lack of PDB structures on proteins that have higher-order symmetries, and as such, training machine learning models that can learn most of the oligomer symmetries is challenging for all the models we experimented with (see Methods for how we address class imbalance). We find that the following improves validation set performance: and adding an auxiliary loss term involving a hierarchical loss defined on ‘coarse’ oligomer symmetries, where the higher-order C symmetries (C3 to C17) are grouped into a class ‘CX’ and D symmetries (D3 to D12) are grouped into a class ‘DX’. We sample a training batch based on proteins’ cluster membership (sequence-similar clusters) to increase the heterogeneity of structures seen in each batch.

Compute resources

To finetune RoseTTAFold2 we used an Azure VM that had 8 Tesla V100 gpus with 32gb gpu memory. For ESM2 and ESM-MSA we used 4 of these gpus for multi-gpu training using pytorch lightning.

Template-based method using HHSuite

We use the following parameters to run HHSearch while finding template-based matches to a protein of interest. The database used for the search is PDB version 03 March 2021, which includes all the proteins from our dataset.

```
hhsearch -hide_cons -hide_pred -hide_dssp -b 50 -B 1000 -z 50 -Z
500 -mact 0.05 -cpu 8 -maxmem 64 -aliw 100000 -e 0.001 -p 5.0 -d
<path-to-database>
```

No-homology split

We created another training setup where the training, validation and test splits are based on sequence homology defined by a relaxed sequence similarity threshold: e-value < 0.1. To create this split, we first use BLAST (blastp) to compute pairwise sequence similarity between all proteins in our dataset and then find connected components in this sequence similarity graph that define the "homologous clusters". We find one large cluster with 233,461 structures and 5,057 smaller clusters over the remaining 65,319 structures. The single large cluster is designated as the training split and we divide the remaining 5,057 clusters randomly into two sets to get the validation and test split. The number of structures and the class-wise distribution of the training / validation and test splits is shown in Supplementary Table 5.

MSA Analysis:

We analyze ~200 randomly selected MSAs from our test split and run inference using Seq2Symm on each protein sequence from the MSA. We show the diversity of predicted oligomer symmetries within each test MSA in Supplementary Figure 10a,c. Note that we do not use ground-truth symmetry labels for this analysis as we only have true symmetries for 0.01% to 5% of the sequences with an MSA (for large MSAs with hundreds or tens of thousands of sequences).

We perform a similar analysis on 300 randomly selected MSAs from our training split, plots for which are shown in Supplementary Figure 10b,d. Note that the query sequence from the train MSAs (i.e. the top sequence) is seen by Seq2Symm during training, i.e. the model has seen the true label for that sequence. However, we find that Seq2Symm assigns other labels to the orthologous protein sequences in each train MSA, given the number of unique predicted symmetries per MSA seen in Supplementary Figure 10b.

Generating all atom structures from Seq2Symm predictions:

We modeled 128 confident oligomer state predictions from the *E. coli* and *S. cerevisiae* proteomes. These were selected based on no overlap to the training dataset (BLAST e-value < 0.001), a total complex size of ≤ 3,000 residues, AFDB [35] monomeric structure model average pLDDT ≥ 75, and MSA depth ≥ 100 (after 95% identity, 50% coverage redundancy filtering). We find that 24 (19%) are confidently predicted by AlphaFold-multimer with pLDDT ≥ 75 and ipTM ≥ 0.5.

For the 128 structures, we compare the pLDDT, pTM, piTM values obtained using the symmetry predicted by Seq2Symm to those obtained using a random symmetry, only allowing random symmetries with a multiplicity between 2 to 6, to make the comparison more practical and for computational reasons (as against allowing a multiplicity of 20, which is very likely to have significantly different pLDDT values). We did a one-sided paired t-test to compare the two sets of scores and found those produced by Seq2Symm's symmetry to be significantly higher in all comparisons (pTM comparison with p-value: 6e-4, pLDDT comparison with p-value: 1e-3, piTM comparison with p-value of 1e-2).

95% sequence identity results:

We design this setting in order to simulate how a template-based method like HHSearch would be used to obtain homo-oligomer symmetry. Such approaches are not "trained" on a training split, and simply involve a template search against a database for a given query protein. However, our dataset includes many redundant examples due to the inherent redundancy of the PDB, which contains multiple structure entries for the same protein under different control conditions or with slight sequence variations (examples of redundancy: 102l_A,103l_A, 104l_A, 104l_B, 107l_A, 1078l_A etc.). Additionally, our dataset lists each chain from the protein as an individual example (ex: 2r15_A, 2r15_B). Hence, in order to evaluate HHSearch accurately, we need to restrict the use of identical structures to obtain labels on a given test protein.

Given these considerations we show performance of HHSearch and Seq2Symm on a train / test split created by MMSeqs clustering using 95% sequence identity at 90% sequence coverage – we call this the "95% setting". As before, the clusters are split randomly between a training and a test set. In this setup, structures for the same protein are unlikely to be split across the training and test split thereby avoiding obvious "substantial leakage". The Seq2Symm based models are trained on the training split, keeping the same hyper-parameters that were used to train the model on the "conventional split" (i.e. the validation split is not used). Each approach (including HHSearch) only has access to the training split and AUC-PR performance is evaluated on the test split. As before, the template baseline using HHSearch is run by varying the

number of hits considered from 1 to 80, to obtain AUC-PR. For HHSearch, no other restrictions are used on sequence identity.

We see the following macro-averaged test AUC-PRs: HHSearch with 0.542, Seq2Symm with 0.643. We show the class-wise AUC-PR on this “95% setting” below.

We think that different applications will present a model with inputs that are of varying “difficulty”. As expected, in an “easy” setting such as the “95% setting” that we show here, a trained deep learning model like Seq2Symm will do better, as we see with a test AUC-PR of 0.643.
